# Lysine benzoylation is a histone mark regulated by SIRT2

**DOI:** 10.1038/s41467-018-05567-w

**Published:** 2018-08-28

**Authors:** He Huang, Di Zhang, Yi Wang, Mathew Perez-Neut, Zhen Han, Y. George Zheng, Quan Hao, Yingming Zhao

**Affiliations:** 1 Ben May Department for Cancer Research, The University of Chicago, Chicago, IL 60637 USA; 2 School of Biomedical Sciences, University of Hong Kong, Hong Kong, China; 3 Department of Pharmaceutical and Biomedical Sciences, University of Georgia, Athens, GA 30602 USA

## Abstract

Metabolic regulation of histone marks is associated with diverse biological processes through dynamically modulating chromatin structure and functions. Here we report the identification and characterization of a histone mark, lysine benzoylation (K_bz_). Our study identifies 22 K_bz_ sites on histones from HepG2 and RAW cells. This type of histone mark can be stimulated by sodium benzoate (SB), an FDA-approved drug and a widely used chemical food preservative, via generation of benzoyl CoA. By ChIP-seq and RNA-seq analysis, we demonstrate that histone K_bz_ marks are associated with gene expression and have physiological relevance distinct from histone acetylation. In addition, we demonstrate that SIRT2, a NAD^+^-dependent protein deacetylase, removes histone K_bz_ both in vitro and in vivo. This study therefore reveals a new type of histone marks with potential physiological relevance and identifies possible non-canonical functions of a widely used chemical food preservative.

## Abstract (web summary)

Histone marks regulate chromatin structure and function. Here the authors identify and characterize lysine benzoylation, a histone mark that can be modulated by sodium benzoate, a widely used chemical food preservative, associated with specific regulation of gene expression.

## Introduction

Chromatin structure and transcriptional activity of genes are regulated by diverse protein posttranslational modifications (PTMs) in histones (or histone marks)^1,2^. Accumulating evidence shows that the status of histone mark levels is coupled to cellular metabolism^3–6^. As an example, short-chain fatty acids can be produced by cellular metabolism or derived from fermentation from the gut microbiota. These metabolites can serve as precursors for generation of acyl-CoAs that can be used for histone acylations^7,8^. Some examples of these metabolites include crotonate, butyrate, α-ketoglutarate, and 3-hydroxybutyrate^9^. The existence of a variety of different acyl-CoAs raises the intriguing possibility of there being undescribed pathways by which cellular metabolism impacts epigenetics through metabolite-directed histone marks.

Benzoyl-CoA is a central intermediate in the degradation of a large number of aromatic growth substrates in bacteria and gut microflora^10^. In mammalian cells, a likely source of benzoyl-CoA is sodium benzoate (SB), one of the most commonly used preservatives worldwide with a maximum allowed concentration up to 0.1% in foods. SB has been successfully used as a drug for patients with acute hyperammonemia, a result of diverse urea cycle disorders^11^. Although SB is listed among the “generally regarded as safe” compounds by the United States Food and Drug Administration, emerging studies have indicated that exposure to SB may cause harm to consumers^12–14^. Furthermore, inappropriate doses of intravenous SB to patients have led to severe complications^15^. These findings suggest human health risks are associated with SB; however, the underlying biological mechanisms remain unknown.

Here we report the histone mark lysine benzoylation (K_bz_). This histone mark has not been, to the best of our knowledge, previously described. We extensively characterize this new histone mark using a variety of chemical and biochemical methods, and detect 22 K_bz_ sites located on histones in mammalian cells. Metabolic labeling experiments indicated that SB could stimulate histone K_bz_ through the generation of cellular benzoyl CoA. Importantly, ChIP-seq (chromatin immunoprecipitation followed by sequencing) and RNA-seq results reveal that histone K_bz_ is a mark enriched in gene promoters and has unique physiological relevance. We further show that SIRT2, a NAD^+^-dependent protein deacetylase, can remove K_bz_ both in vitro and in vivo. Therefore, this study discovers an epigenetic mechanism that potentially contributes physiological changes caused by a widely used food preservative.

## Results

### Identification and verification of histone K_bz_

**Fig. 1:**
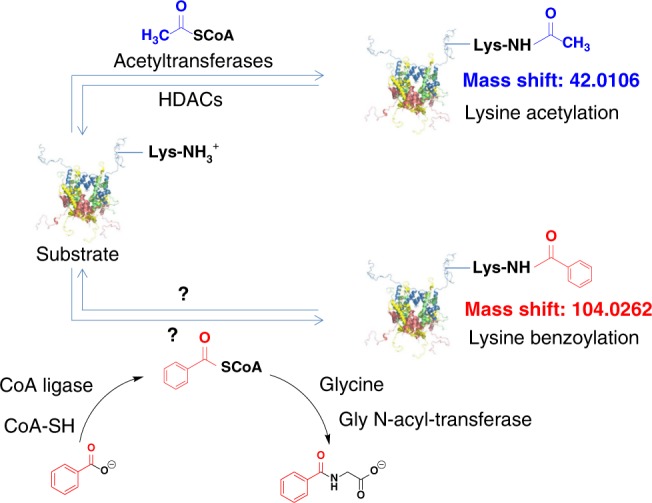
Lysine acetylation and benzoylation. Chemical structure of K_bz_ that induces a mass shift of +104.0268 Da is shown in red

To discover novel histone marks, we extracted core histones from HepG2 cells and performed tryptic digestion. The resulting tryptic histone peptides were subjected to high-performance liquid chromatography–tandem mass spectrometry (HPLC-MS/MS) analysis; the acquired MS/MS data were analyzed by the PTMap software with an unrestricted sequence alignment algorithm that enables searching mass shifts caused by PTMs^16^. Notably, a histone H2B peptide, PEPTK_+104.0268_SAPAPK, was identified with a mass shift of +104.0268 Da at the lysine residue position H2BK5 (Fig. 1). This accurate mass shift was used to deduce the possible element compositions with a maximum allowance of 2 nitrogen atoms and a mass tolerance of ±0.02 Da^17^. Based on the deduction results, the formula C_7_H_4_O is the most likely elemental composition, and only one reasonable chemical structure, benzoyl group, can be responsible for this formula (Fig. 1).

**Fig. 2:**
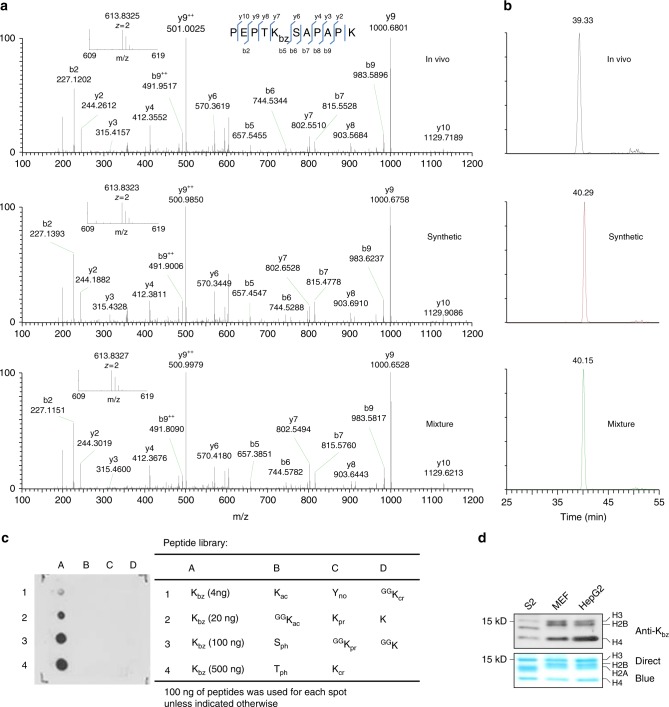
Identification and verification of histone K_bz_. **a** The MS/MS spectra of an in vivo peptide bearing a PTM (PEPTK_+104.0268_SAPAPK) (top), a synthetic lysine benzoylated peptide corresponding to the sequence of the in vivo peptide (middle), and a mixture of the two peptides (bottom). **b** Extracted ion chromatograms of the in vivo-derived peptide (PEPTK_+104.0268_SAPAPK) (top), the synthetic K_bz_ counterpart (middle), and their mixture (bottom) by HPLC-MS/MS analysis. **c** Dot blot assay of the pan anti-K_bz_ antibody. Peptide libraries were used for the assay. Each peptide library contains 10 residues CXXXXKXXXX, where X is a mixture of 19 amino acids (excluding cysteine), C is cysteine, and the sixth residue is a modified lysine residue as indicated. **d** Detection of K_bz_ in core histones from *Drosophila* S2 cells, MEF cells, and HepG2 cells

To confirm the deduced chemical structure of the mass shift, we synthesized a peptide that has the same sequence as the in vivo H2B peptide and contains a benzoyl group at H2BK5. MS/MS and coelution analysis were performed to compare the synthetic peptide with its in vivo counterpart (Fig. 2a, b). The results showed that the MS/MS spectra of two peptides matched very well and both peptides were coeluted. In addition, similar results were obtained from other two peptides, K_+104.0261_STGGK_ac_APR and K_+104.0260_QLATK_ac_AAR, from histone H3 in HepG2 cells (Supplementary Fig. 1). Theoretically, only chemically identical peptides can show the same MS/MS fragmentation patterns and HPLC retention times. Therefore, our MS/MS and coelution analysis confirmed that the mass shift of +104.0268 Da is caused by a new type of PTM, K_bz_.

Next, to further confirm K_bz_ and to detect in vivo K_bz_ sites, we generated a pan-anti-K_bz_ antibody. Pan anti-K_bz_ specificity was evaluated by a dot blot assay (Fig. 2c), in which the pan-anti-K_bz_ antibody can only recognize the peptides bearing a benzoyl lysine but not unmodified peptides or the peptides containing a broad spectrum of other PTMs, such as lysine acetylation (K_ac_), propionylation, crotonylation (K_cr_), tyrosine nitration, and serine/threonine phosphorylation. Using this antibody, we were able to detect K_bz_ histones from HepG2 cells, mouse liver, and *Drosophila* S2 cells (Fig. 2d). K_bz_ signals were detected among core histones from all the three species, indicating that K_bz_ is an evolutionarily conserved histone mark in mammalian and insect cells.

### Mapping histone K_bz_ sites in HepG2 and RAW cells

**Fig. 3:**
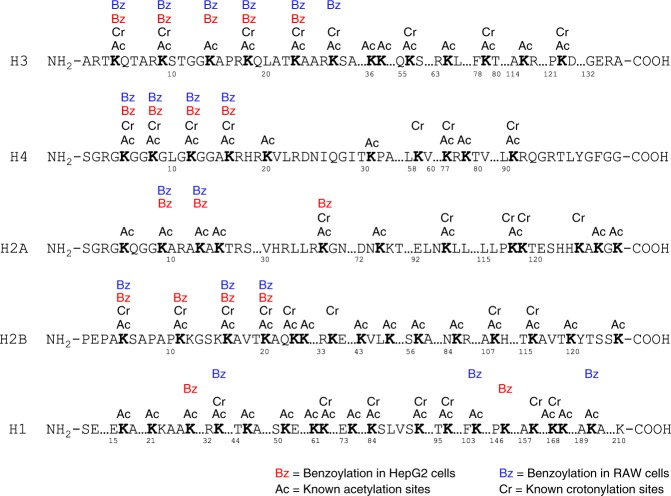
Illustrations of histone K_bz_ in mammalian cells. The detected K_bz_ sites in HepG2 and RAW cells are shown in red and blue, respectively. For comparison, the known K_ac_ and K_cr_ sites described in the literature are also listed

To identify in vivo histone K_bz_ marks, we carried out proteomic analysis using histone proteins extracted from HepG2 and RAW cells that were treated with 5 mM of SB. The extracted histones, with or without chemical propionylation, were tryptically digested and analyzed (see Online Methods). The spectra of the identified K_bz_ peptides were manually verified to remove false positives. Using these procedures and criteria, we identified 22 unique histone K_bz_ sites (Fig. 3, Supplementary Fig. 2, and Supplementary Table 1). The K_bz_ sites in HepG2 cells distributed in a similar pattern to those found in RAW cells. Interestingly, the 22 K_bz_ sites, either in HepG2 or in RAW cells, were mainly located on the N-terminal tails. In contrast, histone K_ac_ and K_cr_ sites are more widely spread, located in both N-terminal tails and other regions (Fig. 3). This line of evidence implies that K_bz_ may have a different role from histone K_ac_ and K_cr_ in chromatin regulation.

### SB stimulates K_bz_ by generating benzoyl CoA

**Fig. 4:**
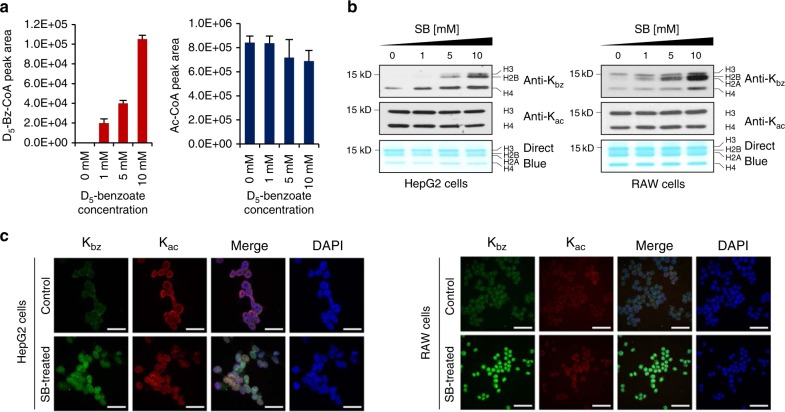
Dynamics of histone K_bz_ in eukaryotic cells. **a** HPLC-MS/MS analysis of cellular benzoyl-CoA levels extracted from HepG2 cells cultured with the indicated concentration of D_5_-SB (pH 7.4) for 24 h (*n* = 3, values are expressed as mean ± s.d.). **b** Western blotting analysis of core histone K_bz_ in response to SB with the indicated concentration in HepG2 (left) and RAW (right) cells. **c** The dynamics of K_bz_ and K_ac_ in response to SB treatment as determined by immunofluorescence staining using pan-K_bz_ and pan-K_ac_ antibodies. HepG2 (left) and RAW (right) cells were treated with 10 mM of SB for 24 h. Scale bar, 50 μm

Acyl-CoAs serve as donors for acylation reactions, in which the acyl groups in acyl-CoA are transferred to the ε-nitrogen of lysine residues by diverse acyltransferases. Recent studies showed that short-chain fatty acids, such as sodium crotonate, sodium succinate, and sodium malonate, stimulate production of their corresponding acyl-CoAs in cells, in turn elevating levels of histone acylations^9,18,19^. K_bz_ is chemically derived from SB; therefore, we speculate that SB may be used as a precursor to generate cellular benzoyl-CoA in vivo by a cellular lipid CoA synthetase, thereby stimulating histone K_bz_ level. To test this hypothesis, we metabolically labeled HepG2 cells with isotopic D_5_-SB for 24 h. Consistent with our hypothesis; D_5_-benzoylation was detected in histone peptides. In addition, the mass spectrometry-based quantification experiment showed an increase of D_5_-benzoyl-CoA in a dose-dependent manner upon D_5_-SB treatment (Fig. 4a and Supplementary Fig. 3).

To further test whether SB could be used by cells for K_bz_, we extracted histone proteins from the D_5_-SB-treated HepG2 cells and performed chemical propionylation for an aliquot of the histones. Both the chemical propionylated and non-propionylated histones were then tryptically digested and analyzed. Our experiment detected 15 histone K_bz_ sites bearing D_5_ (Supplementary Fig. 4 and Supplementary Table 2), further suggesting that SB is a precursor for the synthesis of benzoyl-CoA in the lysine benzoylation reaction.

### Dynamics of histone K_bz_ in response to SB treatment

To probe the dynamics of histone K_bz_ exposed to SB, we experimentally increased benzoyl-CoA level in both HepG2 and RAW cells by adding SB. Western blot analysis showed significant enhancement of K_bz_ on core histones after SB treatment, and we observed a dose-dependent increase in global histone K_bz_ levels both in HepG2 and in RAW cells (Fig. 4b). In contrast, the global histone K_ac_ levels slightly decreased in HepG2 cells and did not change in RAW cells upon SB treatment. These results were well confirmed by immunofluorescence staining, in which K_bz_ levels substantially increased, whereas no obvious changes were observed for K_ac_ levels (Fig. 4c).

To gain further insights into the dynamics of histone K_bz_ stimulated by SB, we quantified the changes of histone K_bz_ and K_ac_ peptides in response to SB treatment in both HepG2 and RAW cells (Table 1). In this experiment, equal amounts of extracted histones from SB-treated and control cells were chemically labeled with ^12^C- or ^13^C-propionic anhydride, respectively. Then the labeled histones were mixed, tryptically digested, and analyzed by mass spectrometry. Ratios of SB treated to control for K_bz_ and K_ac_ peptides were calculated basing on their precursor intensities. To eliminate potential bias caused by protein expression changes, all the ratios of quantifiable K_bz_ and K_ac_ peptides were normalized by corresponding histone protein expression levels. The results indicated that 5 mM (~0.07%) of SB, a concentration lower than the maximum allowed percentage in food, dramatically increased the abundance of histone K_bz_ (Table 1). For example, H3K23_bz_ and H4K8_bz_ sites in HepG2 cells, as well as H4K5_bz_ and H2AK13_bz_ sites in RAW cells, increased from 26.25- to 49.49-fold, while H2AK9_bz_ in both HepG2 and RAW cells increased more than 50-fold. Interestingly, 2 K_bz_ sites (H4K12_bz_ and H2AK13_bz_) in HepG2 cells and 2 K_bz_ sites (H3K14_bz_ and H4K8_bz_) in RAW cells were detectable only in SB-treated cells. Consistent with the western blot analysis, the levels of quantifiable K_ac_ sites in HepG2 cells decreased slightly, while the K_ac_ sites in RAW cells were stable in response to SB treatment. Notably, most of the lysine residues bearing dynamic K_bz_ modification are important to chromatin structure and function, suggesting important roles of K_bz_ in the regulation of chromatin functions.

**Table 1:** Quantified core histone K_bz_ and K_ac_ site dynamics in response to SB

Cell line	Site	Modified sequence	Ratio (T/C)
HepG2	H3K23_bz_	K_pr_QLATK_bz_AAR	27.38
HepG2	H4K8_bz_	GK_pr_GGK_bz_GLGK_ac_GGAK_ac_R	26.35
HepG2	H4K12_bz_	GK_pr_GGK_pr_GLGK_bz_GGAK_ac_R	Treated only
HepG2	H2AK9_bz_	GK_pr_QGGK_bz_AR	57.67
HepG2	H2AK13_bz_	AK_bz_AK_pr_TR	Treated only
HepG2	H3K27_ac_	K_ac_SAPATGGVK_pr_K_pr_PHR	0.75
HepG2	H4K8_ac_ H4K12_ac_ H4K16_ac_	GK_pr_GGK_ac_GLGK_ac_GGAK_ac_R	0.66
HepG2	H2AK5_ac_	GK_ac_QGGK_pr_AR	0.82
RAW	H3K14_bz_	K_pr_STGGK_bz_APR	Treated only
RAW	H4K5_bz_	GK_bz_GGK_pr_GLGK_ac_GGAK_ac_R	26.99
RAW	H4K8_bz_	GK_pr_GGK_bz_GLGK_pr_GGAK_ac_R	Treated only
RAW	H2AK9_bz_	GK_pr_QGGK_bz_AR	71.90
RAW	H2AK13_bz_	AK_bz_AK_pr_SR	49.69
RAW	H3K27_ac_	K_ac_SAPATGGVK_pr_K_pr_PHR	1.01
		K_ac_SAPSTGGVK_pr_K_pr_PHR	1.17
RAW	H2AK5_ac_	GK_ac_QGGK_pr_AR	1.01
		GK_ac_TGGK_pr_AR	0.87

The cells were untreated (C) or treated (T) with SB (5 mM, pH 7.4) for 24 h. Extracted histones from untreated and treated cells were chemically propionylated by ^13^C- and ^12^C-propionic anhydride, respectively. Ratios were normalized by the corresponding protein expression levels

Given that K_ac_ is the most abundant acylation in cells, next we compared the relative abundance of K_bz_ with K_ac_ by spectral counting. To this end, we performed immunoprecipitation experiments using histones extracted from SB-treated (5 mM for 24 h) HepG2 or RAW cells. Pan anti-K_ac_ and pan anti-K_bz_ antibodies were used to enrich K_ac_ and K_bz_ peptides. The results indicated that K_bz_ levels on some sites are equivalent or close to corresponding K_ac_ levels (Supplementary Fig. 5). For example, K_bz_ levels on H2AK5, H2AK8, and H3K9 are as high as K_ac_ levels, while K_bz_ levels on H3K14 and H4K5 are higher than the half of corresponding K_ac_ levels either in HepG2 or in RAW cells, indicating the high abundance of K_bz_ in cells exposed to SB.

### SIRT2 removes histone K_bz_ both in vitro and in vivo

Histone deacetylases (HDACs) are a family of annotated enzymes that can remove acetyl groups from acetylated substrate proteins^20^. One of the HDACs, SIRT5, was identified as an NAD^+^-dependent demalonylase, desuccinylase, and deglutarylase instead of deacetylase^18,21,22^, suggesting that some HDACs may have non-canonical enzymatic activities and thus may work on histone K_bz_.

To identify potential K_bz_ deacylases, we screened all HDACs (including HDACs 1–11 and Sirtuin 1–7) using the synthetic peptide PEPTK_bz_SAPAPK as a substrate. HPLC-MS/MS analysis showed that only SIRT2 exhibited significant lysine debenzoylation activity (Supplementary Fig. 6a). These results are consistent with our western blot assays in which core histone K_bz_ levels of HepG2 and RAW cells did not visibly change following treatment with a class I/II HDAC inhibitor, Trichostatin A (TSA), even though the core histone K_ac_ levels increased obviously under the same conditions (Supplementary Fig. 6b).

**Fig. 5:**
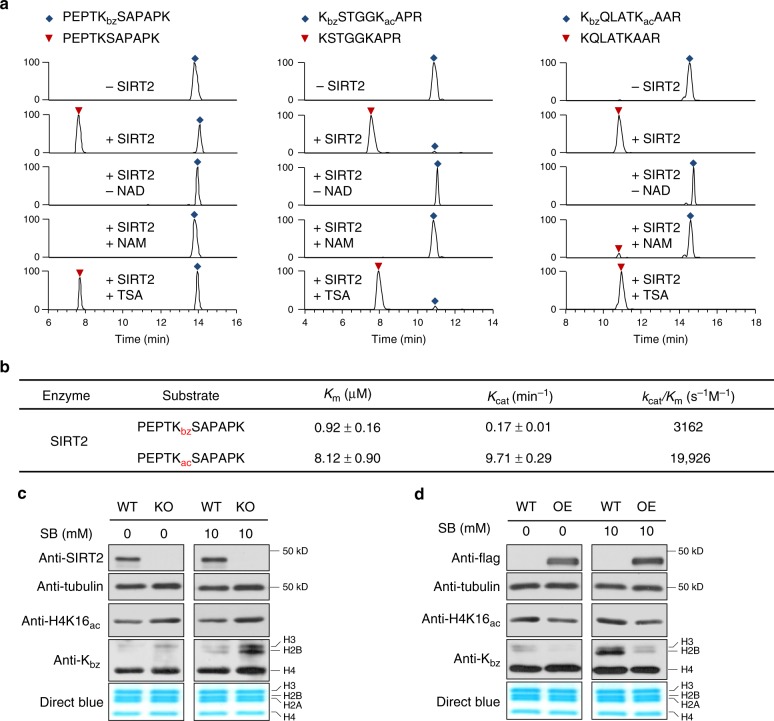
SIRT2 catalyzes histone lysine de-benzoylation both in vitro and in vivo. **a** SIRT2 can catalyze in vitro debenzoylation reactions of 3 synthetic K_bz_ peptide substrates. **b** Kinetics data for SIRT2 on acetyl and benzoyl histone H2AK5 peptides. **c** Core histone K_bz_ increased in SIRT2 knockout (KO) MEF cells. Whole-cell extracts were probed for the presence of SIRT2 and tubulin by western blot. Acid-extracted histones were tested for H4K16_ac_ and global K_bz_ levels by western blot. Total histones levels were visualized with Coomassie blue staining. **d** Core histone lysine benzoylation decreased in SIRT2 overexpressing (OE) cells. Flag-SIRT2 was transfected into HEK293T cells for 48 h. Whole-cell extracts were probed for the presence of Flag-SIRT2 and tubulin by western blot. Acid-extracted histones were tested for H4K16_ac_ and global K_bz_ levels by western blot. Total histones levels were visualized with Coomassie blue staining

To confirm the direct debenzoylation activity of SIRT2, we carried out in vitro debenzoylation reactions using two additional synthetic peptides bearing benzoylated lysine residues (K_bz_STGGK_ac_APR and K_bz_QLATK_ac_AAR) as substrates (Fig. 5a). In both cases, we detected their corresponding unmodified counterparts. In contrast, the debenzoylated peptides were not detected in the absence of NAD^+^, an essential cofactor for this reaction. Furthermore, this reaction was inhibited by the class III HDAC inhibitor nicotinamide but not by TSA. These results demonstrate that SIRT2 is a debenzoylase in vitro.

Given that SIRT2 is a known deacetylase, we next quantitatively compared its deacylase activity toward acetyl and benzoyl peptides. We performed kinetic studies using H2BK5_ac_ or H2BK5_bz_ peptides, respectively. The results showed that the *K*_m_ for deacetylation was ~8.8-fold higher than that for debenzoylation, while the turnover number (*K*_cat_) for deacetylation was ~57.1-fold higher than that for debenzoylation (Fig. 5b). Therefore, *K*_cat_/*K*_m_ for debenzoylation was approximately 1/6 of that for deacetylation.

We next examined whether SIRT2 has debenzoylase activity in vivo. By examining core histone K_bz_ levels in *Sirt2*^*+/+*^ (wild-type (WT)) and *Sirt2*^*−/−*^ (knockout (KO)) mouse embryonic fibroblast (MEF) cells, we found that loss of SIRT2 expression was associated with increased K_bz_ levels, especially on H3 and H2B proteins (Fig. 5c). In contrast, overexpression of SIRT2 in HEK293T cells by transient transfection led to decreased global K_bz_ levels (Fig. 5d). Interestingly, these effects were significantly amplified by SB stimulation, while the levels of the H4K16_ac_ positive control were unchanged following SB stimulation^23^. Collectively, these data indicate that SIRT2 is a debenzoylase both in vitro and in vivo.

To further understand the debenzoylase activity of SIRT2, we performed in silico molecular modeling to investigate the binding interaction between a benzoylated substrate and SIRT2. The modeling results showed that benzoyl group on lysine side chain reached to the catalysis pocket center (Supplementary Fig. 6c). Similar to myristoyl substrate^24^, the extensive hydrophobic interactions between benzoyl substrate and SIRT2 led to stronger binding energy than corresponding acetyl substrate (Supplementary Fig. 6d) and may account for much lower *K*_m_ value of SIRT2 toward myristoyl or benzoyl peptides than acetyl peptide.

### Genome-wide mapping of histone K_bz_ in HepG2 cells

**Fig. 6:**
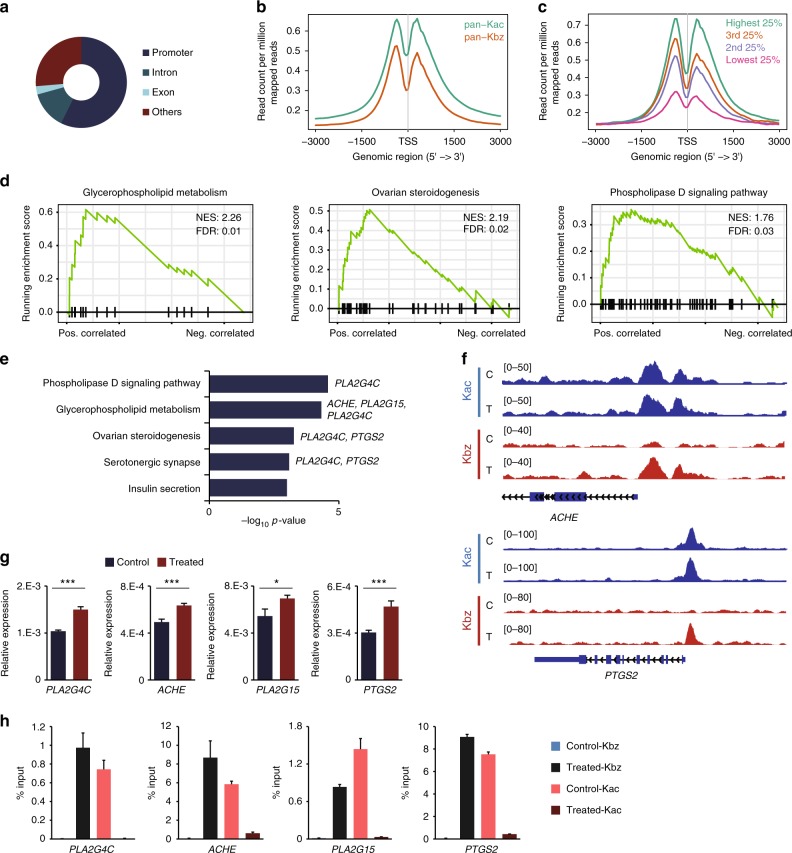
Genome-wide localization and physiological relevance of histone K_bz_ in HepG2 cells. **a** Genome-wide distribution of histone K_bz_ in SB-treated HepG2 cells. **b** Curves showing average profile of histone K_bz_ and K_ac_ ChIP-seq read counts (per million mapped) around all known TSSs. **c** All genes were split into four equal groups based on their expression levels calculated from RNA-seq data. The average profiles of histone K_bz_ ChIP-seq read counts (per million mapped) for each group were plotted at all known TSSs. **d** KEGG pathway analysis of RNA-seq data using GSEA. **e** KEGG pathway analysis of selected genes. The genes selected for validation in each pathway were labeled. **f** A representative snapshot showing the normalized ChIP-seq reads for K_bz_ and K_ac_ at *ACHE* and *PTGS2* genes (C: control, T: SB-treated). **g** RT-qPCR analysis of the control and SB-treated HepG2 cells. Relative expression is normalized to *ACTIN*. *p < 0.05, ***p < 0.001 (*n* = 4, unpaired Student’s *t* test, values are expressed as mean ± s.d.) compared to control conditions. **h** qPCR analysis of K_bz_ and K_ac_ ChIP products from the control and SB-treated HepG2 cells (*n* = 3, values are expressed as mean ± s.d.)

In order to investigate the genome-wide distribution of histone K_bz_ mark, we carried out ChIP-seq with the pan anti-K_bz_ antibody in HepG2 cells, also including the pan anti-K_ac_ antibody as a control. In total, we identified 20283 K_bz_ peaks distributed in 13,255 genes in SB-treated HepG2 cells. Analysis of the ChIP-seq datasets revealed that more than half (57.33%) of K_bz_ signals were associated with gene promoters (defined as ±3 kb around the transcription start sites [TSSs]; Fig. 6a). Similar to K_ac_, K_bz_ shows depletion in TSSs but is enriched around TSS regions, with slightly higher signals in upstream compared to downstream (Fig. 6b). These lines of evidence suggest that histone K_bz_ may be associated with transcription.

We therefore examined the association between histone K_bz_ mark and gene expression. To this end, we classified all genes into four categories based on their expression levels and plotted ChIP-seq peak signals of K_bz_ on these genes. Interestingly, we observed a positive correlation between K_bz_ levels and gene expression at TSSs (Fig. 6c), which strongly support a role of histone K_bz_ in gene expression.

### Profiling physiological relevance of histone K_bz_ mark

To explore the epigenetic role of histone K_bz_, we first analyzed RNA-seq datasets from control and SB-treated HepG2 cells. In this analysis, quantified genes (false discovery rate (FDR) < 0.05) with the presence of K_bz_ ChIP-seq peaks (FDR < 0.01 using magnetic-activated cell sorting (MACS)) in TSSs were selected. Then gene set enrichment analysis (GSEA) was used to compare the rank-ordered dataset of SB-treated versus control transcripts with respect to the KEGG pathways (Supplementary Data 1). The results showed that elevated K_bz_ is positively correlated with glycerophospholipid metabolism, ovarian steroidogenesis, and phospholipase D signaling pathways (Fig. 6d).

To examine whether the upregulated genes in these pathways were regulated by K_bz_ but not K_ac_, we performed an additional KEGG pathway enrichment analysis using the ChIP-seq datasets (Supplementary Data 2). In this analysis, genes were selected based on three restrictive criteria: (1) the presence of K_bz_ peaks (FDR < 0.01 using MACS) in TSSs with increased ChIP-seq read counts after SB treatment; (2) the presence of K_ac_ peaks (FDR < 0.01 using MACS) in TSSs with decreased or unchanged ChIP-seq read counts after SB treatment; and (3) transcripts upregulated (FDR < 0.05 and at least 0.5 absolute log2 fold change) after SB treatment. This analysis identifies five pathways (*q* < 0.05, Fig. 6e): phospholipase D signaling, glycerophospholipid metabolism, ovarian steroidogenesis, serotonergic synapse, and insulin secretion. Among the five pathways, three were also identified in the RNA-seq GSEA analysis. These results confirmed that histone K_bz_ marks are specifically located in chromatin regions that are associated with gene expression.

Next, we choose four genes to validate the correlation between histone K_bz_ and gene expression by reverse transcriptase quantitative polymerase chain reaction (RT-qPCR) and ChIP-qPCR. The selected genes for validation were derived from the phospholipase D signaling, glycerophospholipid metabolism, ovarian steroidogenesis, and serotonergic synapse pathways, which were affected by increased K_bz_ levels. These four genes are *PLA2G4C*, *ACHE*, *PLA2G15*, and *PTGS2*. A representative snapshot of the normalized ChIP-seq reads of selected genes *ACHE* and *PTGS2* is shown in Fig. 6f. RT-qPCR results showed that expression of all the selected genes, which were marked by increased K_bz_ but decreased K_ac_, were upregulated with 1.28- to 1.55-fold increase (*PLA2G4C*: 1.44-fold, *ACHE*: 1.29-fold, *PLA2G15*: 1.28-fold, and *PTGS2*: 1.55-fold) (Fig. 6g). ChIP-qPCR results confirmed that the K_bz_ levels at the promoters of selected genes increased after SB treatment, while the corresponding K_ac_ levels decreased (Fig. 6h), indicating that the upregulation of these genes were associated with K_bz_ instead of K_ac_. Taken together, these data confirmed the correlation between histone K_bz_ dynamics and gene expression, supporting potential physiological relevance of histone K_bz_ mark and its role in the regulation of gene activity.

## Discussion

Emerging evidence suggests that short-chain histone lysine acylations are physiologically relevant and contribute to the regulation of chromatin structure and gene expression^1,17,25^. Despite the structural similarity, they are regulated by very different metabolic pathways and associated with unique physiology and diseases. K_bz_ is structurally very different from the other short-chain lysine acylations. To date, K_bz_ is, to the best of our knowledge, the only known histone lysine PTM bearing an aromatic acyl group. Compared with other histone marks bearing the short-chain fatty acids, K_bz_ has lager molecular volume and stronger hydrophobicity (Supplementary Table 3). For example, the benzoyl group is more than two times the size of acetyl group, and the predicted log *P* (logarithm of octanol–water partition coefficient) of benzoyl group is about three-fold higher than that of acetyl group. In eukaryotes, histone acylations constitute an important epigenetic mechanism that regulates gene expression patterns by loosening or tightening the interaction between DNA and histones. Therefore, we anticipate that histone K_bz_ might have a more significant structural impact than K_ac_ and lysine methylation on chromatin structure. In addition, due to the significant structural difference between K_bz_ and other histone marks, it is tantalizing to speculate the possible existence of specific proteins or domains that recognize K_bz_ and cause diverse downstream alterations in chromatin, providing an additional layer of control over gene transcription^26^. Furthermore, like other lysine acylations, K_bz_ mark may be spread widely throughout the proteome and have important functions on non-histone proteins as well.

Our preliminary studies show that histone K_bz_ has unique regulatory enzyme profiles. Histone K_ac_ and a few other short-chain lysine acylations can be removed by multiple HDACs, such as HDAC1–3, HDAC6, and SIRT1–3^27,28^. In contrast, SIRT2 is the only HDAC that can remove K_bz_ in our in vitro screen. These findings suggest the K_bz_ pathway has a unique regulatory mechanism. Given the epigenetic roles of SIRT2 in diverse cellular processes^29–31^, this study not only opens an opportunity for revealing unknown cellular mechanisms controlled by SIRT2 but also sheds some light on the epigenetic roles of K_bz_.

The ChIP-seq and RNA-seq analyses revealed that the histone K_bz_ mark, rather than the well-known K_ac_, is involved in the expression of specific genes associated with glycerophospholipid metabolism, ovarian steroidogenesis, and phospholipase D signaling pathways. Thus histone K_bz_ and K_ac_ marks can be associated with distinct sets of genes. It has been reported that some lysine acylations, such as K_ac_ and butyrylation (K_bu_), or K_ac_ and 2-hydroxyisobutyrylation (K_hib_), can co-localize in the same genomic regions. In addition, the co-localization, such as histone H4 K5K8 acetylation and butyrylation, can act synergistically to enhance transcription activity in certain biological processes, such as sperm cell differentiation^32^. Therefore, the molecular mechanisms by which histone K_bz_ exerts its functions are likely different from those utilized by histone K_bu_ and K_hib_ sites in transcriptional regulation, although a clear physiological role for this modification still needs to be established in further studies.

Finally, we demonstrate that 5 mM (~0.07%) of SB can significantly increase histone K_bz_ levels, which could pose potential safety concerns, as this concentration is lower than the maximum allowed percentage (0.1%) in food. Human plasma SB concentration can be as elevated as ~10 mM in patients with hyperammonemia receiving high doses of intravenous SB, which resulted in severe complications and showed toxicity of SB^15^. In addition, oral administration of high-dose SB increases the plasma SB concentration in healthy male volunteers in the range of 2–4 mM^33^. These concentrations of SB in plasma are similar to or even exceed the concentrations that we used for cell treatment and could be associated with transcriptional responses. Moreover, it is becoming increasingly accepted that acyl-CoAs can be generated at local intracellular compartments, directly contributing to the PTM of proximal substrates^34^. Therefore, the concentration of benzoyl-CoA generated in proximity to chromatin could exceed the average cellular concentrations and could facilitate the addition of K_bz_ to histones. These data suggest that the K_bz_ mark could exist at physiologically relevant levels in cells. In fact, several studies in the past decade have suggested that SB could cause harmful effect on human health^12–14^. However, the underlying biological mechanism for SB function remains unclear. Thus our study not only discovers an epigenetic and PTM pathway but also sheds light on potential mechanisms for the physiological changes induced by SB.

## Methods

### Materials and reagents

Unless otherwise noted, all chemical reagents were purchased from Sigma-Aldrich (St. Louis, MO). D_5_-SB was from C/D/N Isotopes Inc., D-288. Antibodies were the following: anti-pan K_ac_ (1:1000, PTM Biolabs, PTM-101), anti-H4K16_ac_ (1:15,000; PTM Biolabs, PTM-122), anti-H3 (1:5000, Abcam, ab1791), anti-H4 (1:5000, Abcam, ab31830), anti-Flag (1:10,000; Sigma-Aldrich, F7425), anti-Tubulin (1:10,000; Abcam, ab6160), and anti-SIRT2 (1:1000, Abcam, 23886). Anti-pan K_bz_ antibody (1:1000) was made by PTM Biolabs Inc. (Chicago, IL). MEFs derived from WT and *Sirt2* KO mice are kind gifts of Dr. Chu-Xia Deng (NIH)^35^. HepG2 (HB-8065), RAW (TIB-71), and 293T (CRL-3216) cell lines were purchased from ATCC (www.atcc.org) and used without further authentication. No mycoplasma contamination was detected using a MycoAlert™ Mycoplasma Detection Kit (Lonza, LT07-118).

### Western blot analysis

Protein extracts (20 µg of whole-cell proteins or 2–4 µg of histones) were fractionated by sodium dodecyl sulfate-polyacrylamide gel electrophoresis and transferred to a polyvinylidene difluoride membrane using a transfer apparatus according to the manufacturer’s protocols (Bio-Rad, Hercules, CA). After incubation with 3% bovine serum albumin (BSA) in TBST (10 mM Tris, pH 8.0, 150 mM NaCl, 0.5% Tween 20) for 1 h, the membrane was incubated with the indicated primary antibody (concentration is shown in “Materials and reagents” section) at 4 °C overnight. Then the membrane was washed three times (10 min for each) with TBST and incubated with a 1:20,000 dilution of horseradish peroxidase-conjugated anti-mouse or anti-rabbit antibodies at room temperature for 1 h. Next, the membrane was washed three times (10 min for each) with TBST and developed with enhanced chemiluminescence detection system (Thermo Scientific, Rockford, IL) according to the manufacturer’s protocols. Uncropped western blots are shown in Supplementary Figures 7–9.

### Chemical propionylation

Two hundred fifty µg of histone sample was dissolved in 250 μL of 0.1 M NH_4_HCO_3_ buffer (pH = 8.0), followed by adding 3.0 μL propionic anhydride and then adjusting pH to 8–9 with instant monitor. After 1 h incubation at room temperature, we added another 3.0 μL of propionic anhydride and kept the pH at 8–9 by addition of 1 M NaOH. The mixture was incubated at room temperature for another 1 h and the reaction was blocked by adding 3.0 μL of 2-aminoethanol. The mixture was incubated for 15 min and digested with trypsin (histone: trypsin = 50:1) at 37 °C overnight.

### CoA extraction

Cell culture media was quickly removed and the dish was placed on top of dry ice. Then 1 mL of extraction buffer (80% methanol/water, v/v) was immediately added and the dishes were transferred to a −80 °C freezer. The dishes were left at −80 °C for 15 min and transferred on top of dry ice. Next, cells were scraped into extraction solvent and the solution was centrifuged with the speed of 20,000 × *g* at 4 °C for 10 min. The supernatant was dried in Speed Vac (ThermoFisher Scientific, San Jose, CA) for HPLC-MS/MS analysis.

### Immunoprecipitation

The peptides in NH_4_HCO_3_ solution were incubated with prewashed pan anti-K_bz_ beads (PTM Biolabs Inc., Chicago, IL) at 4 °C overnight with gentle shaking. After incubation, the beads were washed three times with NETN buffer (50 mM Tris pH 8.0, 100 mM NaCl, 1 mM EDTA, 0.5% NP40), twice with ETN buffer (50 mM Tris pH 8.0, 100 mM NaCl, 1 mM EDTA), and once with water. The bound peptides were eluted from the beads with 0.1% trifluoroacetic acid, and the eluted fractions were combined and vacuum-dried.

### Immunofluorescence staining

HepG2 and RAW cells were seeded on coverslips before experiment. The cells on coverslips were washed twice with phosphate-buffered saline (PBS, 137 mM NaCl, 2.7 mM KCl, 10 mM Na_2_HPO_4_, and 2 mM KH_2_PO_4_) and fixed in 4% paraformaldehyde at room temperature for 15 min. After rinsing with PBS twice, the coverslips were incubated with 0.5% Triton X-100 at room temperature for 10 min, blocked with 3% BSA at room temperature for 60 min, and incubated with mouse pan anti-K_ac_ (1:100) antibody and rabbit pan anti-K_bz_ (1:100) antibody mixture at 4 °C overnight. The coverslips were washed twice with PBST (PBS with 0.1% Tween 20), followed by incubation with Alexa Fluor 488-conjugated and Alexa Fluor 594-conjugated secondary antibodies (1:100, against rabbit and mouse, respectively) at room temperature for 60 min. Next, the cells were counterstained with 4,6-diamidino-2-phenylindole and mounted onto glass slides. Images were acquired with a Leica SP5 confocal microscope system.

### HDAC screening

The reactions were performed in a final volume of 50 μL per well in a 96-well microplate. For each reaction, 0.5 μM of K_bz_ peptide and HDACs (0.08 μM of HDAC1–11 or 0.25 μM of SIRT1–7) was added to reaction buffer (for HDAC1–11: 25 mM Tris pH 8, 130 mM NaCl, 3.0 mM KCl, 1 mM MgCl_2_, 0.1% PEG8000, pH 8.0; for SIRT1–7: 20 mM Tris pH 8, 1 mM DTT, 1 mM NAD^+^) in sequence. After 30 min incubation at 37 °C, the reactions were stopped by adding 50 μL of 200 mM HCl and 320 mM acetic acid in methanol. The samples were dried and analyzed by HPLC-MS/MS with a gradient of 5–90% HPLC buffer B (0.1% formic acid in acetonitrile, v/v) in buffer A (0.1% formic acid in water, v/v) at a flow rate of 900 nL/min over 15 min. Product quantification was based on their peak areas. The *K*_cat_ and *K*_m_ values were obtained by curve fitting the 1/*V*_initial_ versus 1/[S].

### Molecular docking

Three-dimensional structure of SIRT2 (PDB ID 4Y6L) was downloaded from www.rcsb.org. Ligands were constructed with PyMol basing on the myristoyl peptide structure in PDB 4Y6L. Three residues of the ligand peptide, Arg-Lys-Ser, were kept, in which lysine was modified by different acylation groups. All the ligands were energy minimized using Chimera^36^. Receptor and ligand files for docking simulation were prepared using Autodock tools^37^ (version 1.5.6). AutoDock vina^38^ (version 1.1.2) program was employed for docking using protein and ligand information along with grid box properties in the configuration file. Grid center was designated at *x* = 6.866, *y* = −13.796, and *z* = 39.416, and the box size was set at *x* = 30, *y* = 16, and *z* = 16. Each docking simulation was run with the number of modes set to 9 and energy range set to 4. The docking results were manually checked and the pose with the lowest energy of binding was extracted. Binding detail of each ligand was further analyzed with LigPlot^+^ 1.4 program.

### HPLC-MS/MS analysis

Histone peptide samples were dissolved in 2.5 µL of HPLC solvent A (0.1% formic acid in water, v/v) and loaded onto an in-house packed capillary C_18_ column (10 cm length×75 µm ID, 3 µm particle size, Dr. Maisch GmbH, Ammerbuch, Germany), which was connected to EASY-nLC 1000 UHPLC system (ThermoFisher Scientific, San Jose, CA). Peptides were separated with a gradient of 5–90% HPLC buffer B (0.1% formic acid in acetonitrile, v/v) in solvent A at a flow rate of 200 nL/min over 60 min. The eluted peptides were analyzed by a Q Exactive mass spectrometer (ThermoFisher Scientific, San Jose, CA) using a nano-spray source. Full mass scans were acquired in the *m*/*z* range of 300−1400 with a mass resolution of 70,000 at *m*/*z* 200. The 15 most intensive ions were fragmented with 27% normalized collision energy and tandem mass spectra were acquired with a mass resolution of 17,500 at *m*/*z* 200.

The extracted CoA samples were separated with 90% HPLC buffer B (5 mM NH_4_OAc in 95/5 ACN/H_2_O, v/v) in buffer A (5 mM NH_4_OAc) at a flow rate of 900 nL/min over 20 min. The eluted molecules were analyzed by an Orbitrap Velos mass spectrometer (ThermoFisher Scientific, San Jose, CA) with a targeted MS/MS method. Full mass scans were acquired with a resolution of 30,000 at *m*/*z* 400; isolation width of precursor ions was set at *m*/*z* 3.0, and high-energy collision dissociation was set at 35%^39^.

### Protein sequence database searching

Unrestrictive identification of PTMs was performed with PTMap algorithm. Mass shift of potential protein modifications ranged from −50 to +200 Da. Maximum missing cleavage was set at 3, and mass tolerance was set at ±0.01 Da for precursor ions and ±0.5 Da for MS/MS. Restrictive identification was performed with the Mascot search engine (Matrix Science, London, UK) against UniProt Human protein database (88,277 entries, http://www.uniprot.org). The following parameters were used during sequence alignment: methionine oxidation, protein *N*-terminal acetylation, lysine acetylation, lysine mono-/di-/tri-methylation, arginine mono-/di-methylation, and lysine benzoylation were specified as variable modifications. Maximum missing cleavage was set at 4, and mass tolerance was set at 10 ppm for precursor ions and ±0.05 Da for MS/MS. Propionylated histone samples for quantification were searched using MaxQuant v1.3.0.5 with integrated Andromeda search engine. Tandem mass spectra were searched against UniProt Human protein database (88,277 entries, http://www.uniprot.org) concatenated with reverse decoy database. Trypsin/P was specified as cleavage enzyme allowing up to four missing cleavages. Methionine oxidation, protein *N*-terminal acetylation, lysine acetylation, lysine mono-/di-/tri-methylation, arginine mono-/di-methylation, and lysine benzoylation were specified as variable modifications. FDR thresholds for protein, peptide, and modification site were specified at 1%. K_bz_ identified on peptides from reverse or contaminant protein sequences and peptides with Andromeda score <40 were removed. All the K_bz_ site ratios were normalized by the quantified protein expression levels.

### ChIP-seq analyses

HepG2 cells were grown in full media with or without SB treatment (10 mM for 24 h). Chromatin was prepared by MNase digestion^40^. For each immunoprecipitation, 3 µg of pan anti-K_bz_ or anti-K_ac_ antibody were incubated with 30 µg chromatin overnight at 4 °C. ChIP-seq libraries were prepared following the TruSEq Chip-SEQ Kit (Illumina, San Diego, CA) as per the manufacturer’s instruction. The libraries were sequenced with 50 bp single read sequencing on Illumina HiSeq 2500 machine at the University of Chicago Genomics Core as per the manufacturer’s protocols. ChIP-seq reads were mapped to reference genome of Illumina iGenomes UCSC hg38 using Bowtie^41^ (version 2.2.6), and only the uniquely mapped reads were retained. SAMtools^42^ (version 0.1.19) was then used to convert files to bam format, sort, and remove PCR duplicates. Ngs.plot^43^ (version 2.61) was used to generate average profiles of ChIP-seq reads. Peaks were called using MACS^44^ (version 2.1.1) with FDR = 0.01, and data were visualized using IGV^45^ (version 2.4). The differential binding analysis and KEGG Pathway analysis was implemented using DiffBind^46^ and clusterProfiler^47^ packages, respectively.

### RNA-seq analyses

Total RNAs were extracted from control and SB-treated (10 mM for 24 h) HepG2 cells using the RNeasy Mini Kit (QIAGEN Inc, Valencia, CA). Four biological replicates were performed for each studied condition. The sequencing libraries were prepared using the TruSeq Stranded Total Sample Preparation Kit (Illumina, San Diego, CA) as per the manufacturer’s instruction. The libraries were sequenced with 50 bp single read sequencing on Illumina HiSeq 2500 machine at the University of Chicago Genomics Core as per the manufacturer’s protocols. RNA-seq reads were mapped to reference genome of Illumina iGenomes UCSC hg38 using TopHat^48^ version 2.1.0. Mapped reads were summarized for each gene using featureCounts^49^ version 1.5.0. Differential expression analysis was implemented using edgeR^50^ version 3.16.5. Only the genes of which counts per million is larger than one in at least two samples were kept and the library sizes across samples were normalized using the TMM method in the edgeR package. GSEA analysis of KEGG pathway was implemented using the clusterProfiler^47^ package.

### Primer sequences for ChIP-qPCR and RT-qPCR

ChIP experiments for histone K_bz_ and K_ac_ were carried out according to the instructions from the ChIP-IT® High Sensitivity Kit (Active Motif, Carlsbad, CA). ChIP products and input were analyzed by qPCR using SYBR® Select Master Mix (Life Technologies, Thermo Fisher Scientific Inc., Waltham, MA) and StepOnePlus Real-Time PCR system (Applied Biosystems, Thermo Fisher Scientific Inc., Waltham, MA). The sequences for ChIP-qPCR primers with forward followed by reverse are: *PLA2G4C* (GTGTTTCCTCCTGGTCCTGA/CTGAAAAAGCTGGAGCAACC), *ACHE* (GGGATCGCTAGTGGAAATGA/GTCCTGCCTTCTCAGGTGTC), *PLA2G15* (GCAGAGTTACAGGGGCTGAC/AGGAGCTGACCAGGACCTTT), and *PTGS2* (TCCCTCCTCTCCCCTTAAAA/CTGGGTTTCCGATTTTCTCA).

Total RNAs were extracted using the RNeasy Mini Kit (QIAGEN Inc, Valencia, CA). cDNA was prepared using RevertAid First Strand cDNA Synthesis Kit (Thermo Fisher Scientific Inc., Waltham, MA) following the manufacture’s protocol and analyzed by qPCR using SYBR® Select Master Mix (Life Technologies, Thermo Fisher Scientific Inc., Waltham, MA) and StepOnePlus Real-Time PCR system (Applied Biosystems, Thermo Fisher Scientific Inc., Waltham, MA). The sequences for RT-qPCR primer pairs with forward followed by reverse are: *PLA2G4C* (TGGGCAATATCTTCTCTCTAC/GGTAAATCGATGTTTCAGGTC), *ACHE* (CTGTGGTAGATGGAGACTTC/CCCCGTAAACCAGAAAATAC), *PLA2G15* (GAAAGCTACTTCACAATCTGG/CAGCCTGATATTGTCAATCC), *PTGS2* (AAGCAGGCTAATACTGATAGG/TGTTGAAAAGTAGTTCTGGG), and *ACTIN* (ACCTTCTACAATGAGCTGCG/CTGGATGGCTACGTACATGG).

### Data availability

The mass spectrometric data have been deposited to the ProteomeXchange Consortium with the dataset identifier PXD010332. The sequencing datasets reported in this paper have been submitted to the Gene Expression Omnibus database and are available under accession no. GSE108470.

## Electronic supplementary material

Supplementary Information

Peer Review File

Description of Additional Supplementary Files

Supplementary Data 1

Supplementary Data 2

## References

[1] Sabari BR, Zhang D, Allis CD, Zhao Y (2017). Metabolic regulation of gene expression through histone acylations. Nat. Rev. Mol. Cell Biol..

[2] Muller MM, Muir TW (2015). Histones: at the crossroads of peptide and protein chemistry. Chem. Rev..

[3] Xie ZY (2016). Metabolic regulation of gene expression by histone lysine beta-hydroxybutyrylation. Mol. Cell.

[4] Moellering RE, Cravatt BF (2013). Functional lysine modification by an intrinsically reactive primary glycolytic metabolite. Science.

[5] Simithy, J., Sidoli, S. & Garcia, B. A. Integrating proteomics and targeted metabolomics to understand global changes in histone modifications. *Proteomics* e1700309 (2018). 10.1002/pmic.201700309 PMC6261489 29512899

[6] Choudhary C, Weinert BT, Nishida Y, Verdin E, Mann M (2014). The growing landscape of lysine acetylation links metabolism and cell signalling. Nat. Rev. Mol. Cell Biol..

[7] Wellen KE (2009). ATP-citrate lyase links cellular metabolism to histone acetylation. Science.

[8] Takahashi H, McCaffery JM, Irizarry RA, Boeke JD (2006). Nucleocytosolic acetyl-coenzyme A synthetase is required for histone acetylation and global transcription. Mol. Cell.

[9] Sabari BR (2015). Intracellular crotonyl-CoA stimulates transcription through p300-catalyzed histone crotonylation. Mol. Cell.

[10] Harwood CS, Burchhardt G, Herrmann H, Fuchs G (1998). Anaerobic metabolism of aromatic compounds via the benzoyl-CoA pathway. FEMS Microbiol. Rev..

[11] Brusilow SW (1984). Treatment of episodic hyperammonemia in children with inborn errors of urea synthesis. N. Engl. J. Med..

[12] Yilmaz S, Unal F, Yuzbasioglu D (2009). The in vitro genotoxicity of benzoic acid in human peripheral blood lymphocytes. Cytotechnology.

[13] Pongsavee M (2015). Effect of sodium benzoate preservative on micronucleus induction, chromosome break, and Ala40Thr superoxide dismutase gene mutation in lymphocytes. Biomed. Res. Int..

[14] Park HW, Park EH, Yun HM, Rhim H (2011). Sodium benzoate-mediated cytotoxicity in mammalian cells. J. Food Biochem.

[15] Praphanproj V, Boyadjiev SA, Waber LJ, Brusilow SW, Geraghty MT (2000). Three cases of intravenous sodium benzoate and sodium phenylacetate toxicity occurring in the treatment of acute hyperammonaemia. J. Inherit. Metab. Dis..

[16] Chen Y, Chen W, Cobb MH, Zhao Y (2009). PTMap--a sequence alignment software for unrestricted, accurate, and full-spectrum identification of post-translational modification sites. Proc. Natl. Acad. Sci. USA.

[17] Huang H, Lin S, Garcia BA, Zhao Y (2015). Quantitative proteomic analysis of histone modifications. Chem. Rev..

[18] Peng C (2011). The first identification of lysine malonylation substrates and its regulatory enzyme. Mol. Cell. Proteomics.

[19] Zhang Z (2011). Identification of lysine succinylation as a new post-translational modification. Nat. Chem. Biol..

[20] De Ruijter AJM, Van Gennip AH, Caron HN, Kemp S, Van Kuilenburg ABP (2003). Histone deacetylases (HDACs): characterization of the classical HDAC family. Biochem. J..

[21] Du JT (2011). Sirt5 Is a NAD-dependent protein lysine demalonylase and desuccinylase. Science.

[22] Tan MJ (2014). Lysine glutarylation is a protein posttranslational modification regulated by SIRT5. Cell. Metab..

[23] Vaquero A (2006). SirT2 is a histone deacetylase with preference for histone H4 Lys 16 during mitosis. Genes Dev..

[24] Teng YB (2015). Efficient demyristoylase activity of SIRT2 revealed by kinetic and structural studies. Sci. Rep..

[25] Huang H, Sabari BR, Garcia BA, Allis CD, Zhao Y (2014). SnapShot: histone modifications. Cell.

[26] Yun M, Wu J, Workman JL, Li B (2011). Readers of histone modifications. Cell Res..

[27] McClure JJ (2017). Comparison of the deacylase and deacetylase activity of zinc-dependent HDACs. Acs. Chem. Biol..

[28] Jing H, Lin H (2015). Sirtuins in epigenetic regulation. Chem. Rev..

[29] Verreault A, Kaufman PD, Kobayashi R, Stillman B (1998). Nucleosomal DNA regulates the core-histone-binding subunit of the human Hat1 acetyltransferase. Curr. Biol..

[30] Parthun MR (2007). Hat1: the emerging cellular roles of a type B histone acetyltransferase. Oncogene.

[31] Vaquero A, Sternglanz R, Reinberg D (2007). NAD+-dependent deacetylation of H4 lysine 16 by class III HDACs. Oncogene.

[32] Goudarzi A (2016). Dynamic competing histone H4 K5K8 acetylation and butyrylation are hallmarks of highly active gene promoters. Mol. Cell.

[33] Kubota K, Ishizaki T (1991). Dose-dependent pharmacokinetics of benzoic acid following oral administration of sodium benzoate to humans. Eur. J. Clin. Pharmacol..

[34] Wang Y (2017). KAT2A coupled with the alpha-KGDH complex acts as a histone H3 succinyltransferase. Nature.

[35] Kim HS (2011). SIRT2 maintains genome integrity and suppresses tumorigenesis through regulating APC/C activity. Cancer Cell..

[36] Pettersen EF (2004). UCSF Chimera--a visualization system for exploratory research and analysis. J. Comput. Chem..

[37] Morris GM (2009). AutoDock4 and AutoDockTools4: automated docking with selective receptor flexibility. J. Comput. Chem..

[38] Trott O, Olson AJ (2010). AutoDock Vina: improving the speed and accuracy of docking with a new scoring function, efficient optimization, and multithreading. J. Comput. Chem..

[39] Liu X (2015). High-resolution metabolomics with Acyl-CoA profiling reveals widespread remodeling in response to diet. Mol. Cell. Proteomics.

[40] Delaval K (2007). Differential histone modifications mark mouse imprinting control regions during spermatogenesis. EMBO J..

[41] Langmead B, Salzberg SL (2012). Fast gapped-read alignment with Bowtie 2. Nat. Methods.

[42] Li H (2009). The Sequence Alignment/Map format and SAMtools. Bioinformatics.

[43] Shen L, Shao N, Liu X, Nestler E (2014). ngs.plot: Quick mining and visualization of next-generation sequencing data by integrating genomic databases. BMC Genomics.

[44] Zhang Y (2008). Model-based analysis of ChIP-Seq (MACS). Genome Biol..

[45] Robinson JT (2011). Integrative genomics viewer. Nat. Biotechnol..

[46] Stark, R. & Brown, G. DiffBind: differential binding analysis of ChIP-Seq peak data. *Bioconductor* https://bioconductor.org/packages/release/bioc/html/DiffBind.html (2011).

[47] Yu G, Wang LG, Han Y, He QY (2012). clusterProfiler: an R package for comparing biological themes among gene clusters. OMICS.

[48] Kim D (2013). TopHat2: accurate alignment of transcriptomes in the presence of insertions, deletions and gene fusions. Genome Biol..

[49] Liao Y, Smyth GK, Shi W (2014). featureCounts: an efficient general purpose program for assigning sequence reads to genomic features. Bioinformatics.

[50] Robinson MD, McCarthy DJ, Smyth GK (2010). edgeR: a Bioconductor package for differential expression analysis of digital gene expression data. Bioinformatics.

