# Supplementary material for: Lysine benzoylation is a histone mark regulated by SIRT2
Source: Nat Commun. 2018 Aug 28;9:3374. doi: 10.1038/s41467-018-05567-w (PMC6113264; doi:10.1038/s41467-018-05567-w)
Supplement: Supplementary file 1 — Supplementary Information [file 41467_2018_5567_MOESM1_ESM.pdf]

Lysine benzoylation is a histone mark regulated by SIRT2

Huang et al.

Table of Contents

Supplementary Table 1………………………………………………………Page 3

Supplementary Table 2………………………………………………………Page 4

Supplementary Table 3………………………………………………………Page 5

Supplementary Figure 1……………………………………………………Page 6

Supplementary Figure 2………………………………………………Pages 7-29

Supplementary Figure 3…………………………………………………..Page 30

Supplementary Figure 4……………………………………………Pages 31-46

Supplementary Figure 5…………………………………………………..Page 47

Supplementary Figure 6…………………………………………………..Page 48

Supplementary Figure 7…………………………………………………..Page 49

Supplementary Figure 8…………………………………………………..Page 50

Supplementary Figure 9…………………………………………………..Page 51

Supplementary Table 1. Histone K_{bz} sites identified in HepG2 cells

K_{bz} Site	Modified Peptide	Mascot Score
H3K4bz	$TK_{bz}QTAR$	26
H3K9bz	$K_{bz}STGGK_{ac}APR$	55
H3K14bz	$K_{pr}STGGK_{bz}APR$	38
H3K18bz	$K_{bz}QLATK_{ac}AAR$	48
H3K23bz	$K_{ac}QLATK_{bz}AAR$	57
H3K27bz	$K_{bz}SAPATGGVK$	24
H4K5bz	$GK_{bz}GGK_{ac}GLGK$<br>$GK_{bz}GGK_{pr}GLGK_{ac}GGAK_{ac}R$	30<br>106
H4K8bz	$GK_{ac}GGK_{bz}GLGK_{ac}GGAK$<br>$GK_{pr}GGK_{bz}GLGK_{pr}GGAK_{ac}R$	79<br>83
H4K12bz	$GGK_{ac}GLGK_{bz}GGAK$<br>$GK_{ac}GGK_{pr}GLGK_{bz}GGAK_{ac}R$	40<br>85
H4K16bz	$GLGK_{ac}GGAK_{bz}R$	23
H2AK9bz	$GK_{ac}QGGK_{bz}AR$	35
H2AK13bz	$AK_{bz}AK_{pr}TR$	34
H2AK36bz	$K_{bz}GNYAER$	50
H2BK5bz	$PEPTK_{bz}SAPAPK$	70
H2BK11bz	$SAPAPK_{bz}K$	24
H2BK16bz	$K_{bz}AVTK_{ac}AQK$	39
H2BK20bz	$K_{ac}AVTK_{bz}AQK$	34
H1K33bz	$K_{bz}SAGAAK_{pr}R$	37
H1K104bz	$GTGASGSFK_{bz}LNK$	53
H1K147bz	$ATGAATPK_{bz}K$	33
H1K190bz	$SASK_{bz}AVKPK$	30

Supplementary Table 2. D_5-labeled histone K_{bz} sites identified from HepG2 cells that were treated with 5 mM D_5-Sodium Benzoate for 24 hours.

Kbz Site	Modified Peptide	Mascot Score
H3K4bz	$TK_{bz}QTAR$	33
H3K9bz	$K_{bz}STGGK_{ac}APR$	41
H3K14bz	$STGGK_{bz}APR$	50
H3K18bz	$K_{bz}QLATK_{ac}AAR$	58
H3K23bz	$KQLATK_{bz}AAR$	53
H4K5bz	$GK_{bz}GGK_{ac}GLGK_{ac}GGAK_{ac}R$	86
H4K8bz	$GK_{ac}GGK_{bz}GLGK_{ac}GGAK_{ac}R$	70
H4K12bz	$GLGK_{bz}GGAK_{ac}R$<br>$GK_{ac}GGK_{ac}GLGK_{bz}GGAK_{ac}R$	51<br>92
H4K16bz	$GGK_{pr}GLGK_{pr}GGAK_{bz}R$	28
H2AK9bz	$GK_{ac}QGGK_{bz}AR$	34
H2AK13bz	$AK_{bz}AK_{pr}SR$	24
H2BK5bz	$PEPTK_{bz}SAPAPK$	57
H2BK11bz	$SAPAPK_{bz}K$	35
H2BK16bz	$K_{bz}AVTK_{ac}AQK$	47
H2BK20bz	$KAVTK_{bz}AQK$	45

Supplementary Table 3. Comparison of the size and hydrophobicity changes caused by lysine acylations. Their size and hydrophobicity are presented by the molecule volumes and LogP of different acyl groups. All the values were calculated using the robustly trained fragment-based algorithm of the Molinspiration Property Calculation Service (www.molinspiration.com).

**Acylation**	**Molecule volume (Å^3)**	**LogP**
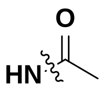<br>Acetylation	48.18	0.51
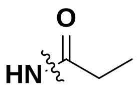<br>Propionylation	64.98	1.01
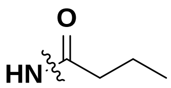<br>Butyrylation	81.78	1.57
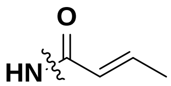<br>Crotonylation	75.60	1.03
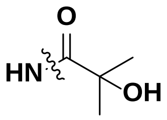<br>2-Hydroxyisobutyrylation	89.26	0.31
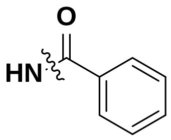<br>Benzoylation	103.03	1.73

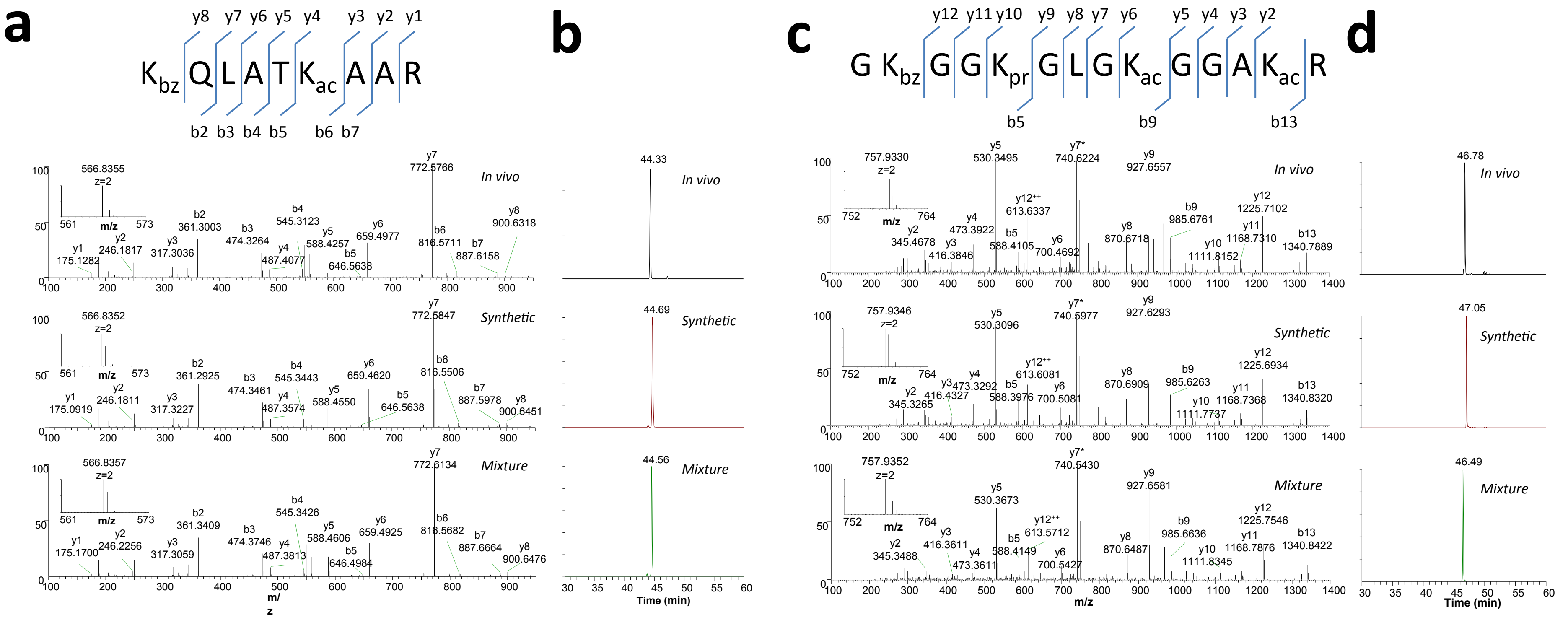

Supplementary Figure 1. Verification of histone K_{bz}.
(a) The MS/MS spectra of an *in vivo* peptide bearing a PTM ($K_{+104.0268}QLATK_{ac}AAR$) (top), a synthetic lysine benzoylated peptide corresponding to the sequence of the *in vivo* peptide (middle) and a mixture of the two peptides (bottom).
(b) Extracted ion chromatograms of the *in vivo*-derived peptide ($K_{+104.0268}QLATK_{ac}AAR$) (top), the synthetic K_{bz} counterpart (middle), and their mixture (bottom) by HPLC-MS/MS analysis.
(c) The MS/MS spectra of an *in vivo* peptide bearing a PTM ($GK_{+104.0268}GGK_{pr}GLGK_{ac}GGAK_{ac}R$) (top), a synthetic lysine benzoylated peptide corresponding to the sequence of the *in vivo* peptide (middle) and a mixture of the two peptides (bottom).
(d) Extracted ion chromatograms of the *in vivo*-derived peptide ($GK_{+104.0268}GGK_{pr}GLGK_{ac}GGAK_{ac}R$) (top), the synthetic K_{bz} counterpart (middle), and their mixture (bottom) by HPLC-MS/MS analysis.

MS/MS Fragmentation of **TKQTAR**
Found in **Q71DI3**, Histone H3.2
Match to Query 744: 807.423654 from(404.719103,2+)

H3K4bz

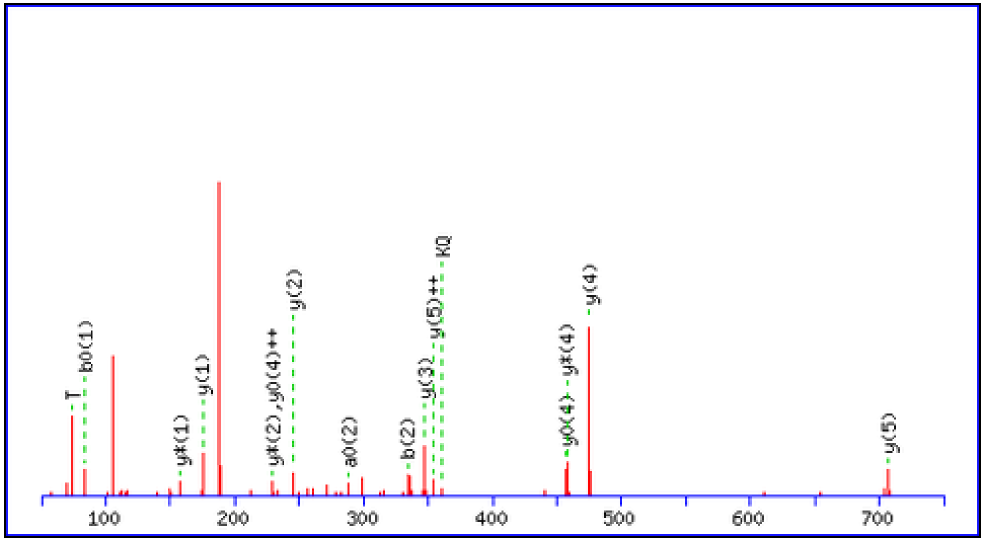

Monoisotopic mass of neutral peptide Mr(calc): 807.42
Variable modifications:
K2 : Benzoyl (K)
Ions Score: 26 **Expect:** 0.93
Matches (Bold Red): 18/100 fragment ions using 30 most intense peaks

#	Immon.	a	a^{++}	a^{*}	a^{*++}	a^{0}	a^{0++}	b	b^{++}	b^{*}	b^{*++}	b^{0}	b^{0++}	Seq.	y	y^{++}	y^{*}	y^{*++}	y^{0}	y^{0++}	#
1	**74.06**	**74.06**	37.53			56.05	28.53	102.05	51.53			**84.04**	42.53	T							6
2	205.13	306.18	153.59	289.15	145.08	**288.17**	144.59	**334.18**	167.59	317.15	159.08	316.17	158.59	K	**707.38**	**354.20**	690.36	345.68	689.37	345.19	5
3	101.07	434.24	217.62	417.21	209.11	416.23	208.62	462.23	231.62	445.21	223.11	444.22	222.62	Q	**475.26**	238.13	**458.24**	229.62	**457.25**	**229.13**	4
4	**74.06**	535.29	268.15	518.26	259.63	517.28	259.14	563.28	282.14	546.26	273.63	545.27	273.14	T	**347.20**	174.11	330.18	165.59	329.19	165.10	3
5	44.05	606.32	303.67	589.30	295.15	588.31	294.66	634.32	317.66	617.29	309.15	616.31	308.66	A	**246.16**	123.58	**229.13**	115.07			2
6	129.11													R	**175.12**	88.06	**158.09**	79.55			1

MS/MS Fragmentation of **KSTGGKAPR**

Found in **Q71DI3**, Histone H3.2

Match to Query 2961: 1046.550752 from(524.282652,2+)

H3K9bz

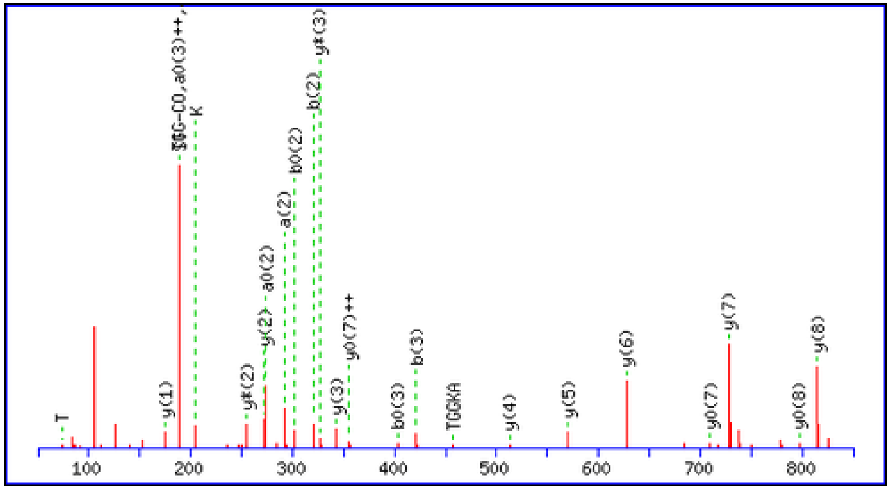

Monoisotopic mass of neutral peptide Mr(calc): 1046.55
Variable modifications:
K1 : Benzoyl (K)
K6 : Acetyl (K)
Ions Score: 55 **Expect:** 0.0022
Matches (Bold Red): 27/179 fragment ions using 39 most intense peaks

#	Immon.	a	a^{++}	a^{*}	a^{*++}	a^{0}	a^{0++}	b	b^{++}	b^{*}	b^{*++}	b^{0}	b^{0++}	Seq.	y	y^{++}	y^{*}	y^{*++}	y^{0}	y^{0++}	#
1	**205.13**	**205.13**	103.07	**188.11**	94.56			233.13	117.07	216.10	108.55			K							9
2	60.04	**292.17**	146.59	275.14	138.07	**274.16**	137.58	**320.16**	160.58	303.13	152.07	**302.15**	151.58	S	**815.44**	408.22	798.41	399.71	**797.43**	399.22	8
3	**74.06**	393.21	197.11	376.19	188.60	375.20	**188.10**	**421.21**	211.11	404.18	202.59	**403.20**	202.10	T	**728.40**	364.71	711.38	356.19	**710.39**	**355.70**	7
4	30.03	450.23	225.62	433.21	217.11	432.22	216.62	478.23	239.62	461.20	231.11	460.22	230.61	G	**627.36**	314.18	610.33	305.67			6
5	30.03	507.26	254.13	490.23	245.62	489.25	245.13	535.25	268.13	518.22	259.62	517.24	259.12	G	**570.34**	285.67	553.31	277.16			5
6	143.12	677.36	339.18	660.34	330.67	659.35	330.18	705.36	353.18	688.33	344.67	687.35	344.18	K	**513.31**	257.16	496.29	248.65			4
7	44.05	748.40	374.70	731.37	366.19	730.39	365.70	776.39	388.70	759.37	380.19	758.38	379.70	A	**343.21**	172.11	**326.18**	163.59			3
8	70.07	845.45	423.23	828.43	414.72	827.44	414.22	873.45	437.23	856.42	428.71	855.44	428.22	P	**272.17**	136.59	**255.15**	128.08			2
9	129.11													R	**175.12**	88.06	158.09	79.55			1

MS/MS Fragmentation of **KSTGGKAPR**

Found in **Q71DI3**, Histone H3.2

Match to Query 2348: 1060.566124 from(531.290338,2+)

H3K14bz

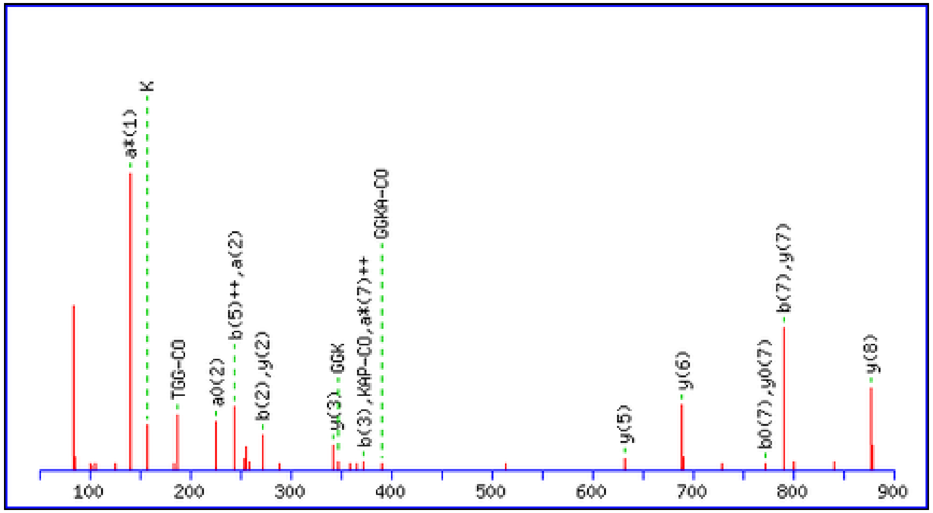

Monoisotopic mass of neutral peptide Mr(calc): 1060.57

Variable modifications:

K1 : Propionyl (K)

K6 : Benzoyl (K)

Ions Score: 38 **Expect:** 0.13

Matches (Bold Red): 22/178 fragment ions using 23 most intense peaks

#	Immon.	a	a^{++}	a^{*}	a^{*++}	a^{0}	a^{0++}	b	b^{++}	b^{*}	b^{*++}	b^{0}	b^{0++}	Seq.	y	y^{++}	y^{*}	y^{*++}	y^{0}	y^{0++}	#
1	**157.13**	**157.13**	79.07	**140.11**	70.56			185.13	93.07	168.10	84.55			K							9
2	60.04	**244.17**	122.59	227.14	114.07	**226.16**	113.58	**272.16**	136.58	255.13	128.07	254.15	127.58	S	**877.45**	439.23	860.43	430.72	859.44	430.22	8
3	74.06	345.21	173.11	328.19	164.60	327.20	164.10	**373.21**	187.11	356.18	178.59	355.20	178.10	T	**790.42**	395.71	773.39	387.20	**772.41**	386.71	7
4	30.03	402.23	201.62	385.21	193.11	384.22	192.62	430.23	215.62	413.20	207.11	412.22	206.61	G	**689.37**	345.19	672.35	336.68			6
5	30.03	459.26	230.13	442.23	221.62	441.25	221.13	487.25	**244.13**	470.22	235.62	469.24	235.12	G	**632.35**	316.68	615.32	308.17			5
6	205.13	691.38	346.19	674.35	337.68	673.37	337.19	719.37	360.19	702.35	351.68	701.36	351.18	K	575.33	288.17	558.30	279.66			4
7	44.05	762.41	381.71	745.39	**373.20**	744.40	372.71	**790.41**	395.71	773.38	387.20	**772.40**	386.70	A	**343.21**	172.11	326.18	163.59			3
8	70.07	859.47	430.24	842.44	421.72	841.46	421.23	887.46	444.23	870.44	435.72	869.45	435.23	P	**272.17**	136.59	255.15	128.08			2
9	129.11													R	175.12	88.06	158.09	79.55			1

MS/MS Fragmentation of **KQLATKAAR**
Found in **Q71DI3**, Histone H3.2
Match to Query 3605: 1131.639834 from(566.827193,2+)

H3K18bz

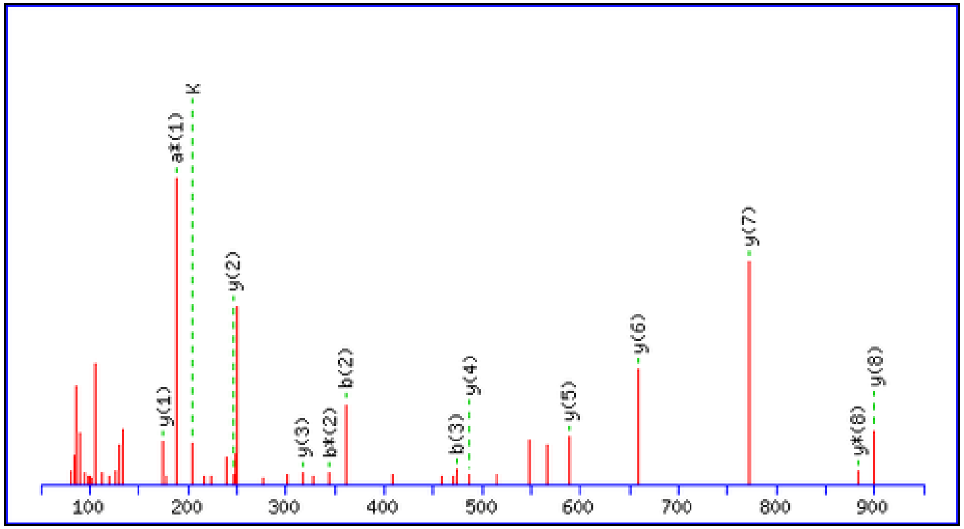

Monoisotopic mass of neutral peptide Mr(calc): 1131.64
Variable modifications:
K1 : Benzoyl (K)
K6 : Acetyl (K)
Ions Score: 48 **Expect:** 0.019
Matches (Bold Red): 15/170 fragment ions using 33 most intense peaks

#	Immon.	a	a^{++}	a^{*}	a^{*++}	a^{0}	a^{0++}	b	b^{++}	b^{*}	b^{*++}	b^{0}	b^{0++}	Seq.	y	y^{++}	y^{*}	y^{*++}	y^{0}	y^{0++}	#
1	**205.13**	**205.13**	103.07	**188.11**	94.56			233.13	117.07	216.10	108.55			K							9
2	101.07	333.19	167.10	316.17	158.59			**361.19**	181.10	**344.16**	172.58			Q	**900.53**	450.77	**883.50**	442.25	882.52	441.76	8
3	86.10	446.28	223.64	429.25	215.13			**474.27**	237.64	457.24	229.13			L	**772.47**	386.74	755.44	378.22	754.46	377.73	7
4	44.05	517.31	259.16	500.29	250.65			545.31	273.16	528.28	264.64			A	**659.38**	330.20	642.36	321.68	641.37	321.19	6
5	74.06	618.36	309.68	601.33	301.17	600.35	300.68	646.36	323.68	629.33	315.17	628.35	314.68	T	**588.35**	294.68	571.32	286.16	570.34	285.67	5
6	143.12	788.47	394.74	771.44	386.22	770.46	385.73	816.46	408.73	799.43	400.22	798.45	399.73	K	**487.30**	244.15	470.27	235.64			4
7	44.05	859.50	430.26	842.48	421.74	841.49	421.25	887.50	444.25	870.47	435.74	869.49	435.25	A	**317.19**	159.10	300.17	150.59			3
8	44.05	930.54	465.77	913.51	457.26	912.53	456.77	958.54	479.77	941.51	471.26	940.53	470.77	A	**246.16**	123.58	229.13	115.07			2
9	129.11													R	**175.12**	88.06	158.09	79.55			1

MS/MS Fragmentation of **KQLATKAAR**
Found in **Q71DI3**, Histone H3.2
Match to Query 2786: 1131.640066 from(566.827309,2+)

H3K23bz

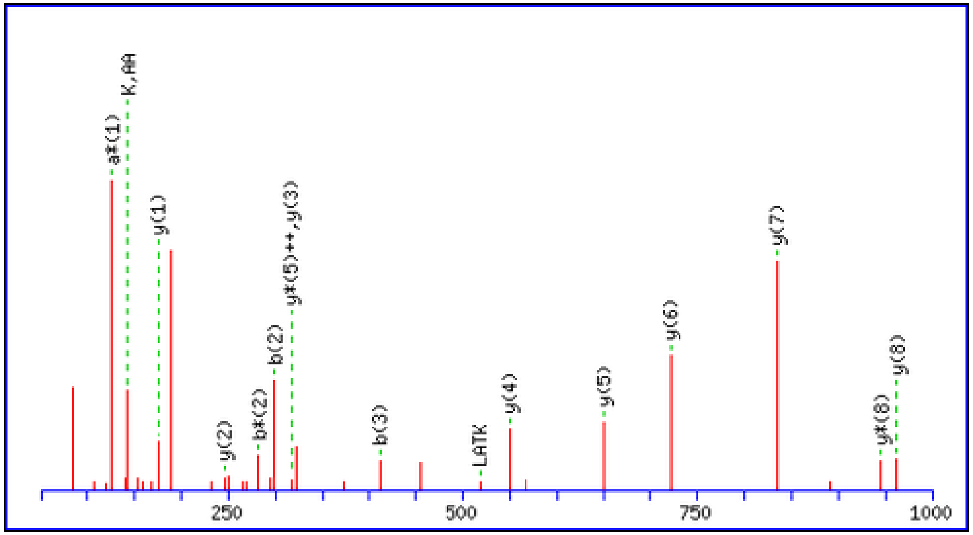

Monoisotopic mass of neutral peptide Mr(calc): 1131.64
Variable modifications:
K1 : Acetyl (K)
K6 : Benzoyl (K)
Ions Score: 57 **Expect:** 0.0033
Matches (Bold Red): 18/168 fragment ions using 26 most intense peaks

#	Immon.	a	a++	a*	a*++	a0	a0++	b	b++	b*	b*++	b0	b0++	Seq.	y	y++	y*	y*++	y0	y0++	#
1	**143.12**	**143.12**	72.06	**126.09**	63.55			171.11	86.06	154.09	77.55			K							9
2	101.07	271.18	136.09	254.15	127.58			**299.17**	150.09	**282.14**	141.58			Q	**962.54**	481.77	**945.52**	473.26	944.53	472.77	8
3	86.10	384.26	192.63	367.23	184.12			**412.26**	206.63	395.23	198.12			L	**834.48**	417.75	817.46	409.23	816.47	408.74	7
4	44.05	455.30	228.15	438.27	219.64			483.29	242.15	466.27	233.64			A	**721.40**	361.20	704.37	352.69	703.39	352.20	6
5	74.06	556.35	278.68	539.32	270.16	538.33	269.67	584.34	292.67	567.31	284.16	566.33	283.67	T	**650.36**	325.68	633.34	**317.17**	632.35	316.68	5
6	205.13	788.47	394.74	771.44	386.22	770.46	385.73	816.46	408.73	799.43	400.22	798.45	399.73	K	**549.31**	275.16	532.29	266.65			4
7	44.05	859.50	430.26	842.48	421.74	841.49	421.25	887.50	444.25	870.47	435.74	869.49	435.25	A	**317.19**	159.10	300.17	150.59			3
8	44.05	930.54	465.77	913.51	457.26	912.53	456.77	958.54	479.77	941.51	471.26	940.53	470.77	A	**246.16**	123.58	229.13	115.07			2
9	129.11													R	**175.12**	88.06	158.09	79.55			1

MS/MS Fragmentation of **KSAPATGGVK**
Found in **P68433**, Histone H3.1
Match to Query 4276: 1018.545874 from(510.280213,2+)

H3K27bz

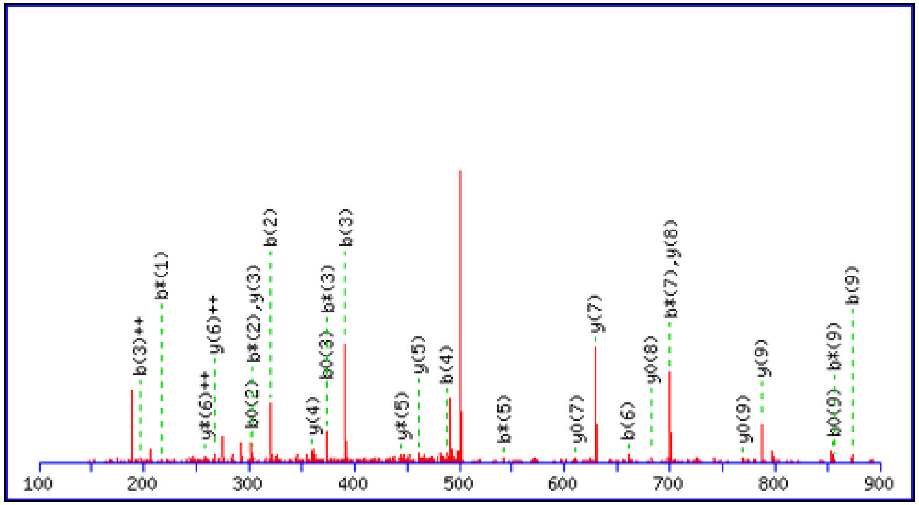

Monoisotopic mass of neutral peptide Mr(calc): 1018.54
Variable modifications:
K1 : Benzoyl (K)
Ions Score: 24 **Expect:** 3.6
Matches (Bold Red): 27/98 fragment ions using 75 most intense peaks

#	b	b^{++}	b^{*}	b^{*++}	b^{0}	b^{0++}	Seq.	y	y^{++}	y^{*}	y^{*++}	y^{0}	y^{0++}	#
1	233.13	117.07	**216.10**	108.55			K							10
2	**320.16**	160.58	**303.13**	152.07	**302.15**	151.58	S	**787.43**	394.22	770.40	385.71	**769.42**	385.21	9
3	**391.20**	**196.10**	**374.17**	187.59	**373.19**	187.10	A	**700.40**	350.70	683.37	342.19	**682.39**	341.70	8
4	**488.25**	244.63	471.22	236.12	470.24	235.62	P	**629.36**	315.18	612.34	306.67	**611.35**	306.18	7
5	559.29	280.15	**542.26**	271.63	541.28	271.14	A	532.31	**266.66**	515.28	**258.14**	514.30	257.65	6
6	**660.34**	330.67	643.31	322.16	642.32	321.67	T	**461.27**	231.14	**444.25**	222.63	443.26	222.13	5
7	717.36	359.18	**700.33**	350.67	699.35	350.18	G	**360.22**	180.62	343.20	172.10			4
8	774.38	387.69	757.35	379.18	756.37	378.69	G	**303.20**	152.10	286.18	143.59			3
9	**873.45**	437.23	**856.42**	428.71	**855.44**	428.22	V	246.18	123.59	229.15	115.08			2
10							K	147.11	74.06	130.09	65.55			1

MS/MS Fragmentation of **GKGGKGLGK**

Found in **P62805**, Histone H4

Match to Query 2000: 946.523290 from(474.268921,2+)

H4K5bz

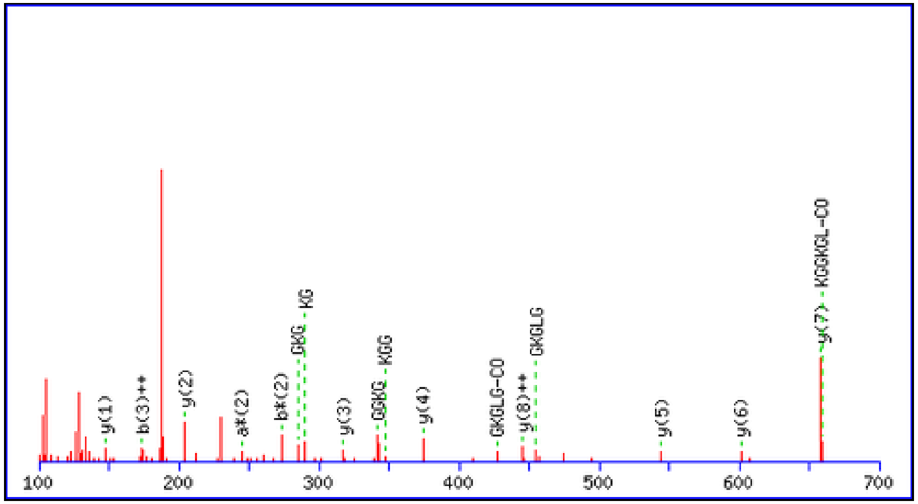

Monoisotopic mass of neutral peptide Mr(calc): 946.52

Variable modifications:

K2 : Benzoyl (K)

K5 : Acetyl (K)

Ions Score: 30 **Expect:** 0.71

Matches (Bold Red): 25/141 fragment ions using 44 most intense peaks

#	Immon.	a	a++	a*	a*++	b	b++	b*	b*++	Seq.	y	y++	y*	y*++	#
1	30.03	30.03	15.52			58.03	29.52			G					9
2	205.13	262.15	131.58	**245.13**	123.07	**290.15**	145.58	**273.12**	137.07	K	890.51	**445.76**	873.48	437.25	8
3	30.03	319.18	160.09	302.15	151.58	**347.17**	**174.09**	330.14	165.58	G	**658.39**	329.70	641.36	321.18	7
4	30.03	376.20	188.60	359.17	180.09	404.19	202.60	387.17	194.09	G	**601.37**	301.19	584.34	292.67	6
5	143.12	546.30	273.66	529.28	265.14	574.30	287.65	557.27	279.14	K	**544.35**	272.68	527.32	264.16	5
6	30.03	603.32	302.17	586.30	293.65	631.32	316.16	614.29	307.65	G	**374.24**	187.62	357.21	179.11	4
7	**86.10**	716.41	358.71	699.38	350.19	744.40	372.71	727.38	364.19	L	**317.22**	159.11	300.19	150.60	3
8	30.03	773.43	387.22	756.40	378.71	801.43	401.22	784.40	392.70	G	**204.13**	102.57	187.11	94.06	2
9	101.11									K	**147.11**	74.06	130.09	65.55	1

MS/MS Fragmentation of **GKGGKGLGKGGAK**

Found in **P62805**, Histone H4

Match to Query 4805: 1301.707828 from(651.861190,2+)

H4K8bz

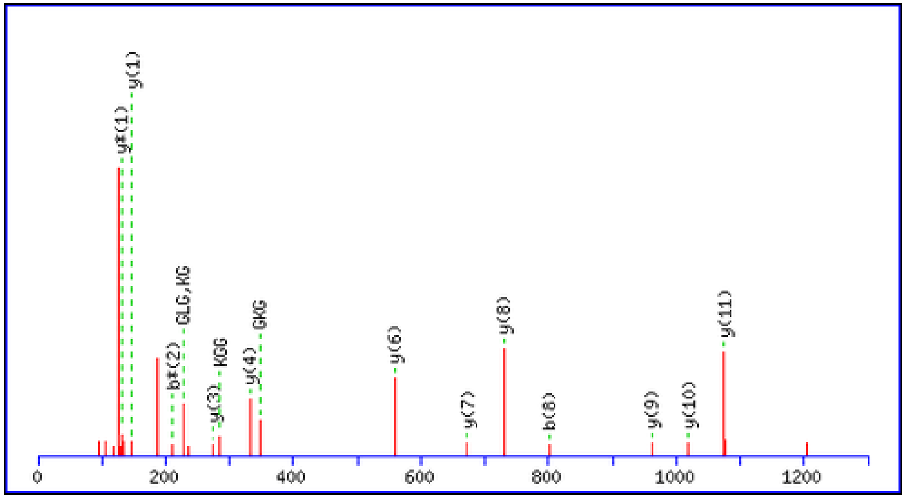

Monoisotopic mass of neutral peptide Mr(calc): 1301.71

Variable modifications:

K2 : Acetyl (K)

K5 : Benzoyl (K)

K9 : Acetyl (K)

Ions Score: 79 **Expect:** 1.8e-005

Matches (Bold Red): 23/235 fragment ions using 19 m

#	Immon.	a	a^{++}	a*	a^{*++}	b	b^{++}	b*	b^{*++}	Seq.	y	y^{++}	y*	y^{*++}	#
1	30.03	30.03	15.52			58.03	29.52			G					13
2	143.12	200.14	100.57	183.11	92.06	**228.13**	114.57	**211.11**	106.06	K	1245.69	623.35	1228.67	614.84	12
3	30.03	257.16	129.08	240.13	120.57	**285.16**	143.08	268.13	134.57	G	**1075.59**	538.30	1058.56	529.79	11
4	30.03	314.18	157.59	297.16	149.08	342.18	171.59	325.15	163.08	G	**1018.57**	509.79	1001.54	501.27	10
5	205.13	546.30	273.66	529.28	265.14	574.30	287.65	557.27	279.14	K	**961.55**	481.28	944.52	472.76	9
6	30.03	603.32	302.17	586.30	293.65	631.32	316.16	614.29	307.65	G	**729.43**	365.22	712.40	356.70	8
7	86.10	716.41	358.71	699.38	350.19	744.40	372.71	727.38	364.19	L	**672.40**	336.71	655.38	328.19	7
8	30.03	773.43	387.22	756.40	378.71	**801.43**	401.22	784.40	392.70	G	**559.32**	280.16	542.29	271.65	6
9	143.12	943.54	472.27	926.51	463.76	971.53	486.27	954.50	477.76	K	502.30	251.65	485.27	243.14	5
10	30.03	1000.56	500.78	983.53	492.27	1028.55	514.78	1011.53	506.27	G	**332.19**	166.60	315.17	158.09	4
11	30.03	1057.58	529.29	1040.55	520.78	1085.57	543.29	1068.55	534.78	G	**275.17**	138.09	258.14	129.58	3
12	44.05	1128.62	564.81	1111.59	556.30	1156.61	578.81	1139.58	570.30	A	218.15	109.58	201.12	101.07	2
13	101.11									K	**147.11**	74.06	**130.09**	65.55	1

MS/MS Fragmentation of **GGKGLGKGGAK**

Found in **P62805**, Histone H4

Match to Query 3188: 1074.582010 from(538.298281,2+)

H4K12bz

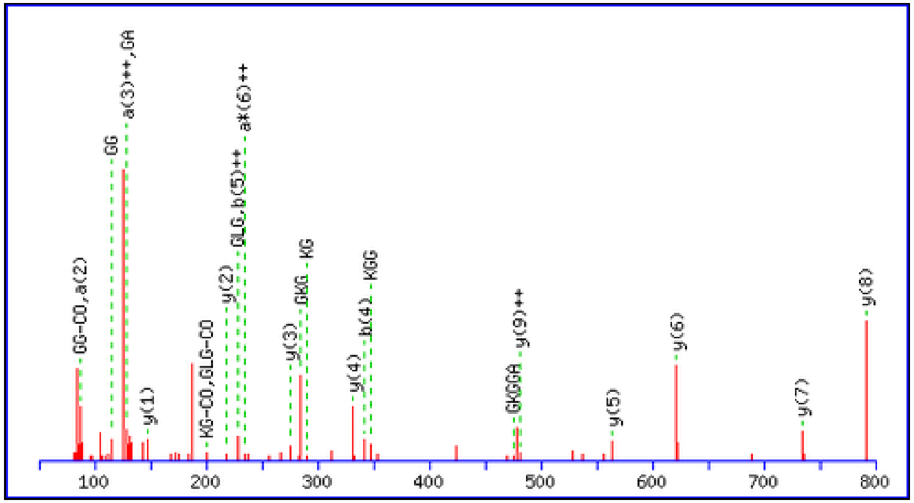

Monoisotopic mass of neutral peptide Mr(calc): 1074.58

Variable modifications:

K3 : Acetyl (K)

K7 : Benzoyl (K)

Ions Score: 40 **Expect:** 0.086

Matches (Bold Red): 31/185 fragment ions u

#	Immon.	a	a++	a*	a*++	b	b++	b*	b*++	Seq.	y	y++	y*	y*++	#
1	30.03	30.03	15.52			58.03	29.52			G					11
2	30.03	**87.06**	44.03			**115.05**	58.03			G	1018.57	509.79	1001.54	501.27	10
3	143.12	257.16	**129.08**	240.13	120.57	**285.16**	143.08	268.13	134.57	K	961.55	**481.28**	944.52	472.76	9
4	30.03	314.18	157.59	297.16	149.08	**342.18**	171.59	325.15	163.08	G	**791.44**	396.22	774.41	387.71	8
5	86.10	427.27	214.14	410.24	205.62	455.26	**228.13**	438.23	219.62	L	**734.42**	367.71	717.39	359.20	7
6	30.03	484.29	242.65	467.26	**234.13**	512.28	256.64	495.26	248.13	G	**621.34**	311.17	604.31	302.66	6
7	205.13	716.41	358.71	699.38	350.19	744.40	372.71	727.38	364.19	K	**564.31**	282.66	547.29	274.15	5
8	30.03	773.43	387.22	756.40	378.71	801.43	401.22	784.40	392.70	G	**332.19**	166.60	315.17	158.09	4
9	30.03	830.45	415.73	813.43	407.22	858.45	429.73	841.42	421.21	G	**275.17**	138.09	258.14	129.58	3
10	44.05	901.49	451.25	884.46	442.73	929.48	465.25	912.46	456.73	A	**218.15**	109.58	201.12	101.07	2
11	101.11									K	**147.11**	74.06	130.09	65.55	1

MS/MS Fragmentation of **GLGKGGAKR**

Found in **P62805**, Histone H4

Match to Query 2422: 988.542272 from(495.278412,2+)

H4K16bz

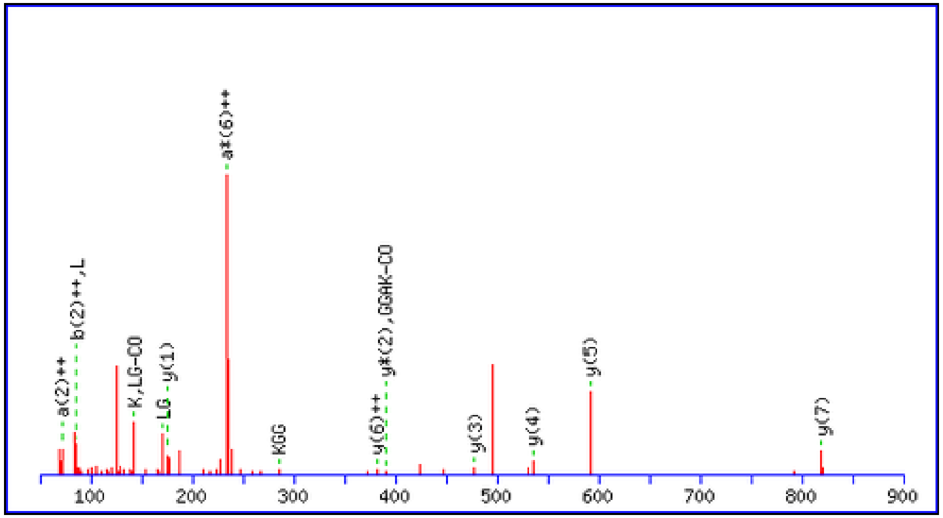

Monoisotopic mass of neutral peptide Mr(calc): 988.55
Variable modifications:
K4 : Acetyl (K)
K8 : Benzoyl (K)
Ions Score: 23 **Expect:** 4.1
Matches (Bold Red): 19/133 fragment ions using 31 most intense peaks

#	Immon.	a	a++	a*	a*++	b	b++	b*	b*++	Seq.	y	y++	y*	y*++	#
1	30.03	30.03	15.52			58.03	29.52			G					9
2	**86.10**	**143.12**	**72.06**			**171.11**	**86.06**			L	932.53	466.77	915.50	458.26	8
3	30.03	200.14	100.57			228.13	114.57			G	**819.45**	410.23	802.42	401.71	7
4	**143.12**	370.24	185.63	353.22	177.11	398.24	199.62	381.21	191.11	K	762.43	**381.72**	745.40	373.20	6
5	30.03	427.27	214.14	410.24	205.62	455.26	228.13	438.23	219.62	G	**592.32**	296.66	575.29	288.15	5
6	30.03	484.29	242.65	467.26	**234.13**	512.28	256.64	495.26	248.13	G	**535.30**	268.15	518.27	259.64	4
7	44.05	555.32	278.17	538.30	269.65	583.32	292.16	566.29	283.65	A	**478.28**	239.64	461.25	231.13	3
8	205.13	787.45	394.23	770.42	385.71	815.44	408.22	798.41	399.71	K	407.24	204.12	**390.21**	195.61	2
9	129.11									R	**175.12**	88.06	158.09	79.55	1

MS/MS Fragmentation of **GKQGGKAR**
Found in **Q6FI13**, Histone H2A type 2-A
Match to Query 1498: 946.498578 from(474.256565,2+)

H2AK9bz

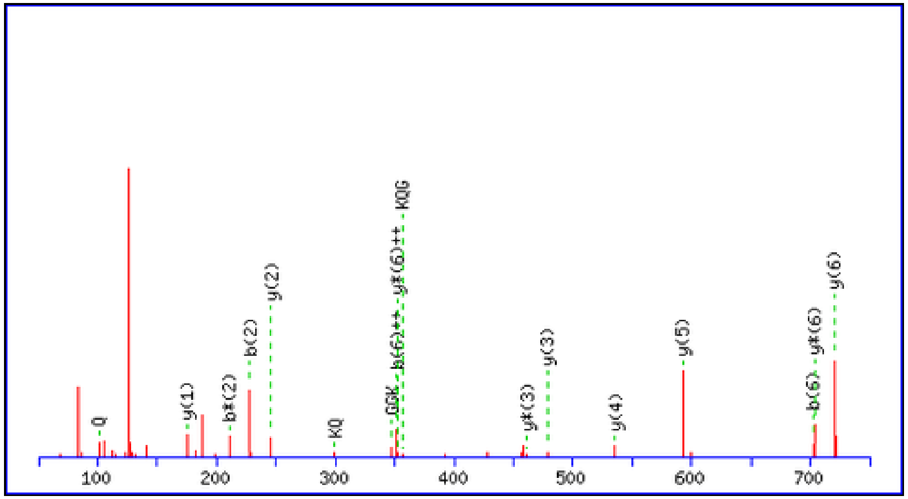

Monoisotopic mass of neutral peptide Mr(calc): 946.50
Variable modifications:
K2 : Acetyl (K)
K6 : Benzoyl (K)
Ions Score: 35 **Expect:** 0.2
Matches (Bold Red): 18/117 fragment ions using 30 most intense peaks

#	Immon.	a	a^{++}	a^*	a^{*++}	b	b^{++}	b^*	b^{*++}	Seq.	y	y^{++}	y^*	y^{*++}	#
1	30.03	30.03	15.52			58.03	29.52			G					8
2	143.12	200.14	100.57	183.11	92.06	**228.13**	114.57	**211.11**	106.06	K	890.48	445.75	873.46	437.23	7
3	**101.07**	328.20	164.60	311.17	156.09	**356.19**	178.60	339.17	170.09	Q	**720.38**	360.69	**703.35**	**352.18**	6
4	30.03	385.22	193.11	368.19	184.60	413.21	207.11	396.19	198.60	G	**592.32**	296.66	575.29	288.15	5
5	30.03	442.24	221.62	425.21	213.11	470.24	235.62	453.21	227.11	G	**535.30**	268.15	518.27	259.64	4
6	205.13	674.36	337.68	657.34	329.17	**702.36**	**351.68**	685.33	343.17	K	**478.28**	239.64	**461.25**	231.13	3
7	44.05	745.40	373.20	728.37	364.69	773.39	387.20	756.37	378.69	A	**246.16**	123.58	229.13	115.07	2
8	129.11									R	**175.12**	88.06	158.09	79.55	1

MS/MS Fragmentation of **AKAKTR**

Found in **P04908**, Histone H2A type 1-B/E

Match to Query 702: 833.476084 from(417.745318,2+)

H2AK13bz

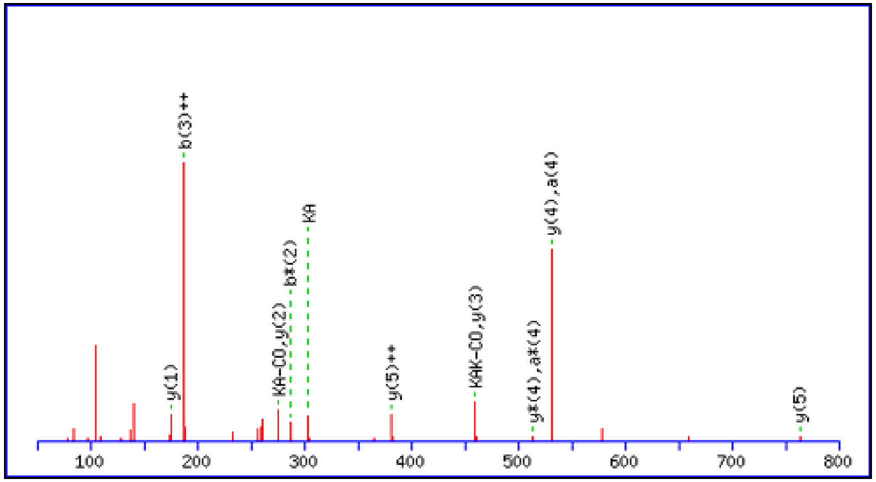

Monoisotopic mass of neutral peptide Mr(calc): 833.48
Variable modifications:
K2 : Benzoyl (K)
K4 : Propionyl (K)
Ions Score: 34 **Expect:** 0.16
Matches (Bold Red): 16/86 fragment ions using 18 most intense peaks

#	Immon.	a	a^{++}	a*	a^{*++}	a^{0}	a^{0++}	b	b^{++}	b*	b^{*++}	b^{0}	b^{0++}	Seq.	y	y^{++}	y*	y^{*++}	y^{0}	y^{0++}	#
1	44.05	44.05	22.53					72.04	36.53					A							6
2	205.13	**276.17**	138.59	259.14	130.08			**304.17**	152.59	**287.14**	144.07			K	**763.45**	**382.23**	746.42	373.71	745.44	373.22	5
3	44.05	347.21	174.11	330.18	165.59			375.20	**188.10**	358.18	179.59			A	**531.32**	266.17	**514.30**	257.65	513.31	257.16	4
4	157.13	**531.33**	266.17	**514.30**	257.65			559.32	280.17	542.30	271.65			K	**460.29**	230.65	443.26	222.13	442.28	221.64	3
5	74.06	632.38	316.69	615.35	308.18	614.37	307.69	660.37	330.69	643.34	322.18	642.36	321.68	T	**276.17**	138.59	259.14	130.07	258.16	129.58	2
6	129.11													R	**175.12**	88.06	158.09	79.55			1

MS/MS Fragmentation of **KGNYAER**
Found in **Q6FI13**, Histone H2A type 2-A
Match to Query 1446: 940.444116 from(471.229334,2+)

H2AK36bz

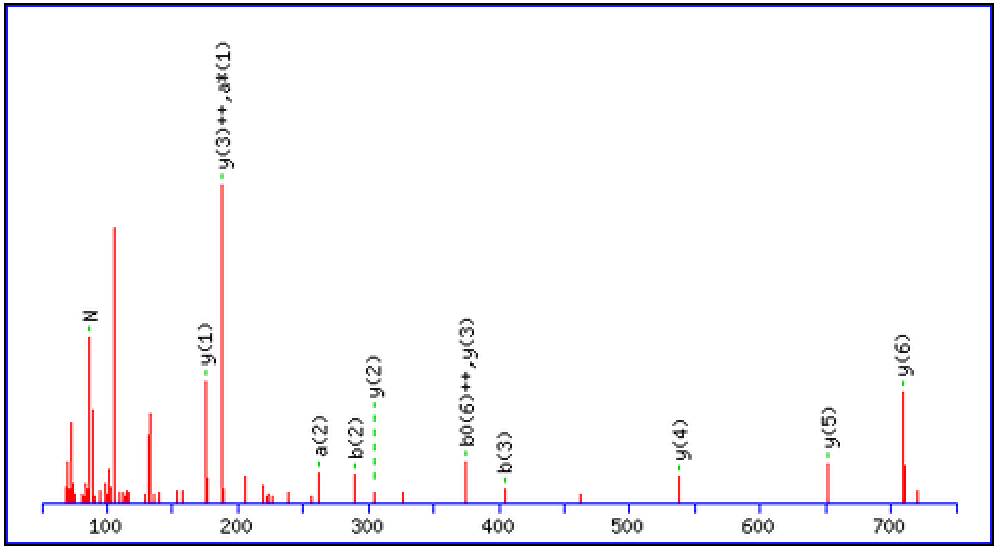

Monoisotopic mass of neutral peptide Mr(calc): 940.44
Variable modifications:
K1 : Benzoyl (K)
Ions Score: 50 **Expect:** 0.0012
Matches (Bold Red): 13/113 fragment ions using 14 most intense peaks

#	Immon.	a	a^{++}	a^{*}	a^{*++}	a^{0}	a^{0++}	b	b^{++}	b^{*}	b^{*++}	b^{0}	b^{0++}	Seq.	y	y^{++}	y^{*}	y^{*++}	y^{0}	y^{0++}	#
1	205.13	205.13	103.07	**188.11**	94.56			233.13	117.07	216.10	108.55			K							7
2	30.03	**262.15**	131.58	245.13	123.07			**290.15**	145.58	273.12	137.07			G	**709.33**	355.17	692.30	346.65	691.32	346.16	6
3	**87.06**	376.20	188.60	359.17	180.09			**404.19**	202.60	387.17	194.09			N	**652.30**	326.66	635.28	318.14	634.29	317.65	5
4	136.08	539.26	270.13	522.23	261.62			567.26	284.13	550.23	275.62			Y	**538.26**	269.63	521.24	261.12	520.25	260.63	4
5	44.05	610.30	305.65	593.27	297.14			638.29	319.65	621.27	311.14			A	**375.20**	**188.10**	358.17	179.59	357.19	179.10	3
6	102.05	739.34	370.17	722.31	361.66	721.33	361.17	767.34	384.17	750.31	375.66	749.33	**375.17**	E	**304.16**	152.58	287.13	144.07	286.15	143.58	2
7	129.11													R	**175.12**	88.06	158.09	79.55			1

MS/MS Fragmentation of **PEPTKSAPAPK**

Found in **P58876**, Histone H2B type 1-D

Match to Query 4279: 1225.635042 from(613.824797,2+)

H2BK5bz

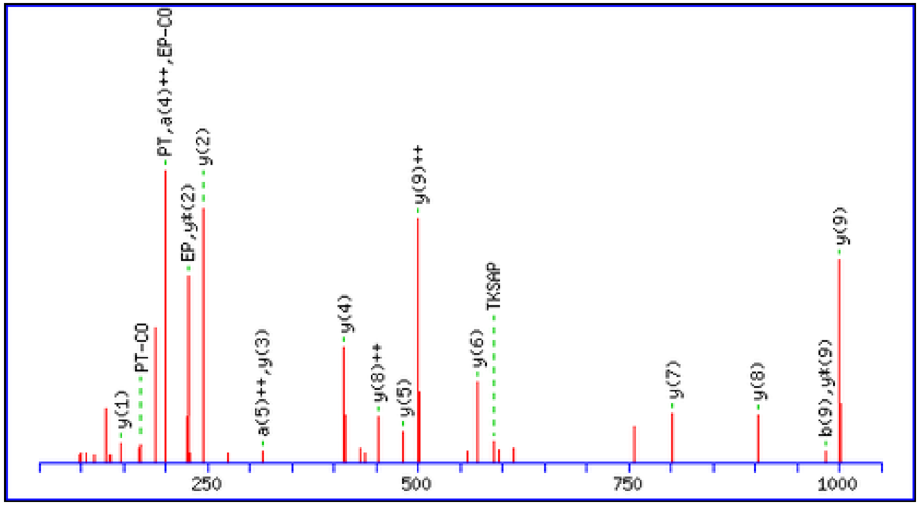

Monoisotopic mass of neutral peptide Mr(calc): 1225.63
Variable modifications:
K5 : Benzoyl (K)
Ions Score: 70 **Expect:** 6e-005
Matches (Bold Red): 24/220 fragment ions using 25 most intense peaks

#	Immon.	a	a^{++}	a*	a^{*++}	a^0	a^{0++}	b	b^{++}	b*	b^{*++}	b^0	b^{0++}	Seq.	y	y^{++}	y*	y^{*++}	y^0	y^{0++}	#
1	70.07	70.07	35.54					98.06	49.53					P							11
2	102.05	**199.11**	100.06			181.10	91.05	**227.10**	114.05			209.09	105.05	E	1129.59	565.30	1112.56	556.78	1111.58	556.29	10
3	70.07	296.16	148.58			278.15	139.58	324.16	162.58			306.14	153.58	P	**1000.55**	**500.78**	**983.52**	492.26	982.54	491.77	9
4	74.06	397.21	**199.11**			379.20	190.10	425.20	213.11			407.19	204.10	T	**903.49**	**452.25**	886.47	443.74	885.48	443.25	8
5	205.13	629.33	**315.17**	612.30	306.66	611.32	306.16	657.32	329.17	640.30	320.65	639.31	320.16	K	**802.45**	401.73	785.42	393.21	784.44	392.72	7
6	60.04	716.36	358.68	699.33	350.17	698.35	349.68	744.36	372.68	727.33	364.17	726.35	363.68	S	**570.32**	285.67	553.30	277.15	552.31	276.66	6
7	44.05	787.40	394.20	770.37	385.69	769.39	385.20	815.39	408.20	798.37	399.69	797.38	399.20	A	**483.29**	242.15	466.27	233.64			5
8	70.07	884.45	442.73	867.42	434.22	866.44	433.72	912.45	456.73	895.42	448.21	894.44	447.72	P	**412.26**	206.63	395.23	198.12			4
9	44.05	955.49	478.25	938.46	469.73	937.48	469.24	**983.48**	492.25	966.46	483.73	965.47	483.24	A	**315.20**	158.10	298.18	149.59			3
10	70.07	1052.54	526.77	1035.51	518.26	1034.53	517.77	1080.54	540.77	1063.51	532.26	1062.53	531.77	P	**244.17**	122.59	**227.14**	114.07			2
11	101.11													K	**147.11**	74.06	130.09	65.55			1

MS/MS Fragmentation of **SAPAPKK**

Found in **P58876**, Histone H2B type 1-D

Match to Query 692: 801.440312 from(401.727432,2+)

H2BK11bz

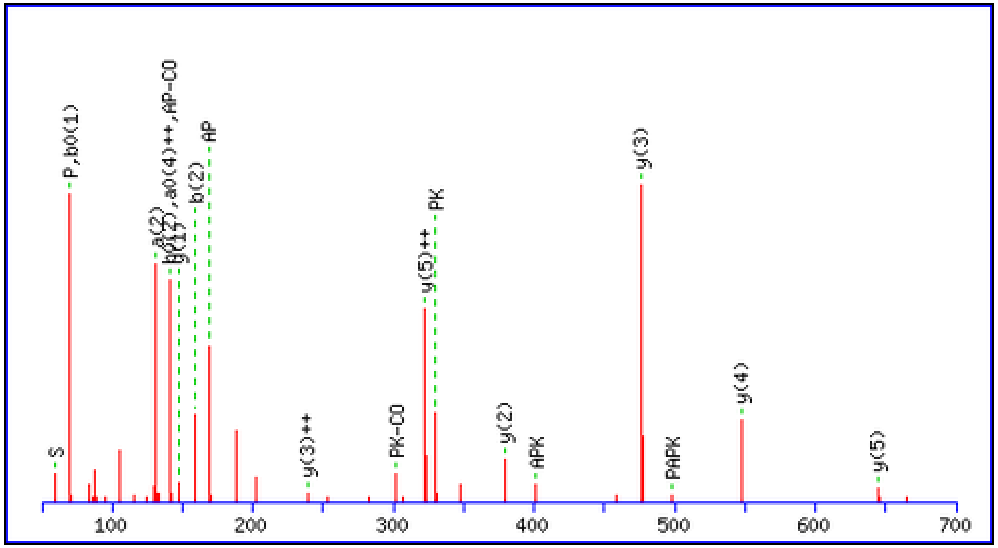

Monoisotopic mass of neutral peptide Mr(calc): 801.44

Variable modifications:

K6 : Benzoyl (K)

Ions Score: 24 **Expect:** 2.9

Matches (Bold Red): 26/103 fragment ions using 33 most intense peaks

#	Immon.	a	a++	a*	a*++	a0	a0++	b	b++	b*	b*++	b0	b0++	Seq.	y	y++	y*	y*++	#
1	**60.04**	**60.04**	30.53			42.03	21.52	88.04	44.52			**70.03**	35.52	S					7
2	44.05	**131.08**	66.04			113.07	57.04	**159.08**	80.04			**141.07**	71.04	A	715.41	358.21	698.39	349.70	6
3	**70.07**	228.13	114.57			210.12	105.57	256.13	128.57			238.12	119.56	P	**644.38**	**322.69**	627.35	314.18	5
4	44.05	299.17	150.09			281.16	**141.08**	327.17	164.09			309.16	155.08	A	**547.32**	274.17	530.30	265.65	4
5	**70.07**	396.22	198.62			378.21	189.61	424.22	212.61			406.21	203.61	P	**476.29**	**238.65**	459.26	230.13	3
6	205.13	628.35	314.68	611.32	306.16	610.33	305.67	656.34	328.67	639.31	320.16	638.33	319.67	K	**379.23**	190.12	362.21	181.61	2
7	101.11													K	**147.11**	74.06	130.09	65.55	1

MS/MS Fragmentation of **KAVTKAQK**
Found in **P58876**, Histone H2B type 1-D
Match to Query 2722: 1018.581420 from(510.297986,2+)

H2BK16bz

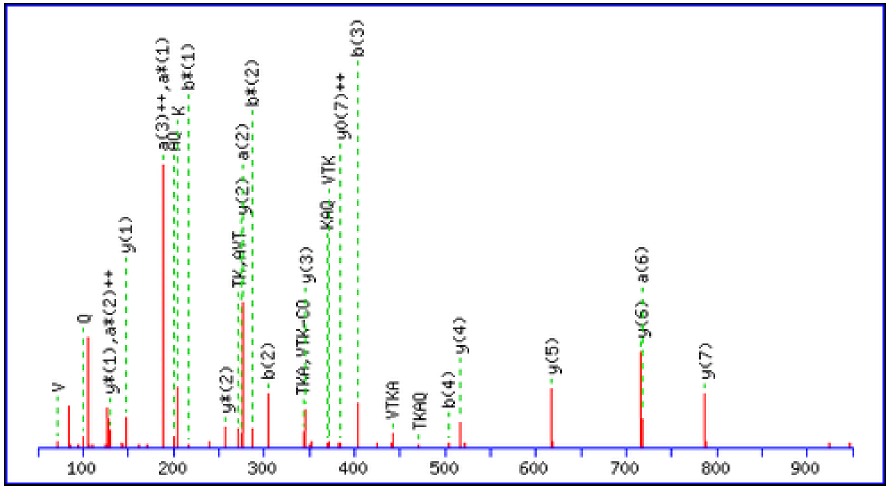

Monoisotopic mass of neutral peptide Mr(calc): 1018.58
Variable modifications:
K1 : Benzoyl (K)
K5 : Acetyl (K)
Ions Score: 39 **Expect:** 0.11
Matches (Bold Red): 35/144 fragment ions using 46 most intense peaks

#	Immon.	a	a^{++}	a^{*}	a^{*++}	a^{0}	a^{0++}	b	b^{++}	b^{*}	b^{*++}	b^{0}	b^{0++}	Seq.	y	y^{++}	y^{*}	y^{*++}	y^{0}	y^{0++}	#
1	**205.13**	**205.13**	103.07	**188.11**	94.56			233.13	117.07	**216.10**	108.55			K							8
2	44.05	**276.17**	138.59	259.14	**130.08**			**304.17**	152.59	**287.14**	144.07			A	**787.47**	394.24	770.44	385.72	769.46	**385.23**	7
3	**72.08**	375.24	**188.12**	358.21	179.61			**403.23**	202.12	386.21	193.61			V	**716.43**	358.72	699.40	350.21	698.42	349.71	6
4	74.06	476.29	238.65	459.26	230.13	458.28	229.64	**504.28**	252.64	487.26	244.13	486.27	243.64	T	**617.36**	309.18	600.34	300.67	599.35	300.18	5
5	143.12	646.39	323.70	629.37	315.19	628.38	314.69	674.39	337.70	657.36	329.18	656.38	328.69	K	**516.31**	258.66	499.29	250.15			4
6	44.05	**717.43**	359.22	700.40	350.71	699.42	350.21	745.42	373.22	728.40	364.70	727.41	364.21	A	**346.21**	173.61	329.18	165.09			3
7	**101.07**	845.49	423.25	828.46	414.73	827.48	414.24	873.48	437.25	856.46	428.73	855.47	428.24	Q	**275.17**	138.09	**258.14**	129.58			2
8	**101.11**													K	**147.11**	74.06	**130.09**	65.55			1

MS/MS Fragmentation of **KAVTKAQK**

Found in **P58876**, Histone H2B type 1-D

Match to Query 2721: 1018.580298 from(510.297425,2+)

H2BK20bz

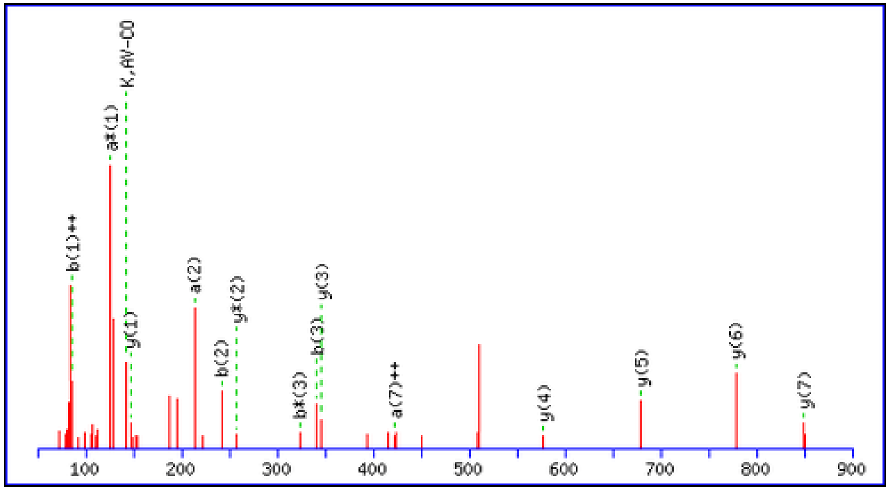

Monoisotopic mass of neutral peptide Mr(calc): 1018.58

Variable modifications:

K1 : Acetyl (K)

K5 : Benzoyl (K)

Ions Score: 34 **Expect:** 0.33

Matches (Bold Red): 17/143 fragment ions using 30 most intense peaks

#	Immon.	a	a^{++}	a^{*}	a^{*++}	a^{0}	a^{0++}	b	b^{++}	b^{*}	b^{*++}	b^{0}	b^{0++}	Seq.	y	y^{++}	y^{*}	y^{*++}	y^{0}	y^{0++}	#
1	**143.12**	**143.12**	72.06	**126.09**	63.55			171.11	**86.06**	154.09	77.55			K							8
2	44.05	**214.16**	107.58	197.13	99.07			**242.15**	121.58	225.12	113.07			A	**849.48**	425.25	832.46	416.73	831.47	416.24	7
3	72.08	313.22	157.12	296.20	148.60			**341.22**	171.11	**324.19**	162.60			V	**778.45**	389.73	761.42	381.21	760.44	380.72	6
4	74.06	414.27	207.64	397.24	199.13	396.26	198.63	442.27	221.64	425.24	213.12	424.26	212.63	T	**679.38**	340.19	662.35	331.68	661.37	331.19	5
5	205.13	646.39	323.70	629.37	315.19	628.38	314.69	674.39	337.70	657.36	329.18	656.38	328.69	K	**578.33**	289.67	561.30	281.16			4
6	44.05	717.43	359.22	700.40	350.71	699.42	350.21	745.42	373.22	728.40	364.70	727.41	364.21	A	**346.21**	173.61	329.18	165.09			3
7	101.07	845.49	**423.25**	828.46	414.73	827.48	414.24	873.48	437.25	856.46	428.73	855.47	428.24	Q	275.17	138.09	**258.14**	129.58			2
8	101.11													K	**147.11**	74.06	130.09	65.55			1

MS/MS Fragmentation of **KSAGAAKR**

Found in **P10412**, Histone H1.4

Match to Query 1507: 947.518172 from(474.766362,2+)

H1K25bz

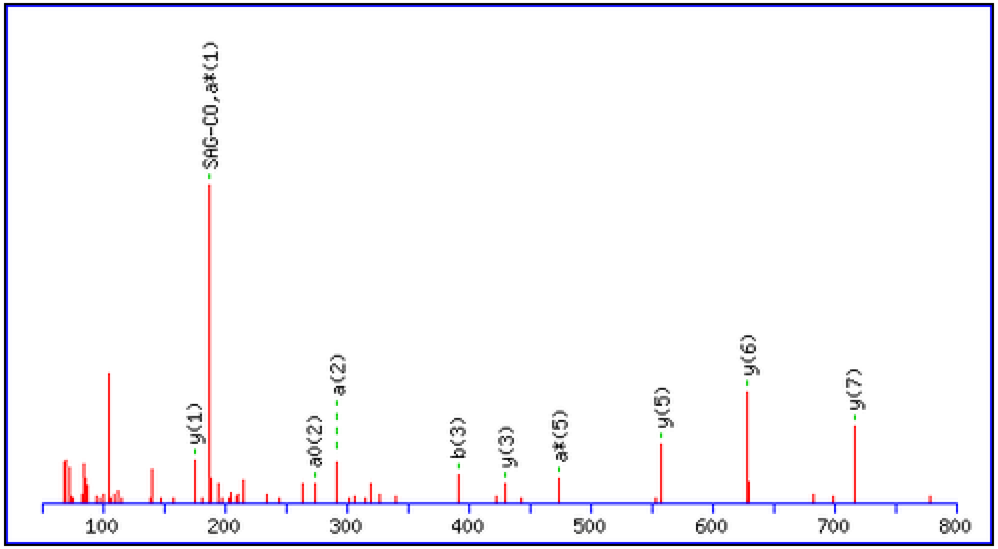

Monoisotopic mass of neutral peptide Mr(calc): 947.52

Variable modifications:

K1 : Benzoyl (K)

K7 : Propionyl (K)

Ions Score: 37 **Expect:** 0.14

Matches (Bold Red): 11/148 fragment ions using 17 most intense peaks

#	Immon.	a	a^{++}	a^*	a^{*++}	a^0	a^{0++}	b	b^{++}	b^*	b^{*++}	b^0	b^{0++}	Seq.	y	y^{++}	y^*	y^{*++}	y^0	y^{0++}	#
1	205.13	205.13	103.07	**188.11**	94.56			233.13	117.07	216.10	108.55			K							8
2	60.04	**292.17**	146.59	275.14	138.07	**274.16**	137.58	320.16	160.58	303.13	152.07	302.15	151.58	S	**716.40**	358.71	699.38	350.19	698.39	349.70	7
3	44.05	363.20	182.10	346.18	173.59	345.19	173.10	**391.20**	196.10	374.17	187.59	373.19	187.10	A	**629.37**	315.19	612.35	306.68			6
4	30.03	420.22	210.62	403.20	202.10	402.21	201.61	448.22	224.61	431.19	216.10	430.21	215.61	G	**558.34**	279.67	541.31	271.16			5
5	44.05	491.26	246.13	**474.23**	237.62	473.25	237.13	519.26	260.13	502.23	251.62	501.25	251.13	A	501.31	251.16	484.29	242.65			4
6	44.05	562.30	281.65	545.27	273.14	544.29	272.65	590.29	295.65	573.27	287.14	572.28	286.64	A	**430.28**	215.64	413.25	207.13			3
7	157.13	746.42	373.71	729.39	365.20	728.41	364.71	774.41	387.71	757.39	379.20	756.40	378.71	K	359.24	180.12	342.21	171.61			2
8	129.11													R	**175.12**	88.06	158.09	79.55			1

MS/MS Fragmentation of **KASGPPVSELITK**

Found in **P15864**, Histone H1.2

Match to Query 8982: 1429.783604 from(715.899078,2+)

H1K33bz

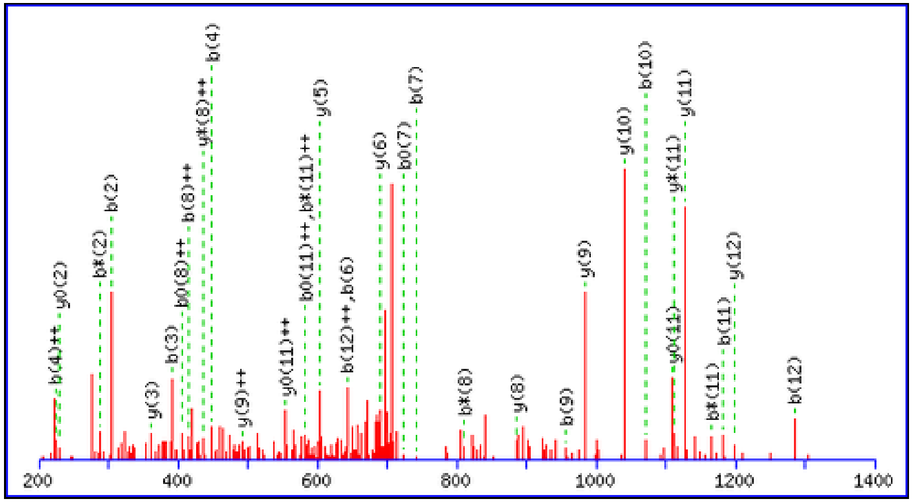

Monoisotopic mass of neutral peptide Mr(calc): 1429.78

Variable modifications:

K1 : Benzoyl (K)

Ions Score: 36 **Expect:** 0.31

Matches (Bold Red): 33/138 fragment ions using 87 most i[illegible]

#	b	b^{++}	b^{*}	b^{*++}	b^{0}	b^{0++}	Seq.	y	y^{++}	y^{*}	y^{*++}	y^{0}	y^{0++}	#
1	233.13	117.07	216.10	108.55			K							13
2	**304.17**	152.59	**287.14**	144.07			A	**1198.67**	599.84	1181.64	591.32	1180.66	590.83	12
3	**391.20**	196.10	374.17	187.59	373.19	187.10	S	**1127.63**	564.32	**1110.60**	555.81	**1109.62**	**555.31**	11
4	**448.22**	**224.61**	431.19	216.10	430.21	215.61	G	**1040.60**	520.80	1023.57	512.29	1022.59	511.80	10
5	545.27	273.14	528.25	264.63	527.26	264.13	P	**983.58**	**492.29**	966.55	483.78	965.57	483.29	9
6	**642.32**	321.67	625.30	313.15	624.31	312.66	P	**886.52**	443.77	869.50	**435.25**	868.51	434.76	8
7	**741.39**	371.20	724.37	362.69	**723.38**	362.19	V	789.47	395.24	772.45	386.73	771.46	386.23	7
8	828.43	**414.72**	**811.40**	406.20	810.41	**405.71**	S	**690.40**	345.71	673.38	337.19	672.39	336.70	6
9	**957.47**	479.24	940.44	470.72	939.46	470.23	E	**603.37**	302.19	586.34	293.68	585.36	293.18	5
10	**1070.55**	535.78	1053.53	527.27	1052.54	526.77	L	474.33	237.67	457.30	229.15	456.32	228.66	4
11	**1183.64**	592.32	**1166.61**	**583.81**	1165.63	**583.32**	I	**361.24**	181.13	344.22	172.61	343.23	172.12	3
12	**1284.68**	**642.85**	1267.66	634.33	1266.67	633.84	T	248.16	124.58	231.13	116.07	**230.15**	115.58	2
13							K	147.11	74.06	130.09	65.55			1

MS/MS Fragmentation of **GTGASGSFKLNK**

Found in **P15864**, Histone H1.2

Match to Query 7645: 1269.635164 from(635.824858,2+)

H1K104bz

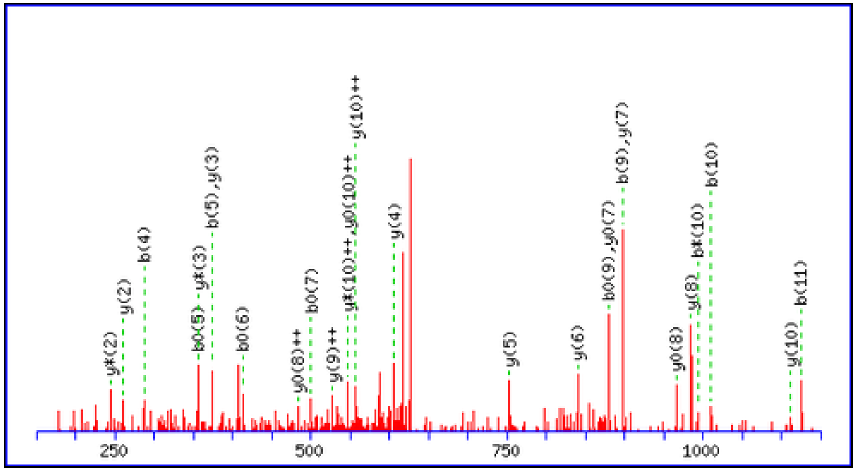

Monoisotopic mass of neutral peptide Mr(calc): 1269.64

Variable modifications:

K9 : Benzoyl (K)

Ions Score: 53 **Expect:** 0.0029

Matches (Bold Red): 27/104 fragment ions using 43 most intense peaks

#	b	b^{++}	b^{*}	b^{*++}	b^{0}	b^{0++}	Seq.	y	y^{++}	y^{*}	y^{*++}	y^{0}	y^{0++}	#
1	58.03	29.52					G							12
2	159.08	80.04			141.07	71.04	T	1213.62	607.31	1196.59	598.80	1195.61	598.31	11
3	216.10	108.55			198.09	99.55	G	**1112.57**	**556.79**	1095.55	**548.28**	1094.56	**547.79**	10
4	**287.13**	144.07			269.12	135.07	A	1055.55	**528.28**	1038.53	519.77	1037.54	519.27	9
5	**374.17**	187.59			**356.16**	178.58	S	**984.51**	492.76	967.49	484.25	**966.50**	**483.76**	8
6	431.19	216.10			**413.18**	207.09	G	**897.48**	449.25	880.46	440.73	**879.47**	440.24	7
7	518.22	259.61			**500.21**	250.61	S	**840.46**	420.73	823.43	412.22	822.45	411.73	6
8	665.29	333.15			647.28	324.14	F	**753.43**	377.22	736.40	368.71			5
9	**897.41**	449.21	880.38	440.70	**879.40**	440.20	K	**606.36**	303.68	589.33	295.17			4
10	**1010.49**	505.75	**993.47**	497.24	992.48	496.75	L	**374.24**	187.62	**357.21**	179.11			3
11	**1124.54**	562.77	1107.51	554.26	1106.53	553.77	N	**261.16**	131.08	**244.13**	122.57			2
12							K	147.11	74.06	130.09	65.55			1

MS/MS Fragmentation of **ATGAATPKK**

Found in **P10412**, Histone H1.4

Match to Query 2014: 947.505448 from(474.760000,2+)

H1K147bz

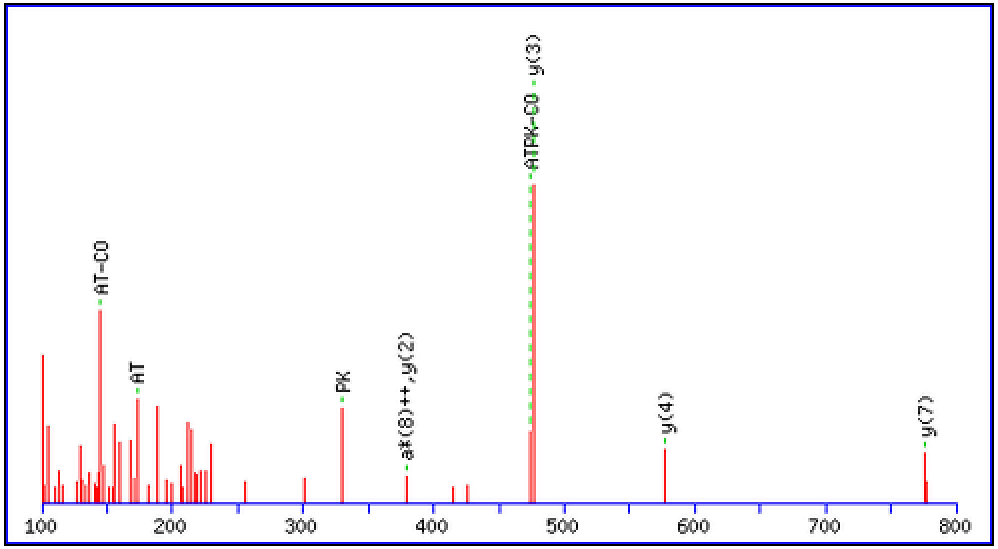

Monoisotopic mass of neutral peptide Mr(calc): 947.51

Variable modifications:

K8 : Benzoyl (K)

Ions Score: 33 **Expect:** 0.35

Matches (Bold Red): 11/155 fragment ions using 10 most intense peaks

#	Immon.	a	a^{++}	a^{*}	a^{*++}	a^{0}	a^{0++}	b	b^{++}	b^{*}	b^{*++}	b^{0}	b^{0++}	Seq.	y	y^{++}	y^{*}	y^{*++}	y^{0}	y^{0++}	#
1	44.05	44.05	22.53					72.04	36.53					A							9
2	74.06	**145.10**	73.05			127.09	64.05	**173.09**	87.05			155.08	78.04	T	877.48	439.24	860.45	430.73	859.47	430.24	8
3	30.03	202.12	101.56			184.11	92.56	230.11	115.56			212.10	106.56	G	**776.43**	388.72	759.40	380.21	758.42	379.71	7
4	44.05	273.16	137.08			255.15	128.08	301.15	151.08			283.14	142.07	A	719.41	360.21	702.38	351.69	701.40	351.20	6
5	44.05	344.19	172.60			326.18	163.59	372.19	186.60			354.18	177.59	A	648.37	324.69	631.34	316.18	630.36	315.68	5
6	74.06	445.24	223.12			427.23	214.12	473.24	237.12			455.22	228.12	T	**577.33**	289.17	560.31	280.66	559.32	280.17	4
7	70.07	542.29	271.65			524.28	262.64	570.29	285.65			552.28	276.64	P	**476.29**	238.65	459.26	230.13			3
8	205.13	774.41	387.71	757.39	**379.20**	756.40	378.71	802.41	401.71	785.38	393.20	784.40	392.70	K	**379.23**	190.12	362.21	181.61			2
9	101.11													K	147.11	74.06	130.09	65.55			1

MS/MS Fragmentation of **SASKAVKPK**

Found in **P15864**, Histone H1.2

Match to Query 4286: 1018.581214 from(510.297883,2+)

H1K190bz

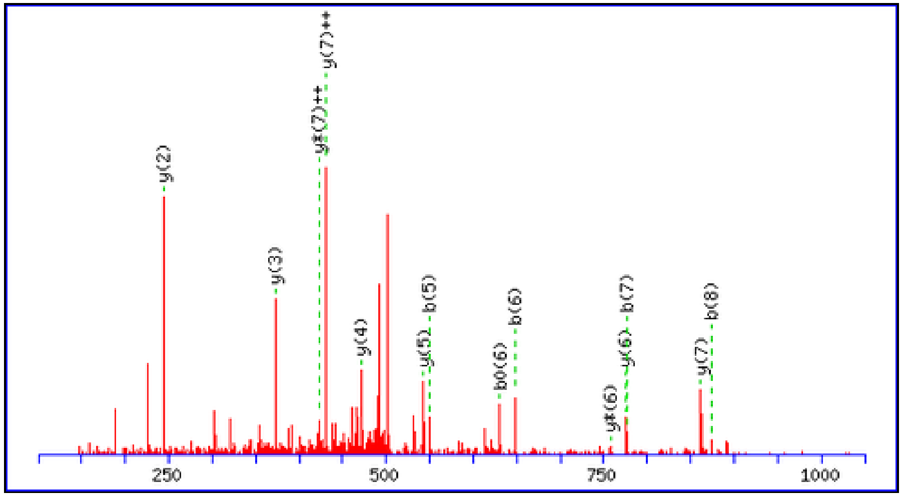

Monoisotopic mass of neutral peptide Mr(calc): 1018.58
Variable modifications:
K4 : Benzoyl (K)
Ions Score: 30 **Expect:** 0.91
Matches (Bold Red): 14/78 fragment ions using 37 most intense peaks

#	b	b^{++}	b^{*}	b^{*++}	b^{0}	b^{0++}	Seq.	y	y^{++}	y^{*}	y^{*++}	y^{0}	y^{0++}	#
1	88.04	44.52			70.03	35.52	S							9
2	159.08	80.04			141.07	71.04	A	932.56	466.78	915.53	458.27	914.55	457.78	8
3	246.11	123.56			228.10	114.55	S	**861.52**	**431.26**	844.49	**422.75**	843.51	422.26	7
4	478.23	239.62	461.20	231.11	460.22	230.61	K	**774.49**	387.75	**757.46**	379.23			6
5	**549.27**	275.14	532.24	266.62	531.26	266.13	A	**542.37**	271.69	525.34	263.17			5
6	**648.34**	324.67	631.31	316.16	**630.32**	315.67	V	**471.33**	236.17	454.30	227.65			4
7	**776.43**	388.72	759.40	380.21	758.42	379.71	K	**372.26**	186.63	355.23	178.12			3
8	**873.48**	437.25	856.46	428.73	855.47	428.24	P	**244.17**	122.59	227.14	114.07			2
9							K	147.11	74.06	130.09	65.55			1

Supplementary Figure 2. MS/MS spectra of histone K_{bz}-containing peptides in HepG2 cells. Annotated MS/MS spectra are provided for each detected K_{bz} peptide. In each spectra, the major ions were found to be the y- and b-ions (C- and N-terminal fragments cleaved across the peptide bond, respectively).

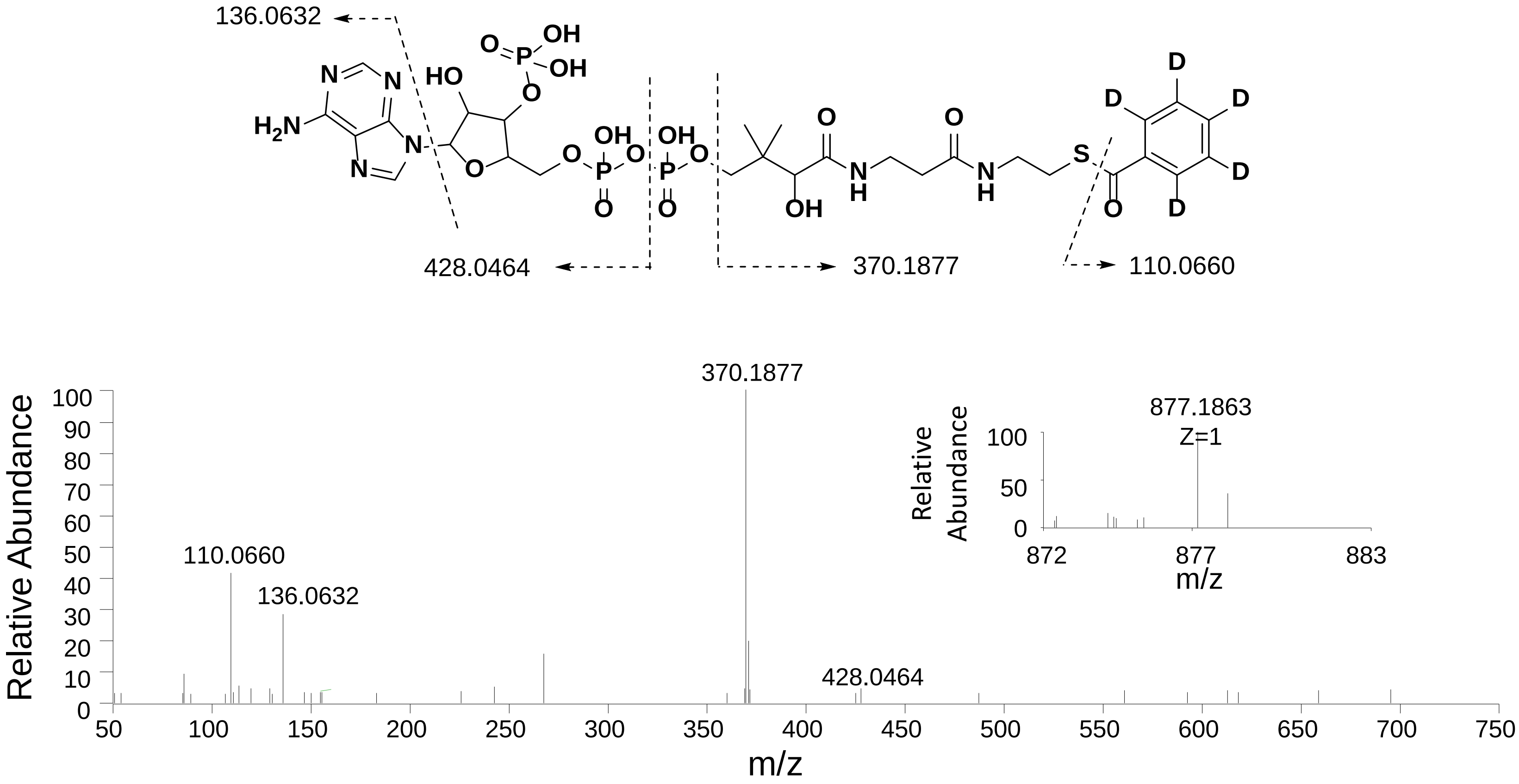

Supplementary Figure 3. Spectrum of D_5-Benzoyl-CoA

MS/MS Fragmentation of **TKQTAR**

Found in **K7EK07**, Histone H3 (Fragment)

Match to Query 834: 812.454790 from(407.234671,2+)

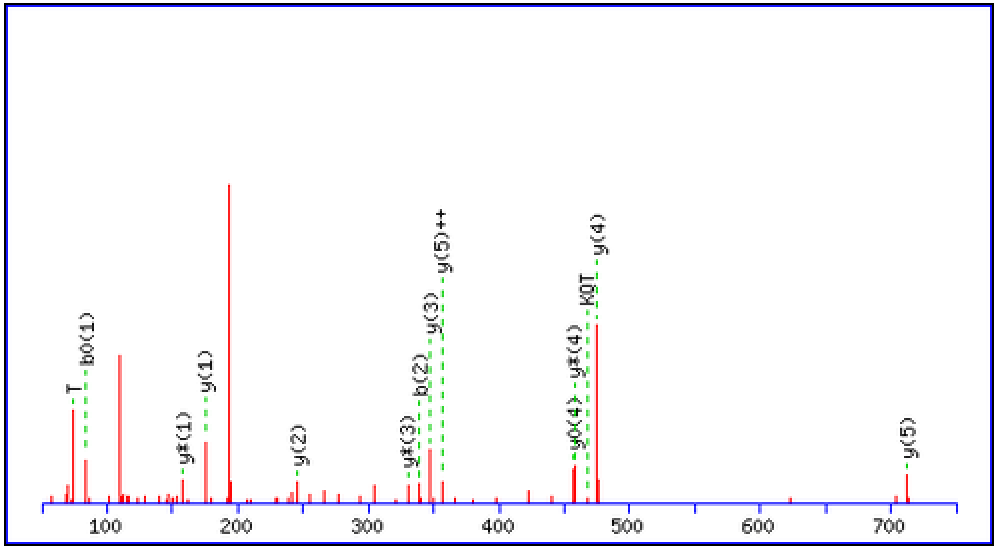

H3K4bz

Monoisotopic mass of neutral peptide Mr(calc): 812.46
Variable modifications:
K2 : Benzoyl-D5 (K)
Ions Score: 33 **Expect:** 0.17
Matches (Bold Red): 17/100 fragment ions using 24 most intense peaks

#	Immon.	a	a^{++}	a^{*}	a^{*++}	a^{0}	a^{0++}	b	b^{++}	b^{*}	b^{*++}	b^{0}	b^{0++}	Seq.	y	y^{++}	y^{*}	y^{*++}	y^{0}	y^{0++}	#
1	**74.06**	**74.06**	37.53			56.05	28.53	102.05	51.53			**84.04**	42.53	T							6
2	210.16	311.21	156.11	294.19	147.60	293.20	147.10	**339.21**	170.11	322.18	161.59	321.20	161.10	K	**712.41**	**356.71**	695.39	348.20	694.40	347.71	5
3	101.07	439.27	220.14	422.24	211.63	421.26	211.13	**467.27**	234.14	450.24	225.62	449.26	225.13	Q	**475.26**	238.13	**458.24**	229.62	**457.25**	229.13	4
4	**74.06**	540.32	270.66	523.29	262.15	522.31	261.66	568.31	284.66	551.29	276.15	550.30	275.66	T	**347.20**	174.11	**330.18**	165.59	329.19	165.10	3
5	44.05	611.36	306.18	594.33	297.67	593.35	297.18	639.35	320.18	622.32	311.67	621.34	311.17	A	**246.16**	123.58	229.13	115.07			2
6	129.11													R	**175.12**	88.06	**158.09**	79.55			1

MS/MS Fragmentation of **KSTGGKAPR**

Found in **Q71DI3**, Histone H3.2

Match to Query 2240: 1051.582002 from(526.798277,2+)

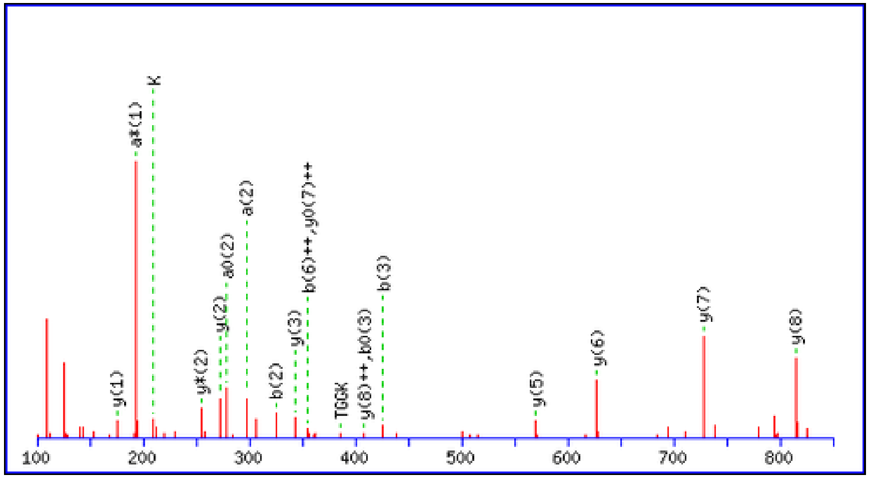

H3K9bz

Monoisotopic mass of neutral peptide Mr(calc): 1051.58
Variable modifications:
K1 : Benzoyl-D5 (K)
K6 : Acetyl (K)
Ions Score: 41 **Expect:** 0.069
Matches (Bold Red): 20/179 fragment ions using 35 most intense peaks

#	Immon.	a	a^{++}	a^{*}	a^{*++}	a^{0}	a^{0++}	b	b^{++}	b^{*}	b^{*++}	b^{0}	b^{0++}	Seq.	y	y^{++}	y^{*}	y^{*++}	y^{0}	y^{0++}	#
1	**210.16**	**210.16**	105.59	**193.14**	97.07			238.16	119.58	221.13	111.07			K							9
2	60.04	**297.20**	149.10	280.17	140.59	**279.19**	140.10	**325.19**	163.10	308.17	154.59	307.18	154.09	S	**815.44**	**408.22**	798.41	399.71	797.43	399.22	8
3	74.06	398.24	199.63	381.22	191.11	380.23	190.62	**426.24**	213.62	409.21	205.11	**408.23**	204.62	T	**728.40**	364.71	711.38	356.19	710.39	**355.70**	7
4	30.03	455.27	228.14	438.24	219.62	437.26	219.13	483.26	242.13	466.23	233.62	465.25	233.13	G	**627.36**	314.18	610.33	305.67			6
5	30.03	512.29	256.65	495.26	248.13	494.28	247.64	540.28	270.64	523.26	262.13	522.27	261.64	G	**570.34**	285.67	553.31	277.16			5
6	143.12	682.39	341.70	665.37	333.19	664.38	332.69	710.39	**355.70**	693.36	347.18	692.38	346.69	K	513.31	257.16	496.29	248.65			4
7	44.05	753.43	377.22	736.40	368.71	735.42	368.21	781.43	391.22	764.40	382.70	763.41	382.21	A	**343.21**	172.11	326.18	163.59			3
8	70.07	850.48	425.75	833.46	417.23	832.47	416.74	878.48	439.74	861.45	431.23	860.47	430.74	P	**272.17**	136.59	**255.15**	128.08			2
9	129.11													R	**175.12**	88.06	158.09	79.55			1

MS/MS Fragmentation of **STGGKAPR**

Found in **K7EK07**, Histone H3 (Fragment)

Match to Query 1379: 881.476600 from(441.745576,2+)

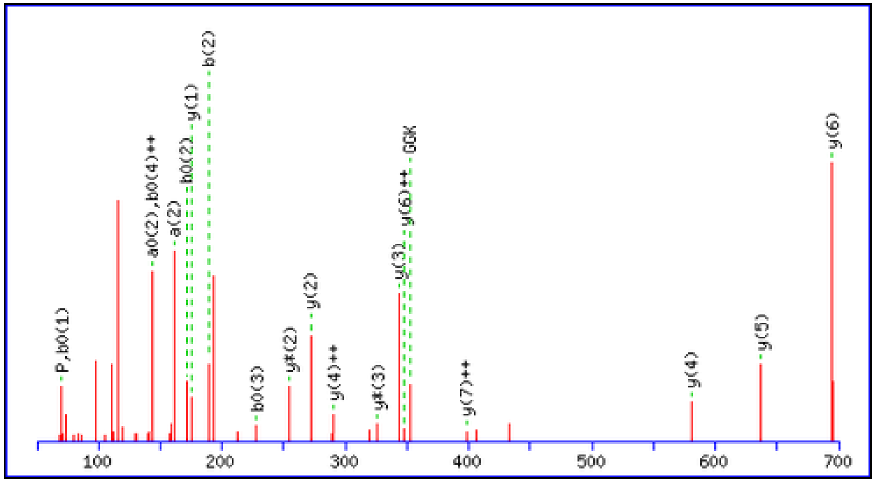

H3K14bz

Monoisotopic mass of neutral peptide Mr(calc): 881.48
Variable modifications:
K5 : Benzoyl-D5 (K)
Ions Score: 50 **Expect:** 0.0054
Matches (Bold Red): 20/136 fragment ions using 25 most intense peaks

#	Immon.	a	a++	a*	a*++	a0	a0++	b	b++	b*	b*++	b0	b0++	Seq.	y	y++	y*	y*++	y0	y0++	#
1	60.04	60.04	30.53			42.03	21.52	88.04	44.52			**70.03**	35.52	S							8
2	74.06	**161.09**	81.05			**143.08**	72.04	**189.09**	95.05			**171.08**	86.04	T	795.45	**398.23**	778.43	389.72	777.44	389.22	7
3	30.03	218.11	109.56			200.10	100.56	246.11	123.56			**228.10**	114.55	G	**694.40**	**347.71**	677.38	339.19			6
4	30.03	275.13	138.07			257.12	129.07	303.13	152.07			285.12	**143.06**	G	**637.38**	319.20	620.36	310.68			5
5	210.16	512.29	256.65	495.26	248.13	494.28	247.64	540.28	270.64	523.26	262.13	522.27	261.64	K	**580.36**	**290.68**	563.33	282.17			4
6	44.05	583.32	292.17	566.30	283.65	565.31	283.16	611.32	306.16	594.29	297.65	593.31	297.16	A	**343.21**	172.11	**326.18**	163.59			3
7	**70.07**	680.38	340.69	663.35	332.18	662.37	331.69	708.37	354.69	691.35	346.18	690.36	345.68	P	**272.17**	136.59	**255.15**	128.08			2
8	129.11													R	**175.12**	88.06	158.09	79.55			1

MS/MS Fragmentation of **KQLATKAAR**
Found in **Q71DI3**, Histone H3.2
Match to Query 2736: 1136.671004 from(569.342778,2+)

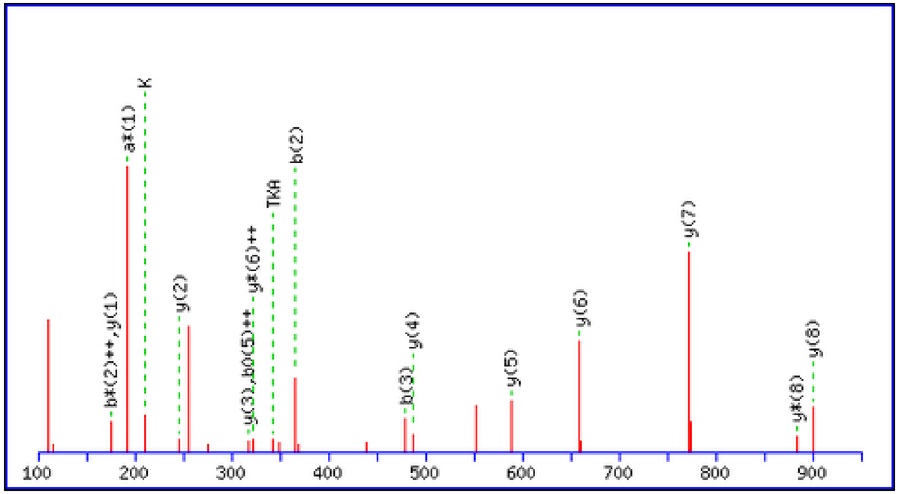

H3K18bz

Monoisotopic mass of neutral peptide Mr(calc): 1136.67
Variable modifications:
K1 : Benzoyl-D5 (K)
K6 : Acetyl (K)
Ions Score: 58 **Expect:** 0.0026
Matches (Bold Red): 19/170 fragment ions using 27 most intense peaks

#	Immon.	a	a^{++}	a^*	a^{*++}	a^0	a^{0++}	b	b^{++}	b^*	b^{*++}	b^0	b^{0++}	Seq.	y	y^{++}	y^*	y^{*++}	y^0	y^{0++}	#
1	**210.16**	**210.16**	105.59	**193.14**	97.07			238.16	119.58	221.13	111.07			K							9
2	101.07	338.22	169.62	321.20	161.10			**366.22**	183.61	349.19	**175.10**			Q	**900.53**	450.77	**883.50**	442.25	882.52	441.76	8
3	86.10	451.31	226.16	434.28	217.64			**479.30**	240.15	462.28	231.64			L	**772.47**	386.74	755.44	378.22	754.46	377.73	7
4	44.05	522.34	261.68	505.32	253.16			550.34	275.67	533.31	267.16			A	**659.38**	330.20	642.36	**321.68**	641.37	321.19	6
5	74.06	623.39	312.20	606.37	303.69	605.38	303.19	651.39	326.20	634.36	317.68	633.38	**317.19**	T	**588.35**	294.68	571.32	286.16	570.34	285.67	5
6	143.12	793.50	397.25	776.47	388.74	775.49	388.25	821.49	411.25	804.47	402.74	803.48	402.24	K	**487.30**	244.15	470.27	235.64			4
7	44.05	864.53	432.77	847.51	424.26	846.52	423.77	892.53	446.77	875.50	438.26	874.52	437.76	A	**317.19**	159.10	300.17	150.59			3
8	44.05	935.57	468.29	918.55	459.78	917.56	459.28	963.57	482.29	946.54	473.77	945.56	473.28	A	**246.16**	123.58	229.13	115.07			2
9	129.11													R	**175.12**	88.06	158.09	79.55			1

MS/MS Fragmentation of **KQLATKAAR**
Found in **K7EK07**, Histone H3 (Fragment)
Match to Query 2928: 1094.660592 from(548.337572,2+)

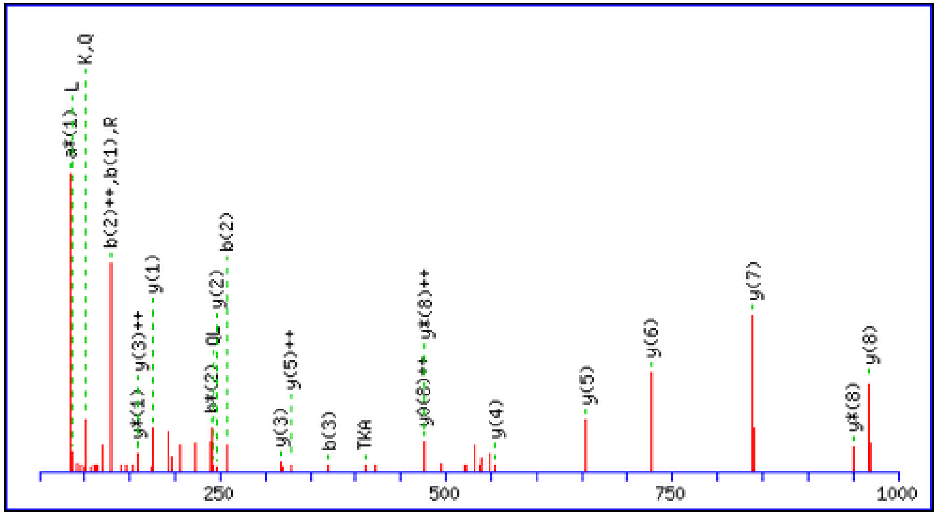

H3K23bz

Monoisotopic mass of neutral peptide Mr(calc): 1094.66
Variable modifications:
K6 : Benzoyl-D5 (K)
Ions Score: 53 **Expect:** 0.0021
Matches (Bold Red): 28/168 fragment ions using 43 most intense peaks

#	Immon.	a	a^{++}	a^{*}	a^{*++}	a^{0}	a^{0++}	b	b^{++}	b^{*}	b^{*++}	b^{0}	b^{0++}	Seq.	y	y^{++}	y^{*}	y^{*++}	y^{0}	y^{0++}	#
1	**101.11**	**101.11**	51.06	**84.08**	42.54			**129.10**	65.05	112.08	56.54			K							9
2	**101.07**	229.17	115.09	212.14	106.57			**257.16**	**129.08**	**240.13**	120.57			Q	**967.57**	484.29	**950.55**	**475.78**	949.56	**475.28**	8
3	**86.10**	342.25	171.63	325.22	163.12			**370.24**	185.63	353.22	177.11			L	**839.51**	420.26	822.49	411.75	821.50	411.26	7
4	44.05	413.29	207.15	396.26	198.63			441.28	221.14	424.26	212.63			A	**726.43**	363.72	709.40	355.21	708.42	354.71	6
5	74.06	514.33	257.67	497.31	249.16	496.32	248.67	542.33	271.67	525.30	263.16	524.32	262.66	T	**655.39**	**328.20**	638.37	319.69	637.38	319.20	5
6	210.16	751.49	376.25	734.46	367.73	733.48	367.24	779.48	390.24	762.46	381.73	761.47	381.24	K	**554.35**	277.68	537.32	269.16			4
7	44.05	822.52	411.77	805.50	403.25	804.51	402.76	850.52	425.76	833.49	417.25	832.51	416.76	A	**317.19**	**159.10**	300.17	150.59			3
8	44.05	893.56	447.28	876.53	438.77	875.55	438.28	921.56	461.28	904.53	452.77	903.55	452.28	A	**246.16**	123.58	229.13	115.07			2
9	**129.11**													R	**175.12**	88.06	**158.09**	79.55			1

MS/MS Fragmentation of **GKGGKGLGKGGAKR**

Found in **P62805**, Histone H4

Match to Query 4805: 1504.845448 from(753.430000,2+)

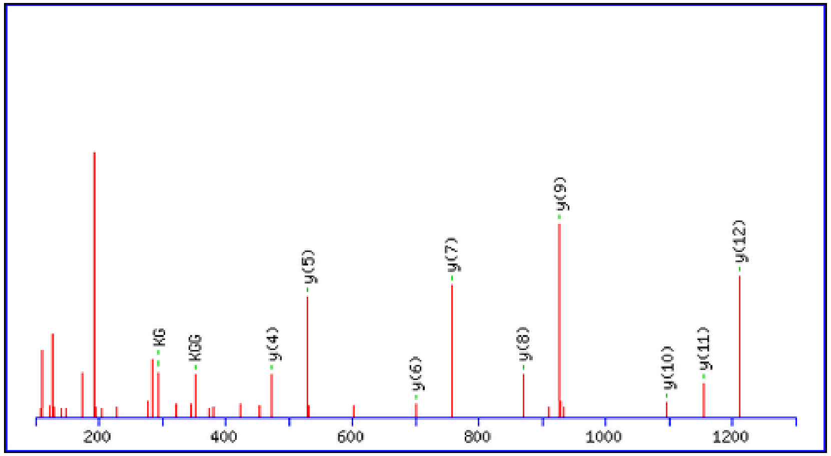

H4K5bz

Monoisotopic mass of neutral peptide Mr(calc): 1504.85
Variable modifications:
K2 : Benzoyl-D5 (K)
K5 : Acetyl (K)
K9 : Acetyl (K)
K13 : Acetyl (K)
Ions Score: 86 **Expect:** 7.8e-006
Matches (Bold Red): 17/266 fragment ions using 14 most inten

#	Immon.	a	a^{++}	a^{*}	a^{*++}	b	b^{++}	b^{*}	b^{*++}	Seq.	y	y^{++}	y^{*}	y^{*++}	#
1	30.03	30.03	15.52			58.03	29.52			G					14
2	210.16	267.19	134.10	250.16	125.58	**295.18**	148.09	278.15	139.58	K	1448.84	724.92	1431.81	716.41	13
3	30.03	324.21	162.61	307.18	154.09	**352.20**	176.61	335.18	168.09	G	**1211.69**	606.35	1194.66	597.83	12
4	30.03	381.23	191.12	364.20	182.61	409.22	205.12	392.20	196.60	G	**1154.66**	577.84	1137.64	569.32	11
5	143.12	551.33	276.17	534.31	267.66	579.33	290.17	562.30	281.66	K	**1097.64**	549.32	1080.62	540.81	10
6	30.03	608.36	304.68	591.33	296.17	636.35	318.68	619.32	310.17	G	**927.54**	464.27	910.51	455.76	9
7	86.10	721.44	361.22	704.41	352.71	749.44	375.22	732.41	366.71	L	**870.52**	435.76	853.49	427.25	8
8	30.03	778.46	389.73	761.44	381.22	806.46	403.73	789.43	395.22	G	**757.43**	379.22	740.40	370.71	7
9	143.12	948.57	474.79	931.54	466.27	976.56	488.78	959.54	480.27	K	**700.41**	350.71	683.38	342.20	6
10	30.03	1005.59	503.30	988.56	494.78	1033.58	517.30	1016.56	508.78	G	**530.30**	265.66	513.28	257.14	5
11	30.03	1062.61	531.81	1045.58	523.30	1090.61	545.81	1073.58	537.29	G	**473.28**	237.15	456.26	228.63	4
12	44.05	1133.65	567.33	1116.62	558.81	1161.64	581.32	1144.62	572.81	A	416.26	208.63	399.24	200.12	3
13	143.12	1303.75	652.38	1286.73	643.87	1331.75	666.38	1314.72	657.86	K	345.22	173.12	328.20	164.60	2
14	129.11									R	175.12	88.06	158.09	79.55	1

MS/MS Fragmentation of **GKGGKGLGKGGAKR**
Found in **P62805**, Histone H4
Match to Query 4808: 1504.849184 from(753.431868,2+)

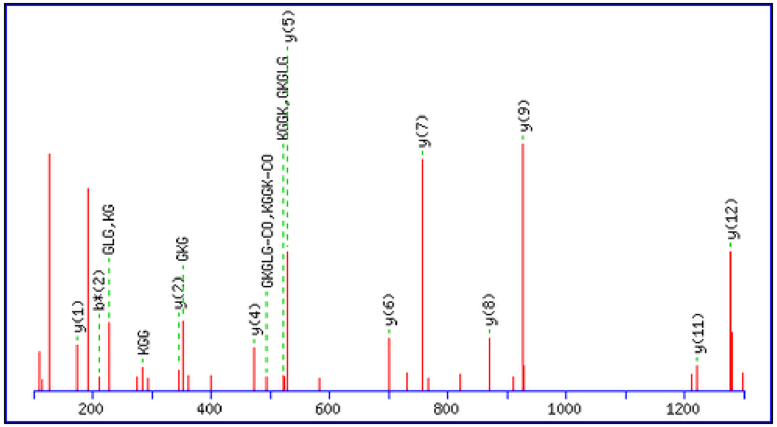

H4K8bz

Monoisotopic mass of neutral peptide Mr(calc): 1504.85
Variable modifications:
K2 : Acetyl (K)
K5 : Benzoyl-D5 (K)
K9 : Acetyl (K)
K13 : Acetyl (K)
Ions Score: 70 **Expect:** 0.00035
Matches (Bold Red): 28/260 fragment ions using 26 most inter

#	Immon.	a	a++	a*	a*++	b	b++	b*	b*++	Seq.	y	y++	y*	y*++	#
1	30.03	30.03	15.52			58.03	29.52			G					14
2	143.12	200.14	100.57	183.11	92.06	**228.13**	114.57	**211.11**	106.06	K	1448.84	724.92	1431.81	716.41	13
3	30.03	257.16	129.08	240.13	120.57	**285.16**	143.08	268.13	134.57	G	**1278.73**	639.87	1261.71	631.36	12
4	30.03	314.18	157.59	297.16	149.08	342.18	171.59	325.15	163.08	G	**1221.71**	611.36	1204.68	602.85	11
5	210.16	551.33	276.17	534.31	267.66	579.33	290.17	562.30	281.66	K	1164.69	582.85	1147.66	574.34	10
6	30.03	608.36	304.68	591.33	296.17	636.35	318.68	619.32	310.17	G	**927.54**	464.27	910.51	455.76	9
7	86.10	721.44	361.22	704.41	352.71	749.44	375.22	732.41	366.71	L	**870.52**	435.76	853.49	427.25	8
8	30.03	778.46	389.73	761.44	381.22	806.46	403.73	789.43	395.22	G	**757.43**	379.22	740.40	370.71	7
9	143.12	948.57	474.79	931.54	466.27	976.56	488.78	959.54	480.27	K	**700.41**	350.71	683.38	342.20	6
10	30.03	1005.59	503.30	988.56	494.78	1033.58	517.30	1016.56	508.78	G	**530.30**	265.66	513.28	257.14	5
11	30.03	1062.61	531.81	1045.58	523.30	1090.61	545.81	1073.58	537.29	G	**473.28**	237.15	456.26	228.63	4
12	44.05	1133.65	567.33	1116.62	558.81	1161.64	581.32	1144.62	572.81	A	416.26	208.63	399.24	200.12	3
13	143.12	1303.75	652.38	1286.73	643.87	1331.75	666.38	1314.72	657.86	K	**345.22**	173.12	328.20	164.60	2
14	129.11									R	**175.12**	88.06	158.09	79.55	1

MS/MS Fragmentation of **GKGGKGLGKGGAKR**

Found in **P62805**, Histone H4

Match to Query 4809: 1504.849930 from(753.432241,2+)

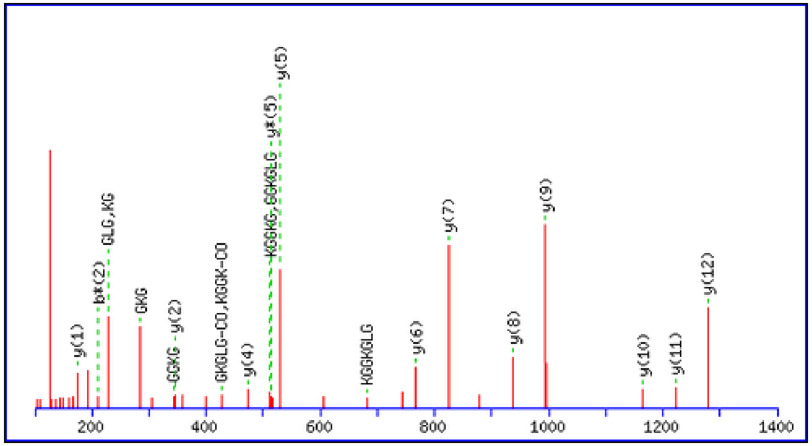

H4K12bz

Monoisotopic mass of neutral peptide Mr(calc): 1504.85
Variable modifications:
K2 : Acetyl (K)
K5 : Acetyl (K)
K9 : Benzoyl-D5 (K)
K13 : Acetyl (K)
Ions Score: 92 **Expect:** 2e-006
Matches (Bold Red): 33/260 fragment ions using 29 most in

#	Immon.	a	a++	a*	a*++	b	b++	b*	b*++	Seq.	y	y++	y*	y*++	#
1	30.03	30.03	15.52			58.03	29.52			G					14
2	143.12	200.14	100.57	183.11	92.06	**228.13**	114.57	**211.11**	106.06	K	1448.84	724.92	1431.81	716.41	13
3	30.03	257.16	129.08	240.13	120.57	**285.16**	143.08	268.13	134.57	G	**1278.73**	639.87	1261.71	631.36	12
4	30.03	314.18	157.59	297.16	149.08	**342.18**	171.59	325.15	163.08	G	**1221.71**	611.36	1204.68	602.85	11
5	143.12	484.29	242.65	467.26	234.13	**512.28**	256.65	495.26	248.13	K	**1164.69**	582.85	1147.66	574.34	10
6	30.03	541.31	271.16	524.28	262.64	569.30	**285.16**	552.28	276.64	G	**994.58**	497.80	977.56	489.28	9
7	86.10	654.39	327.70	637.37	319.19	**682.39**	341.70	665.36	333.18	L	**937.56**	469.28	920.54	460.77	8
8	30.03	711.41	356.21	694.39	347.70	739.41	370.21	722.38	361.70	G	**824.48**	412.74	807.45	404.23	7
9	210.16	948.57	474.79	931.54	466.27	976.56	488.78	959.54	480.27	K	**767.46**	384.23	750.43	375.72	6
10	30.03	1005.59	503.30	988.56	494.78	1033.58	517.30	1016.56	508.78	G	**530.30**	265.66	**513.28**	257.14	5
11	30.03	1062.61	531.81	1045.58	523.30	1090.61	545.81	1073.58	537.29	G	**473.28**	237.15	456.26	228.63	4
12	44.05	1133.65	567.33	1116.62	558.81	1161.64	581.32	1144.62	572.81	A	416.26	208.63	399.24	200.12	3
13	143.12	1303.75	652.38	1286.73	643.87	1331.75	666.38	1314.72	657.86	K	**345.22**	173.12	328.20	164.60	2
14	129.11									R	**175.12**	88.06	158.09	79.55	1

MS/MS Fragmentation of **GGKGLGKGGAKR**
Found in **P62805**, Histone H4
Match to Query 3699: 1305.754890 from(653.884721,2+)

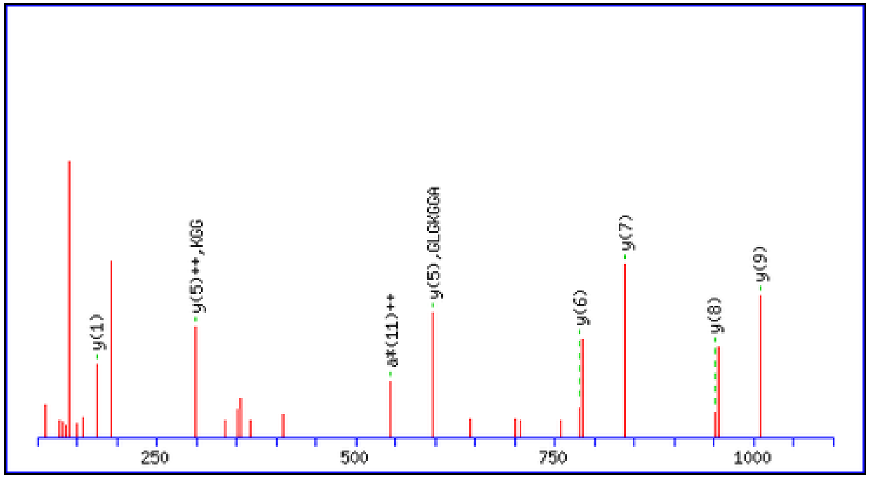

H4K16bz

Monoisotopic mass of neutral peptide Mr(calc): 1305.76
Variable modifications:
K3 : Propionyl (K)
K7 : Propionyl (K)
K11 : Benzoyl-D5 (K)
Ions Score: 28 **Expect:** 3.2
Matches (Bold Red): 13/210 fragment ions using 19 m

#	Immon.	a	a^{++}	a^{*}	a^{*++}	b	b^{++}	b^{*}	b^{*++}	Seq.	y	y^{++}	y^{*}	y^{*++}	#
1	30.03	30.03	15.52			58.03	29.52			G					12
2	30.03	87.06	44.03			115.05	58.03			G	1249.74	625.37	1232.72	616.86	11
3	157.13	271.18	136.09	254.15	127.58	**299.17**	150.09	282.14	141.58	K	1192.72	596.86	1175.69	588.35	10
4	30.03	328.20	164.60	311.17	156.09	356.19	178.60	339.17	170.09	G	**1008.60**	504.80	991.57	496.29	9
5	86.10	441.28	221.14	424.26	212.63	469.28	235.14	452.25	226.63	L	**951.58**	476.29	934.55	467.78	8
6	30.03	498.30	249.66	481.28	241.14	526.30	263.65	509.27	255.14	G	**838.49**	419.75	821.47	411.24	7
7	157.13	682.42	341.72	665.40	333.20	710.42	355.71	693.39	347.20	K	**781.47**	391.24	764.45	382.73	6
8	30.03	739.45	370.23	722.42	361.71	767.44	384.22	750.41	375.71	G	**597.35**	**299.18**	580.32	290.67	5
9	30.03	796.47	398.74	779.44	390.22	824.46	412.73	807.44	404.22	G	540.33	270.67	523.30	262.16	4
10	44.05	867.50	434.26	850.48	425.74	895.50	448.25	878.47	439.74	A	483.31	242.16	466.28	233.64	3
11	210.16	1104.66	552.83	1087.63	**544.32**	1132.65	566.83	1115.63	558.32	K	412.27	206.64	395.24	198.13	2
12	129.11									R	**175.12**	88.06	158.09	79.55	1

MS/MS Fragmentation of **GKQGGKAR**
Found in **Q7L7L0**, Histone H2A type 3
Match to Query 1527: 951.529902 from(476.772227,2+)

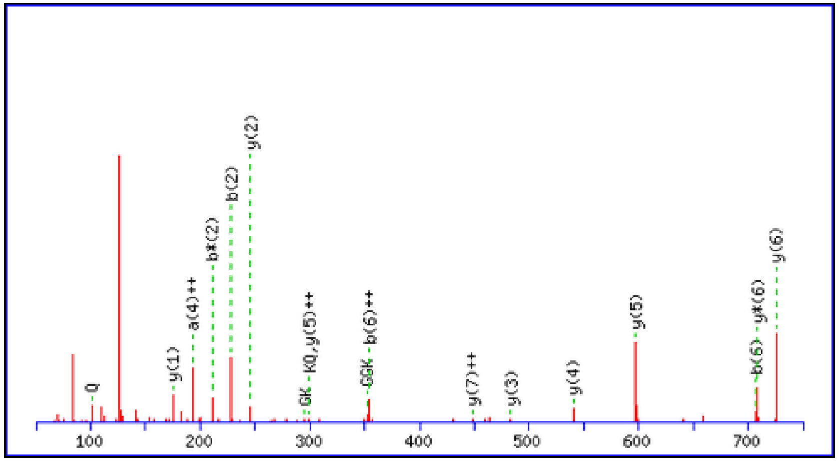

H2AK9bz

Monoisotopic mass of neutral peptide Mr(calc): 951.53
Variable modifications:
K2 : Acetyl (K)
K6 : Benzoyl-D5 (K)
Ions Score: 34 **Expect:** 0.27
Matches (Bold Red): 18/117 fragment ions using 31 most intense peaks

#	Immon.	a	a++	a*	a*++	b	b++	b*	b*++	Seq.	y	y++	y*	y*++	#
1	30.03	30.03	15.52			58.03	29.52			G					8
2	143.12	200.14	100.57	183.11	92.06	**228.13**	114.57	**211.11**	106.06	K	895.52	**448.26**	878.49	439.75	7
3	**101.07**	328.20	164.60	311.17	156.09	356.19	178.60	339.17	170.09	Q	**725.41**	363.21	**708.38**	354.70	6
4	30.03	385.22	**193.11**	368.19	184.60	413.21	207.11	396.19	198.60	G	**597.35**	**299.18**	580.32	290.67	5
5	30.03	442.24	221.62	425.21	213.11	470.24	235.62	453.21	227.11	G	**540.33**	270.67	523.30	262.16	4
6	210.16	679.39	340.20	662.37	331.69	**707.39**	**354.20**	690.36	345.68	K	**483.31**	242.16	466.28	233.64	3
7	44.05	750.43	375.72	733.40	367.21	778.43	389.72	761.40	381.20	A	**246.16**	123.58	229.13	115.07	2
8	129.11									R	**175.12**	88.06	158.09	79.55	1

MS/MS Fragmentation of **AKAKSR**
Found in **Q7L7L0**, Histone H2A type 3
Match to Query 746: 824.491360 from(413.252956,2+)

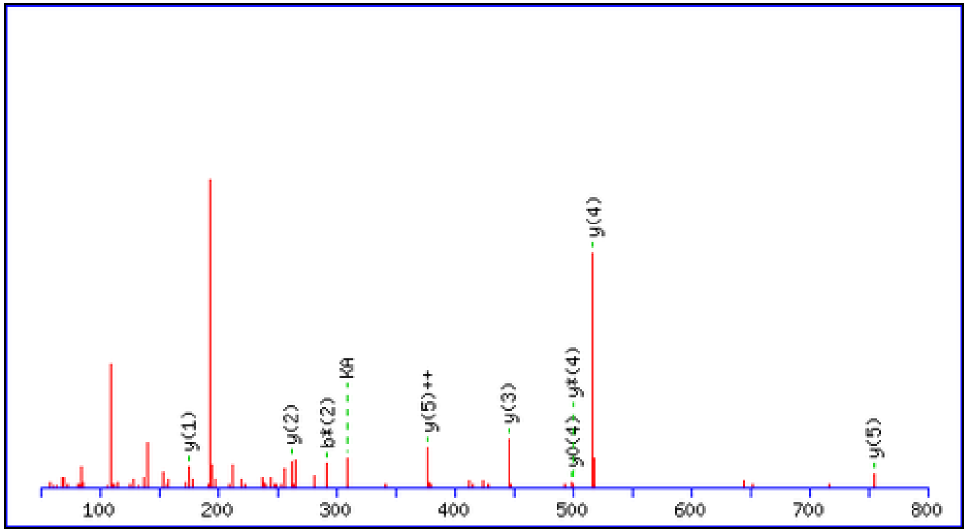

H2AK13bz

Monoisotopic mass of neutral peptide Mr(calc): 824.49
Variable modifications:
K2 : Benzoyl-D5 (K)
K4 : Propionyl (K)
Ions Score: 24 **Expect:** 2.2
Matches (Bold Red): 11/86 fragment ions using 24 most intense peaks

#	Immon.	a	a^{++}	a^*	a^{*++}	a^0	a^{0++}	b	b^{++}	b^*	b^{*++}	b^0	b^{0++}	Seq.	y	y^{++}	y^*	y^{*++}	y^0	y^{0++}	#
1	44.05	44.05	22.53					72.04	36.53					A							6
2	210.16	281.20	141.10	264.18	132.59			**309.20**	155.10	**292.17**	146.59			K	**754.46**	**377.73**	737.44	369.22	736.45	368.73	5
3	44.05	352.24	176.62	335.21	168.11			380.23	190.62	363.21	182.11			A	**517.31**	259.16	**500.28**	250.64	**499.30**	250.15	4
4	157.13	536.36	268.68	519.33	260.17			564.36	282.68	547.33	274.17			K	**446.27**	223.64	429.25	215.13	428.26	214.63	3
5	60.04	623.39	312.20	606.37	303.69	605.38	303.19	651.39	326.20	634.36	317.68	633.38	317.19	S	**262.15**	131.58	245.12	123.07	244.14	122.57	2
6	129.11													R	**175.12**	88.06	158.09	79.55			1

MS/MS Fragmentation of **PEPAKSAPAPK**

Found in **P62807**, Histone H2B type 1-C/E/F/G/I

Match to Query 3568: 1200.654972 from(601.334762,2+)

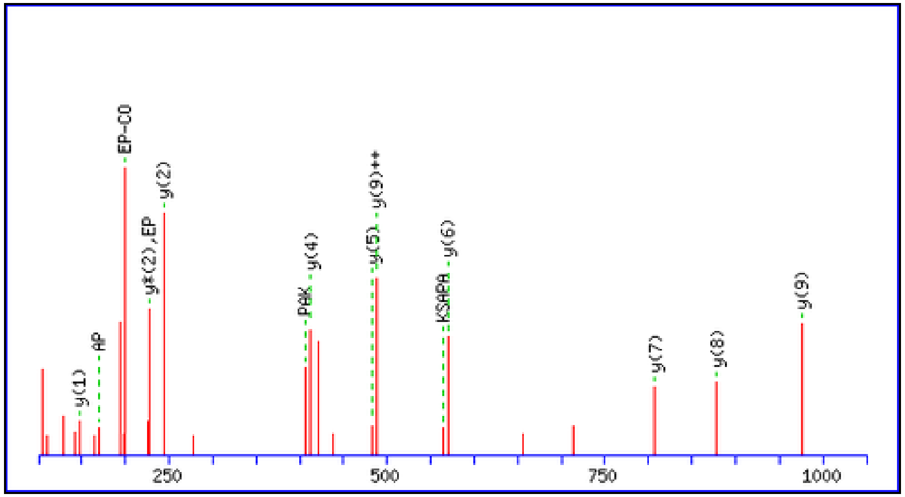

H2BK5bz

Monoisotopic mass of neutral peptide Mr(calc): 1200.66
Variable modifications:
K5 : Benzoyl-D5 (K)
Ions Score: 57 **Expect:** 0.0022
Matches (Bold Red): 22/221 fragment ions using 23 most intense peaks

#	Immon.	a	a^{++}	a^{*}	a^{*++}	a^{0}	a^{0++}	b	b^{++}	b^{*}	b^{*++}	b^{0}	b^{0++}	Seq.	y	y^{++}	y^{*}	y^{*++}	y^{0}	y^{0++}	#
1	70.07	70.07	35.54					98.06	49.53					P							11
2	102.05	**199.11**	100.06			181.10	91.05	**227.10**	114.05			209.09	105.05	E	1104.61	552.81	1087.58	544.30	1086.60	543.80	10
3	70.07	296.16	148.58			278.15	139.58	324.16	162.58			306.14	153.58	P	**975.57**	**488.29**	958.54	479.77	957.56	479.28	9
4	44.05	367.20	184.10			349.19	175.10	395.19	198.10			377.18	189.09	A	**878.51**	439.76	861.49	431.25	860.50	430.76	8
5	210.16	604.35	302.68	587.32	294.17	586.34	293.67	632.35	316.68	615.32	308.16	614.33	307.67	K	**807.48**	404.24	790.45	395.73	789.47	395.24	7
6	60.04	691.38	346.19	674.36	337.68	673.37	337.19	719.38	360.19	702.35	351.68	701.37	351.19	S	**570.32**	285.67	553.30	277.15	552.31	276.66	6
7	44.05	762.42	381.71	745.39	373.20	744.41	372.71	790.41	395.71	773.39	387.20	772.40	386.71	A	**483.29**	242.15	466.27	233.64			5
8	70.07	859.47	430.24	842.45	421.73	841.46	421.23	887.47	444.24	870.44	435.72	869.46	435.23	P	**412.26**	206.63	395.23	198.12			4
9	44.05	930.51	465.76	913.48	457.24	912.50	456.75	958.50	479.76	941.48	471.24	940.49	470.75	A	315.20	158.10	298.18	149.59			3
10	70.07	1027.56	514.28	1010.54	505.77	1009.55	505.28	1055.56	528.28	1038.53	519.77	1037.55	519.28	P	**244.17**	122.59	**227.14**	114.07			2
11	101.11													K	**147.11**	74.06	130.09	65.55			1

MS/MS Fragmentation of **SAPAPKK**

Found in **P62807**, Histone H2B type 1-C/E/F/G/I

Match to Query 767: 806.469552 from(404.242052,2+)

H2BK11bz

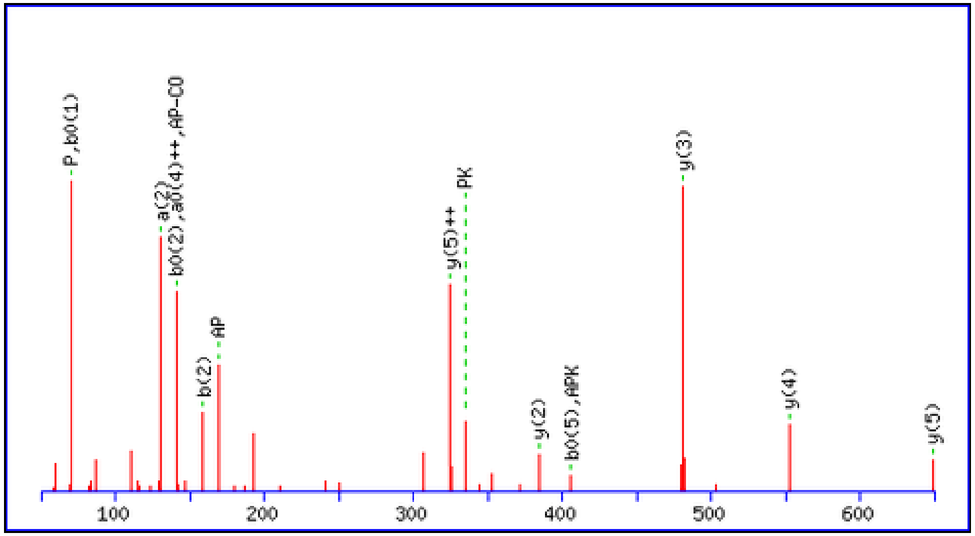

Monoisotopic mass of neutral peptide Mr(calc): 806.47
Variable modifications:
K6 : Benzoyl-D5 (K)
Ions Score: 35 **Expect:** 0.22
Matches (Bold Red): 21/103 fragment ions using 13 most intense peaks

#	Immon.	a	a^{++}	a^{*}	a^{*++}	a^{0}	a^{0++}	b	b^{++}	b^{*}	b^{*++}	b^{0}	b^{0++}	Seq.	y	y^{++}	y^{*}	y^{*++}	#
1	60.04	60.04	30.53			42.03	21.52	88.04	44.52			**70.03**	35.52	S					7
2	44.05	**131.08**	66.04			113.07	57.04	**159.08**	80.04			**141.07**	71.04	A	720.45	360.73	703.42	352.21	6
3	**70.07**	228.13	114.57			210.12	105.57	256.13	128.57			238.12	119.56	P	**649.41**	**325.21**	632.38	316.69	5
4	44.05	299.17	150.09			281.16	**141.08**	327.17	164.09			309.16	155.08	A	**552.36**	276.68	535.33	268.17	4
5	**70.07**	396.22	198.62			378.21	189.61	424.22	212.61			**406.21**	203.61	P	**481.32**	241.16	464.29	232.65	3
6	210.16	633.38	317.19	616.35	308.68	615.37	308.19	661.37	331.19	644.35	322.68	643.36	322.18	K	**384.27**	192.64	367.24	184.12	2
7	101.11													K	147.11	74.06	130.09	65.55	1

MS/MS Fragmentation of **KAVTKAQK**

Found in **P62807**, Histone H2B type 1-C/E/F/G/I

Match to Query 2491: 1023.612270 from(512.813411,2+)

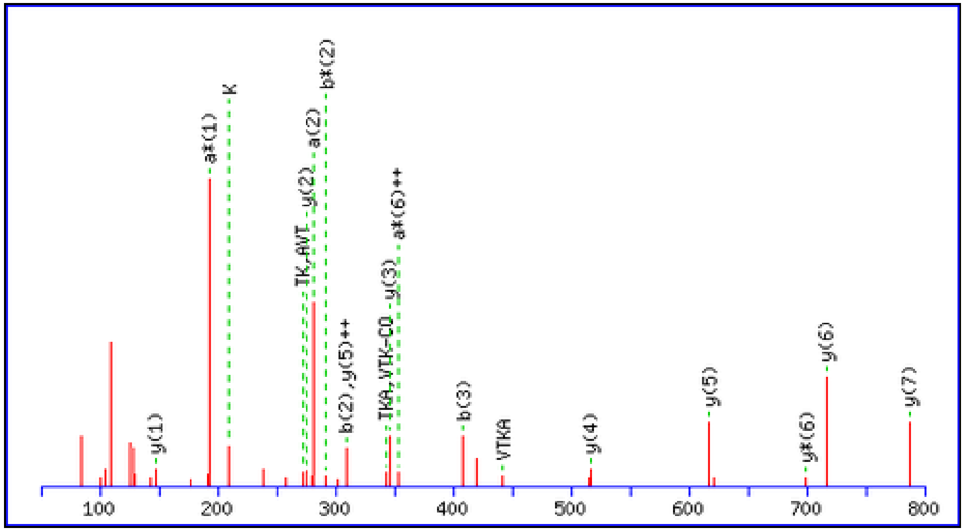

H2BK16bz

Monoisotopic mass of neutral peptide Mr(calc): 1023.61

Variable modifications:

K1 : Benzoyl-D5 (K)

K5 : Acetyl (K)

Ions Score: 47 **Expect:** 0.009

Matches (Bold Red): 23/144 fragment ions using 29 most intense peaks

#	Immon.	a	a^{++}	a^{*}	a^{*++}	a^{0}	a^{0++}	b	b^{++}	b^{*}	b^{*++}	b^{0}	b^{0++}	Seq.	y	y^{++}	y^{*}	y^{*++}	y^{0}	y^{0++}	#
1	**210.16**	**210.16**	105.59	**193.14**	97.07			238.16	119.58	221.13	111.07			K							8
2	44.05	**281.20**	141.10	264.18	132.59			**309.20**	155.10	**292.17**	146.59			A	**787.47**	394.24	770.44	385.72	769.46	385.23	7
3	72.08	380.27	190.64	363.24	182.13			**408.27**	204.64	391.24	196.12			V	**716.43**	358.72	**699.40**	350.21	698.42	349.71	6
4	74.06	481.32	241.16	464.29	232.65	463.31	232.16	509.31	255.16	492.29	246.65	491.30	246.15	T	**617.36**	**309.18**	600.34	300.67	599.35	300.18	5
5	143.12	651.42	326.22	634.40	317.70	633.41	317.21	679.42	340.21	662.39	331.70	661.41	331.21	K	**516.31**	258.66	499.29	250.15			4
6	44.05	722.46	361.73	705.43	**353.22**	704.45	352.73	750.46	375.73	733.43	367.22	732.45	366.73	A	**346.21**	173.61	329.18	165.09			3
7	101.07	850.52	425.76	833.49	417.25	832.51	416.76	878.51	439.76	861.49	431.25	860.50	430.76	Q	**275.17**	138.09	258.14	129.58			2
8	101.11													K	**147.11**	74.06	130.09	65.55			1

MS/MS Fragmentation of **KAVTKAQK**

Found in **P62807**, Histone H2B type 1-C/E/F/G/I

Match to Query 2191: 981.601444 from(491.807998,2+)

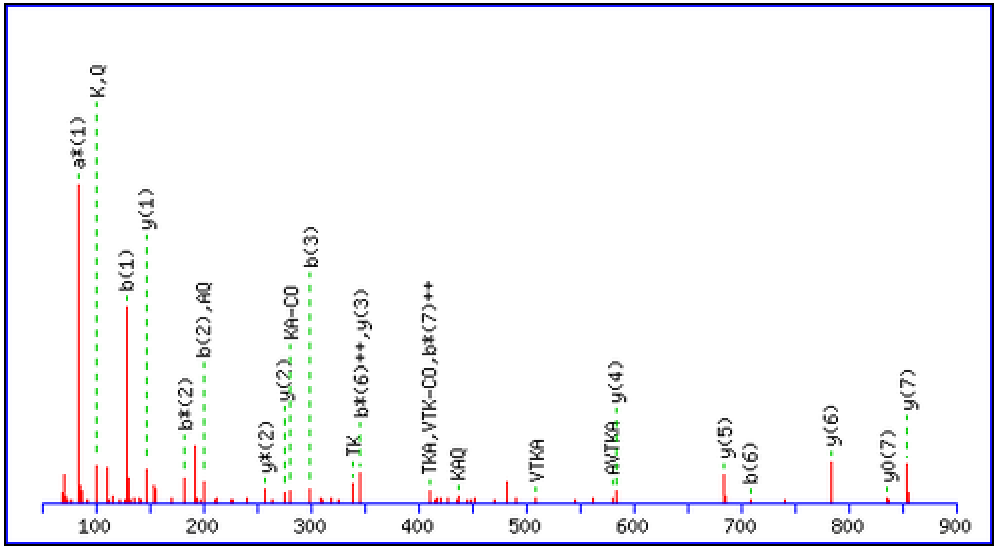

H2BK20bz

Monoisotopic mass of neutral peptide Mr(calc): 981.60

Variable modifications:

K5 : Benzoyl-D5 (K)

Ions Score: 45 **Expect:** 0.013

Matches (Bold Red): 31/143 fragment ions using 36 most intense peaks

#	Immon.	a	a^{++}	a^{*}	a^{*++}	a^{0}	a^{0++}	b	b^{++}	b^{*}	b^{*++}	b^{0}	b^{0++}	Seq.	y	y^{++}	y^{*}	y^{*++}	y^{0}	y^{0++}	#
1	**101.11**	**101.11**	51.06	**84.08**	42.54			**129.10**	65.05	112.08	56.54			K							8
2	44.05	172.14	86.58	155.12	78.06			**200.14**	100.57	**183.11**	92.06			A	**854.51**	427.76	837.49	419.25	**836.50**	418.76	7
3	72.08	271.21	136.11	254.19	127.60			**299.21**	150.11	282.18	141.59			V	**783.48**	392.24	766.45	383.73	765.47	383.24	6
4	74.06	372.26	186.63	355.23	178.12	354.25	177.63	400.26	200.63	383.23	192.12	382.24	191.63	T	**684.41**	342.71	667.38	334.19	666.40	333.70	5
5	210.16	609.41	305.21	592.39	296.70	591.40	296.20	637.41	319.21	620.38	310.69	619.40	310.20	K	**583.36**	292.18	566.33	283.67			4
6	44.05	680.45	340.73	663.42	332.22	662.44	331.72	**708.45**	354.73	691.42	**346.21**	690.43	345.72	A	**346.21**	173.61	329.18	165.09			3
7	**101.07**	808.51	404.76	791.48	396.24	790.50	395.75	**836.50**	418.76	819.48	**410.24**	818.49	409.75	Q	**275.17**	138.09	**258.14**	129.58			2
8	**101.11**													K	**147.11**	74.06	130.09	65.55			1

Supplementary Figure 4. MS/MS spectra of histone D_5-K_{bz}-containing peptides in HepG2 cells treated with D_5-SB. Annotated MS/MS spectra are provided for each detected D_5-K_{bz} peptide. In each spectra, the major ions were found to be the y- and b-ions (C- and N-terminal fragments cleaved across the peptide bond, respectively).

a

Site	Spectra count in HepG2 Cells		Spectra count in RAW cells	
	K_{ac}	K_{bz}	K_{ac}	K_{bz}
**H2AK5**	2	2	4	4
**H2AK8**	2	2	2	2
**H3K9**	5	5	2	2
**H3K14**	7	4	5	3
**H3K18**	14	3	7	5
**H3K23**	30	5	13	2
**H4K5**	24	15	16	13
**H4K8**	24	3	18	7
**H4K12**	24	17	32	12

b

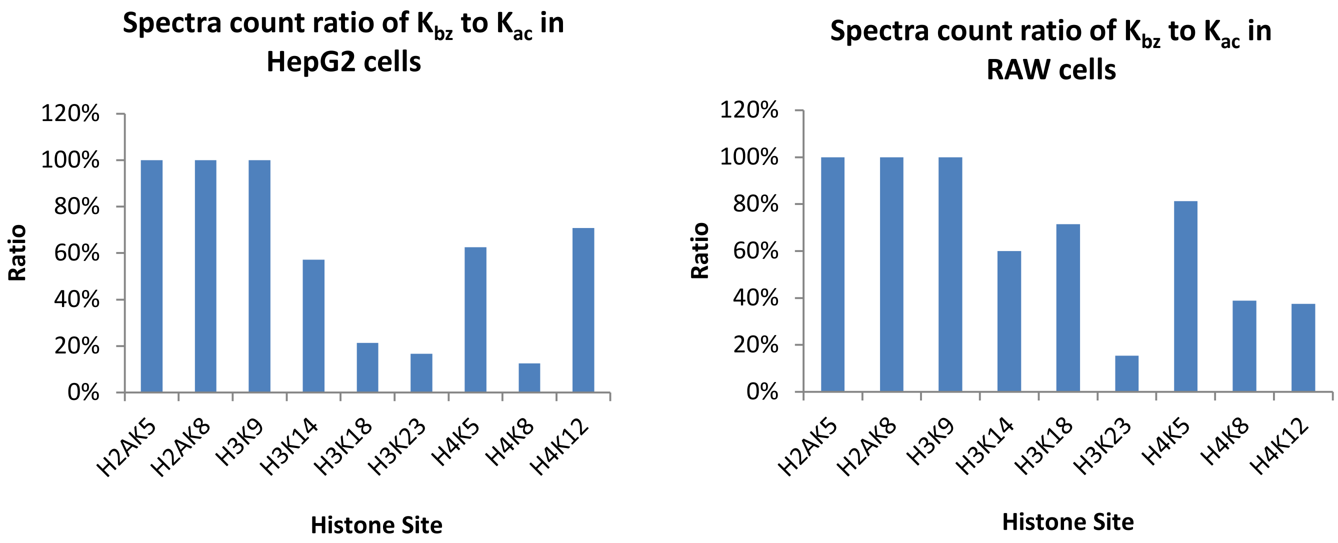

Supplementary Figure 5. Relative abundance of histone K_{bz} and K_{ac} sites in SB treated (5 mM for 24 h) HepG2 and RAW cells.

(a) Spectra count number of K_{bz} and K_{ac} peptides in HepG2 and RAW cells.

(b) Comparison of K_{bz} and K_{ac} abundance in HepG2 (left) and RAW (right) cells.

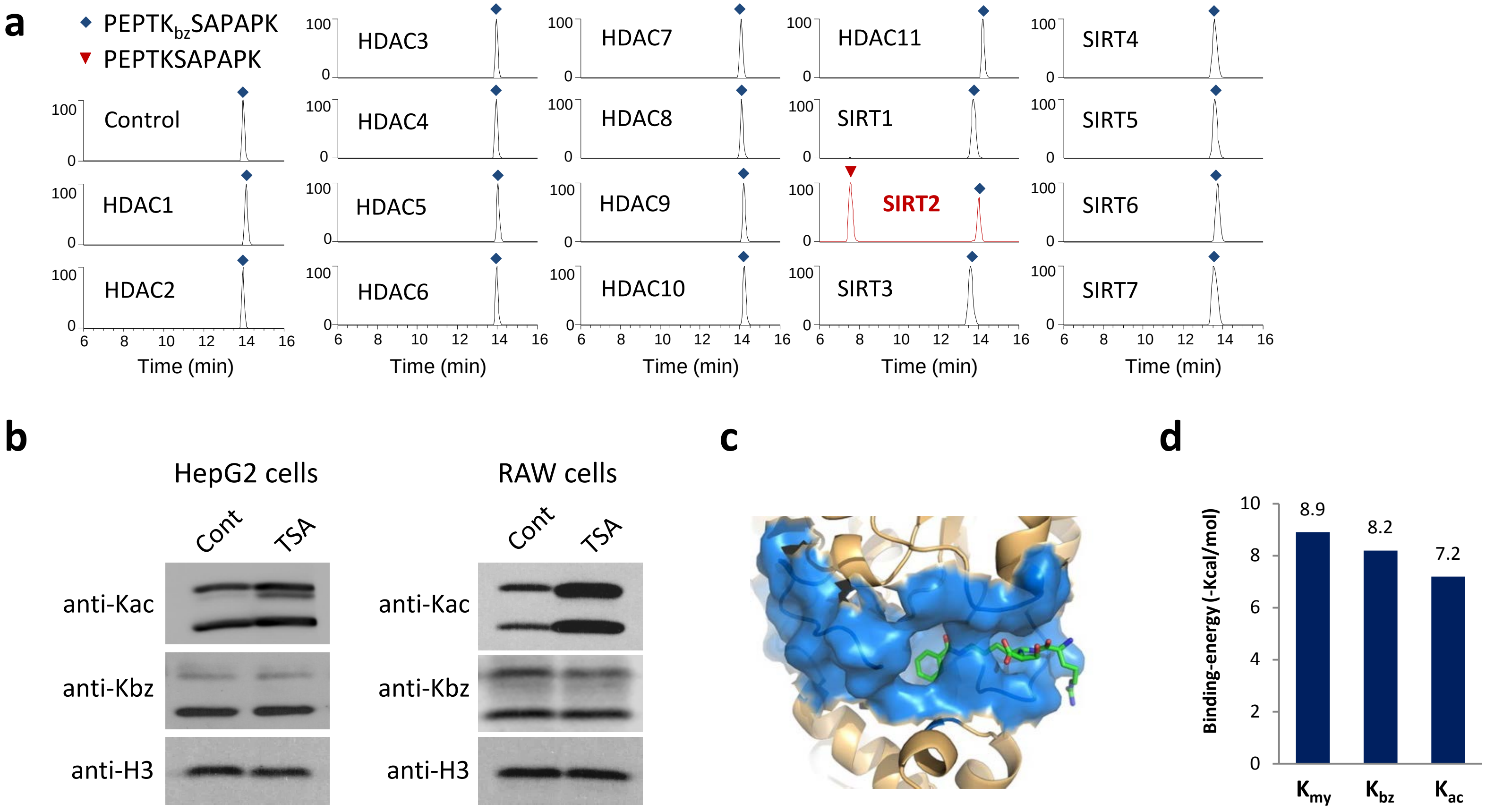

Supplementary Figure 6. Identification of K_{bz} deacylase.

(a) Lysine debenzoylation activity screening of HDACs *in vitro* by HPLC-MS/MS with K_{bz} peptide PEPTK$_{bz}$SAPAPK.

(b) Inhibition of class I/II HDACs increased Kac levels but not Kbz levels of core histones in cells. HepG2 and RAW cells were treated with a class I/II HDAC inhibitor TSA (0.5 µM) for 18 hours. Histones were extracted and Kac/Kbz levels were analyzed by immuno-blotting with indicated antibodies.

(c) Binding conformation of K_{bz} peptide with SIRT2 crystal structure (PDB ID 4Y6L).

(d) Binding energy between SIRT2 and acylated peptides. K_{my} represents the myristoyl peptide.

Original western blot from Figure 2d

Original western blot from Figure 4b

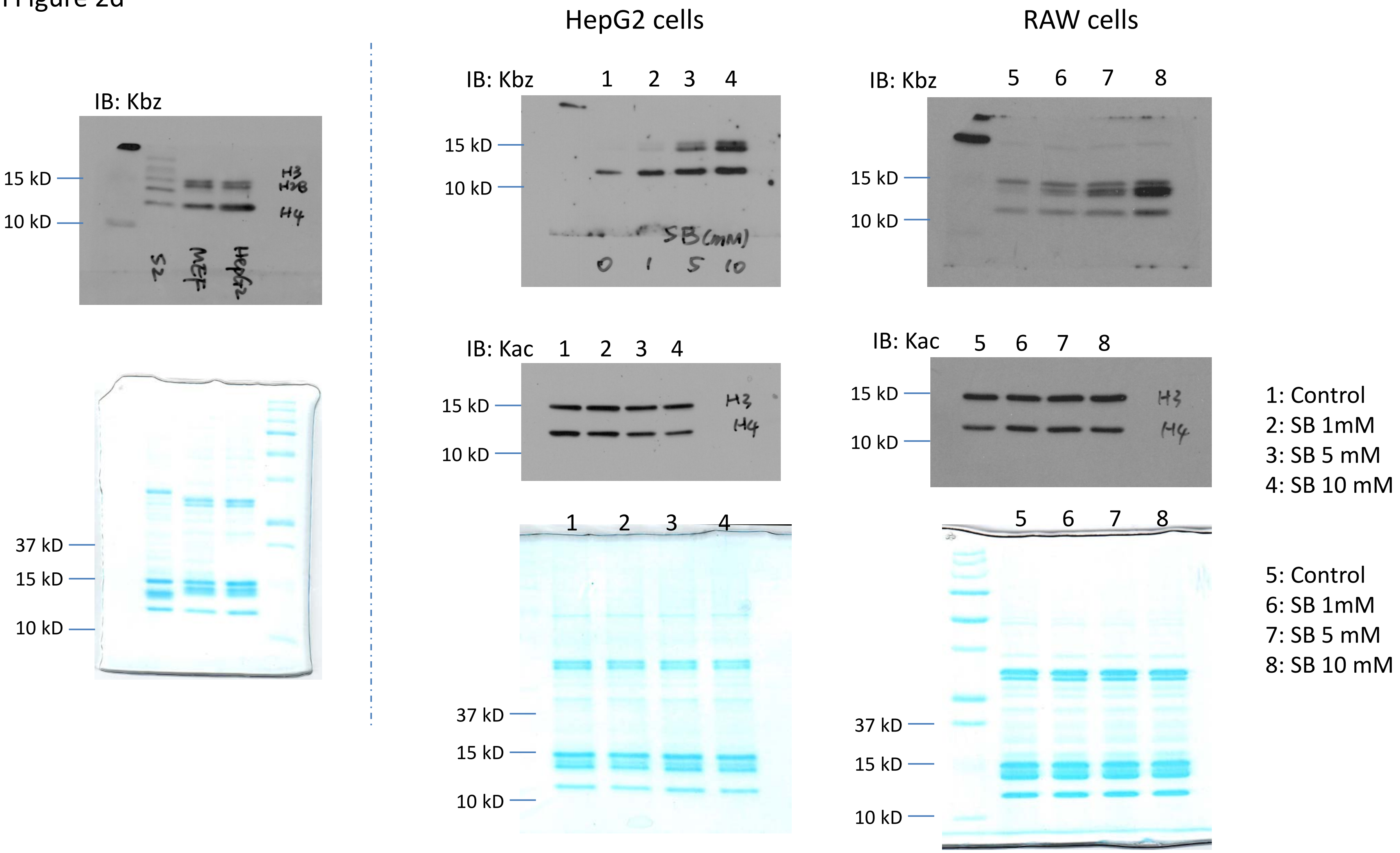

Supplementary Figure 7. Uncropped western blot and SDS-page from Figures 2d and 4b.

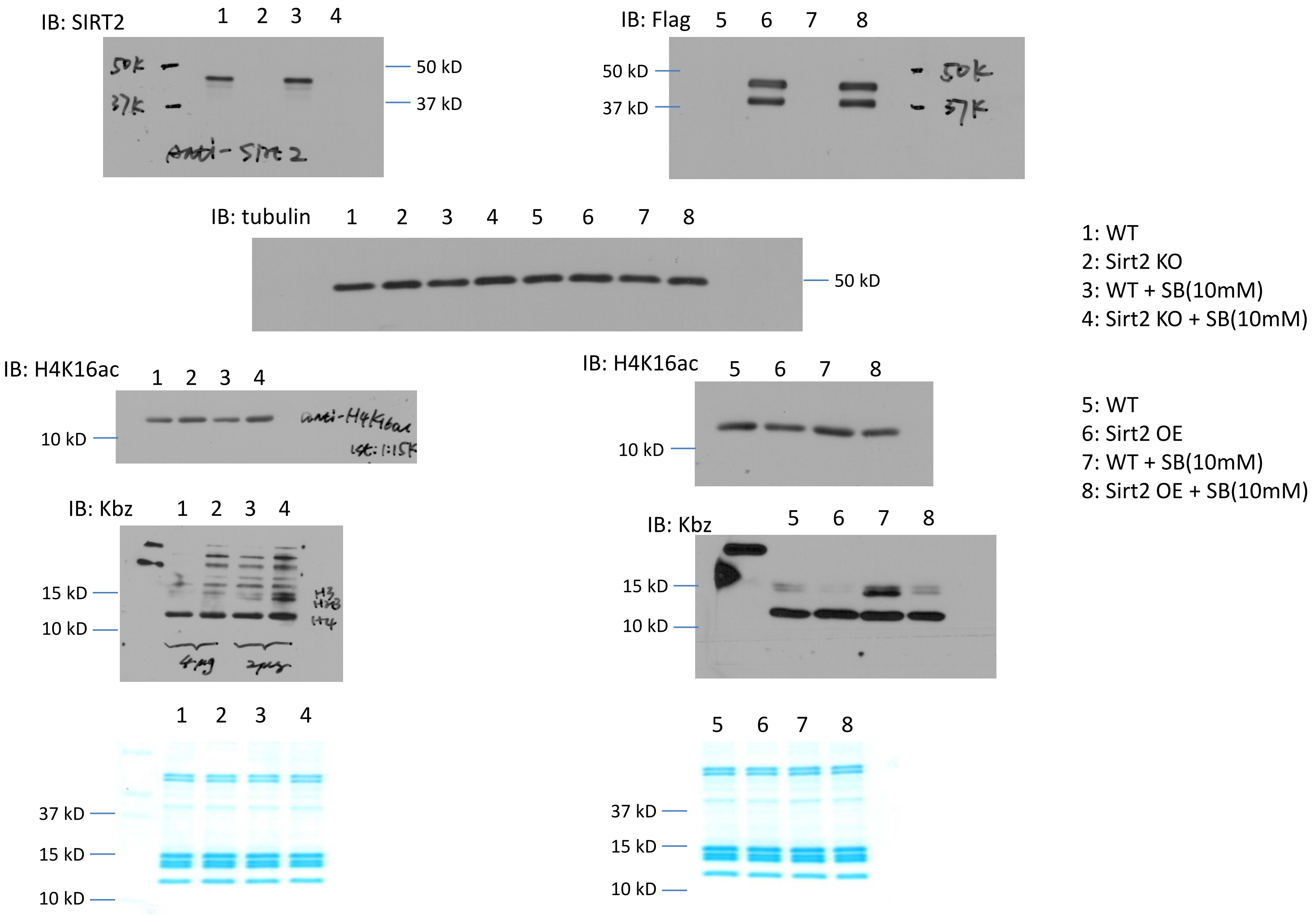

Supplementary Figure 8. Uncropped western blot and SDS-page from Figure 5.

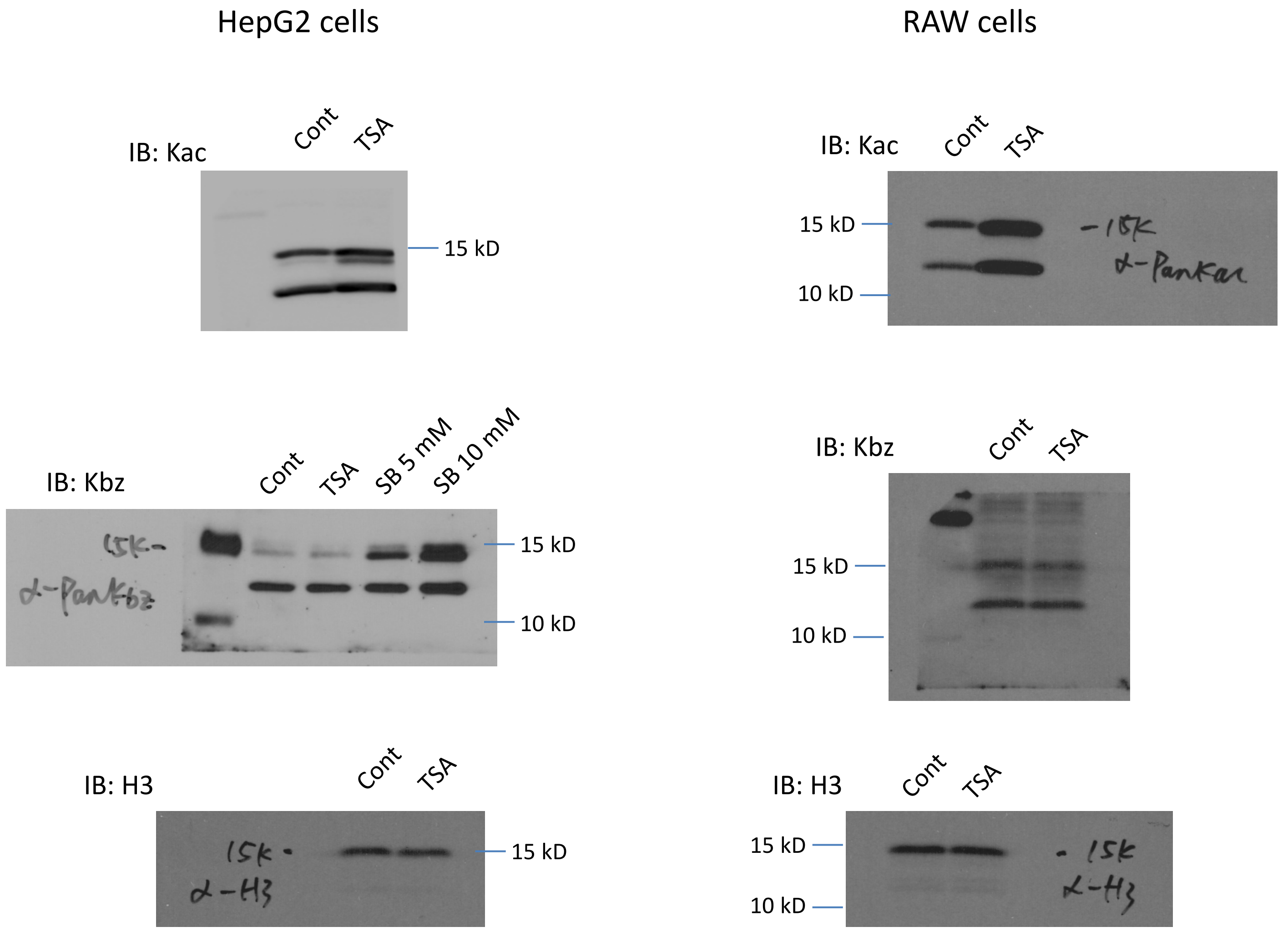

Supplementary Figure 9. Uncropped western blot from Supplementary Figure 6.
