# Supplementary material for: Lysine benzoylation is a histone mark regulated by SIRT2
Source: Nat Commun. 2018 Aug 28;9:3374. doi: 10.1038/s41467-018-05567-w (PMC6113264; doi:10.1038/s41467-018-05567-w)
Supplement: Supplementary file 3 — Description of Additional Supplementary Files [file 41467_2018_5567_MOESM3_ESM.pdf]

Description of Additional Supplementary Files

File Name: Supplementary Data 1
Description: Differentially expressed genes for GSEA analysis.

File Name: Supplementary Data 2
Description: Gene list for KEGG pathway analysis.
